# Pan-Cancer Analysis of Prognostic and Immune Infiltrates for CXCs

**DOI:** 10.3390/cancers13164153

**Published:** 2021-08-18

**Authors:** Long Li, Wenchao Yao, Sen Yan, Xianghui Dong, Zhenyi Lv, Qingxu Jing, Qiang Wang, Biao Ma, Chenjun Hao, Dongbo Xue, Dawei Wang

**Affiliations:** 1 Department of General Surgery, The First Affiliated Hospital of Harbin Medical University, Harbin 150001, China; ll007@hrbmu.edu.cn (L.L.); yaowenchao@hrbmu.edu.cn (W.Y.); lvzhenyi@hrbmu.edu.cn (Z.L.); jingqingxu@hrbmu.edu.cn (Q.J.); wangqiang@hrbmu.edu.cn (Q.W.); mabiao@hrbmu.edu.cn (B.M.); haochenjun@hrbmu.edu.cn (C.H.); 2 Key Laboratory of Hepatosplenic Surgery, Ministry of Education, The First Affiliated Hospital of Harbin Medical University, Harbin 150001, China; 3 Key Laboratory of Cardiac Diseases and Heart Failure, Harbin Medical University, Harbin 150001, China; yansen@hrbmu.edu.cn; 4 Key Laboratory of Pathology, The First Affiliated Hospital of Harbin Medical University, Harbin 150001, China; 2018020839@hrbmu.edu.cn

**Keywords:** multiple tumor, prognosis, genetic alteration, immune infiltration, single-cell sequencing

## Abstract

**Simple Summary:**

CXCs are important genes that regulate inflammation and tumor metastasis. While there are many studies with a focus on individual CXCs, few present a pan-cancer analysis of the whole CXC family. Our results indicate that CXCs are a potential therapeutic target in a variety of tumors and a potential prognostic marker that could improve the survival of cancer patients and the accuracy of prognosis. Meanwhile, we found that CXCs may be involved in diseases caused by intestinal flora.

**Abstract:**

Background: CXCs are important genes that regulate inflammation and tumor metastasis. However, the expression level, prognosis value, and immune infiltration of CXCs in cancers are not clear. Methods: Multiple online datasets were used to analyze the expression, prognosis, and immune regulation of CXCs in this study. Network analysis of the Amadis database and GEO dataset was used to analyze the regulation of intestinal flora on the expression of CXCs. A mouse model was used to verify the fact that intestinal bacterial dysregulation can affect the expression of CXCs. Results: In the three cancers, multiple datasets verified the fact that the mRNA expression of this family was significantly different; the mRNA levels of CXCL3, 8, 9, 10, 14, and 17 were significantly correlated with the prognosis of three cancers. CXCs were correlated with six types of immuno-infiltrating cells in three cancers. Immunohistochemistry of clinical samples confirmed that the expression of CXCL8 and 10 was higher in three cancer tissues. Animal experiments have shown that intestinal flora dysregulation can affect CXCL8 and 10 expressions. Conclusion: Our results further elucidate the function of CXCs in cancers and provide new insights into the prognosis and immune infiltration of breast, colon, and pancreatic cancers, and they suggest that intestinal flora may influence disease progression through CXCs.

## 1. Introduction

Cancer is a major worldwide public health problem, causing an estimated total of 9 million deaths in 2016 (World Health Statistics, 2020). Although significant survival benefits have been achieved in recent years because of early detection, screening, and treatment methods, improving overall survival (OS) remains a challenge in the clinic. Tumor metastasis is an important factor that correlates with a poor prognosis of cancer. Metastasis depends on the tumor microenvironment, which is a complex system composed of tumor cells, lymphocytes, cancer-associated fibroblasts, and other cells. These cells secrete cytokines, including CXCs, which promote the growth of tumor cells and tumor angiogenesis as well as contribute to tumor metastasis [1].

CXC chemokines (C corresponds to cysteine and X denotes any amino acid) are a subfamily of the chemokines. CXC chemokines comprise CXCL1–17 and are divided into two structurally distinct groups according to with or without a three-amino-acid sequence, glutamic acid–leucine–arginine (ELR), in their primary structure [2]. In general, angiogenic activity is usually enhanced by ELR-positive chemokines and inhibited by ELR-negative chemokines [3]. The ELR + CXC chemokines include CXCL1–3, CXCL5–8, and CXCL17, and the ELR chemokines include CXCL4, CXCL9–14, and CXCL16. The biological functions of CXC chemokines have been described in various cancers. High levels of CXCL1, 2, 3, 5, and 13 in renal cell carcinoma patients were associated with a poor prognosis [4]. CXCL2 recruits myeloid-derived suppressor cells and is a biomarker of short OS in bladder cancer [5]. Overexpression of CXCL3 can enhance the proliferation and migration in uterine cervical cancer [6]. CXCL8 is the crucial chemokine responsible for gastric cancer metastasis and indicates poor clinical outcomes [7]. Therefore, CXC chemokines may be potential biomarkers for evaluating the prognosis of tumors and may become new treatment targets.

Breast cancer, colon cancer, and pancreatic cancer are malignant tumors that easily metastasize and progress, and they are characterized by poor prognosis and high mortality. Previous researches have demonstrated that aberrant expression of some members of the CXC family is associated with tumor development in breast, colorectal, and pancreatic cancer. For instance, a high level of CXCL1 in ER-negative breast cancer cells stimulates invasion [8], and up-regulation of the CXCR4/CXCL12 axis is associated with lymph node metastasis in invasive breast carcinoma [9]. Tumor-associated macrophages could secret CXCL1, which promotes breast cancer metastasis by the NF-κB/SOX4 pathway [4]. CXCs influence colorectal cancer progression by regulating inflammation and antitumor immunity [10]. CXCL1 plays the most important role in the metastasis of colorectal cancer [11]. Dawei et al. demonstrated that the CXCL12/CXCR4 axis protects colorectal cancer cells against radiotherapy by enhancing survival [12]. Members of the CXCs are present in the pancreatic tumor microenvironment and play a critical role in the regulation of pancreatic cancer progression [13]. However, there are no comprehensive analyses of CXCs in multiple tumors. The regulatory factors of CXC expression, as well as the association between CXC expression, prognosis, and infiltration of immune cells, remain unclear.

In this study, we used data from several large public databases to perform comprehensive bioinformatics analysis of various aspects of CXC functions in multiple cancers, including expression, prognosis, immune infiltration, genetic changes, and functional analysis. We collected clinical samples and found that CXCs were differentially expressed between tumor tissues and normal tissues. With the aid of network analysis tools of the Amadis database, we found that there could be an association between CXCL8, *Fusobacterium nucleatum*, and human diseases (including inflammatory bowel disease and colon cancer). To validate this association, an azoxymethane (AOM)/dextran sulfate sodium (DSS)-induced colitis-associated cancer (CAC) mouse model was used. Our results proved that intestinal bacterial dysregulation could affect the expression of CXCL8 and 10.

This study provides guidance for the clinical treatment of three cancers and reveals the interaction between flora and colon cancer. 

## 2. Materials and Methods

### 2.1. Oncomine Analysis

The Oncomine database (https://www.oncomine.org, accessed on 11 February 2021) is a database for sequencing data analysis [14]. In our study, transcriptional expressions of different CXCs members between different cancer tissues and their corresponding adjacent normal control samples were obtained from the Oncomine database. Difference in transcriptional expression was compared by Student’s *t*-test. Cut-off of *p* value and fold change were as follows: *p* value: 0.05; fold change: 2.0; gene rank: 10%.

### 2.2. TISCH Analysis

Tumor Immune Single-cell Hub (TISCH, http://tisch.comp-genomics.org/, accessed on 21 February 2021), a database focusing on the tumor microenvironment (TME), provides single-cell level cell-type annotation [15]. In the present study, we evaluated the expression level of CXCs in each subgroup of cells in the three cancer datasets and analyzed the interrelationship between the level and tumor stage. GSEA enrichment analysis and visualization of inflammatory pathways were also performed.

### 2.3. GEPIA Analysis

Gene Expression Profiling Interactive Analysis (GEPIA, http://gepia.cancer-pku.cn/, accessed on 25 February 2021) is a web-based tool to deliver fast and customizable functionalities based on 9736 tumors and 8587 normal samples from GTEx database and The Cancer Genome Atlas (TCGA, https://portal.gdc.cancer.gov, accessed on 1 March 2021) [16]. In our study, tumor/normal differential expression analysis and pathological stages were obtained from them. Difference in transcriptional expression was compared by Student’s *t*-test, and *p* < 0.05 was considered statically significant.

### 2.4. Kaplan–Meier Plotter

Kaplan–Meier plotter (https://www.kmplot.com, accessed on 5 March 2021) is a database that contains gene expression data and survival information of breast cancer patients. The prognostic value of mRNA expression was analyzed using this database [17]. To analyze the OS of patients with three aforementioned malignancies, samples of patients were segregated into two groups (high-expression group and low-expression group). These groups were assessed by a Kaplan–Meier survival plot, with the hazard ratio (HR) with 95% confidence intervals (CIs) and a log-rank *p* value. Only the JeSet best probe set was selected.

### 2.5. OncoLnc Dataset

OncoLnc (http://www.oncolnc.org/, accessed on 6 March 2021) is a tool for interactively exploring survival correlations, which contains survival data for 21 cancer studies performed by TCGA. The PDAC and COAD patients were divided into two groups; we assessed the OS of these groups by using Kaplan–Meier plots and log-rank *p* value; and the cut-off criterion was log-rank *p* value < 0.05. 

### 2.6. TRRUST Dataset

TRRUST v2 (https://www.grnpedia.org/trrust/, accessed on 7 March 2021) is a dataset that provides the transcription factor (TF) of target genes and the regulatory network between them. It includes 8444 TF–target regulatory relationships of 800 human TFs and 6552 TF–target regulatory relationships of 828 mouse TFs. These data are derived from 11,237 PubMed articles that describe small-scale experimental studies of transcriptional regulations [18].

### 2.7. KnockTF Dataset

KnockTF (http://www.licpathway.net/KnockTF/index.html, accessed on 7 March 2021) is a database providing available resources of human gene expression profile datasets, which are associated with TF knockdown/knockout. The database annotates TFs and their targets in a tissue/cell-type-specific way [19]. 

### 2.8. MiRWalk Dataset

MiRWalk 2.0 (http://mirwalk.umm.uni-heidelberg.de, accessed on 8 March 2021), an open source platform, can predict and validate miRNA-binding sites of genes from humans, mice, rats, dogs, and cows. The core of miRWalk is the TarPmiR (random-forest-based approach) that can predict miRNA target sites of the transcript sequence. 

### 2.9. CBioPortal Dataset

CBioPortal (https://www.cbioportal.org, accessed on 8 March 2021), a comprehensive database, provides analysis and visualization functions to process multi-tumor genomics data [20]. Based on data in TCGA, genetic alterations and co-expression of CXCs were obtained from cBioPortal. Protein expression z scores (RPPA) and mRNA expression z scores (RNA Seq V2 RSEM) were obtained using a z score threshold of 2.0. 

### 2.10. STRING Dataset

STRING 11.0 Dataset (https://string-db.org/, accessed on 8 March 2021) collects and integrates PPI (protein–protein interaction) data from public sources and predicts potential functions [21]. A CXCs–PPI network analysis was used to inquire about the interactions. The visualization of those networks was achieved by Cytoscape v.3.6.

### 2.11. GO (Gene Ontology) and KEGG (Kyoto Encyclopedia of Genes and Genomes) Analysis

GO and KEGG analyses of 66 proteins interacting with CXCs found in the STRING database were performed using the Database for Annotation, Visualization, and Integrated Discovery (DAVID, https://david.ncifcrf.gov/summary.jsp, accessed on 9 March 2021) [22]. GO analysis can reveal the potential functional roles of CXCs, including biological processes (BP), cellular components (CC), and molecular functions (MF), while KEGG analysis can define the pathways related to CXCs.

### 2.12. TIMER Analysis

The Tumor Immune Estimation Resource (TIMER 1.0, https://cistrome.shinyapps.io/timer/, accessed on 10 March 2021) is a database that focuses on analyzing tumor-infiltrating immune cells throughout 32 kinds of malignancies from TCGA [23]. We used the gene module to inquiry correlations between CXCL expression and abundance of tumor-infiltrating immune cells by Spearman’s correlation, which include CD8 + T cells, CD4 + T cells, macrophages, neutrophils, B cells, and dendritic cells.

### 2.13. Amadis

Amadis is a database that provides experimentally supported microbiota–disease associations [24]. With aid of Amadis’s network analysis tools, we found that there could be an association between CXCL8, *Fusobacterium nucleatum*, and human diseases (including inflammatory bowel disease and colon cancer).

### 2.14. Bacterial Culture

*Fusobacterium nucleatum (F. nucleatum*) strain ATCC 25586, which was purchased from American Type Culture Collection (ATCC, Manassas, VA, USA), was cultured in brain heart infusion (BHI) broth at 37 °C under anaerobic conditions.

### 2.15. Mice

The animal experiments obtained permission through the Animal Ethics and Welfare Committee (AEWC) of the First Affiliated Hospital of Harbin Medical University. C57BL/6J wild-type (WT) mice were purchased from Beijing Vital River Laboratory Animal Technology Co. Ltd. (Beijiing, China). Six- to eight-week-old female C57BL/6J mice aged 6–8 weeks were housed in standard specific pathogen-free conditions. 

The mice were injected with a single intraperitoneal(i.p.) injection of the AOM (10 mg/kg). One week later, they were given three cycles of 2% DSS treatment (1 week per cycle). The mice were treated with *F. nucleatum* (1 × 10^9^ CFU) by gavage from a fortnight before AOM injection until sacrifice. During the DSS intervention, *F. nucleatum* administration was suspended. The negative control mice were gavage-fed with PBS only. Intragastric gavage administration was carefully carried out with the animal immobilized, using a gavage needle appropriate for mice. Before bacterial intragastric administration, mice were fed with broad-spectrum antibiotics (BSA) in the drinking water for 5 days to ensure the consistency of regular microbiota and facilitate *F. nucleatum* colonization. The DAI (disease activity index) and body weight were observed daily.

### 2.16. Western Blot

Western blots were performed according to standard protocols. A 12% SDS-PAGE gel was used to separate total proteins extracted from mice colon tissue. Then, proteins were transferred onto polyvinylidene fluoride membranes. The membranes were incubated with primary antibodies for CXCL8 (Novus) and CXCL10 (Affinity) overnight at 4 °C after blocking with 5% non-fat dry milk in PBST. Anti-GAPDH (Beyotime, 1:1000) was used as the control. Each experiment has been repeated at least three times.

### 2.17. Enzyme-Linked Immunosorbent Assay (ELISA)

The mouse blood samples were centrifuged, serum was collected, and immediately cryopreserved in liquid nitrogen. According to the manufacturer’s instructions, the quantification of serum cytokine was carried out using the Quantibody^®^ Mouse CXCL10 ELISA Kit (RayBiotech, Norcross, GA, USA).

### 2.18. Histology and Immunohistochemistry (IHC)

Cancer tissue samples and paracancerous tissue samples were collected from the First Affiliated Hospital of Harbin Medical University. The studies obtained permission through the Ethics Committee of the First Affiliated Hospital of Harbin Medical University. Written informed consents were signed by patients/participants to participate in this study.

For histologic evaluation, formalin-fixed colon tissue sections were embedded in paraffin and cut (5 μm) for H&E staining or immunohistochemistry (IHC). For IHC assays, we deparaffinized the paraffin sections, inactivated endogenous enzymes, and thermally repaired antigens. These sections were stained with CXCL8 (Novus) and 10 (Affinity) antibodies, followed by a corresponding secondary antibody and a Streptavidin Biotin Complex kit (Boster BioEngineering, Wuhan, China). Stained slides were scanned by KFBIO. SlideViewer and quantified by Image-pro-plus software.

### 2.19. Statistical Analysis

All data were analyzed using SPSS 22.0 software (Chicago, IL, USA) by ordinary one-way analysis of variances with Tukey’s multiple comparisons. *p* < 0.05 was considered statistically significant.

## 3. Results

### 3.1. Analysis Process and Data Processing

**Figure 1:**
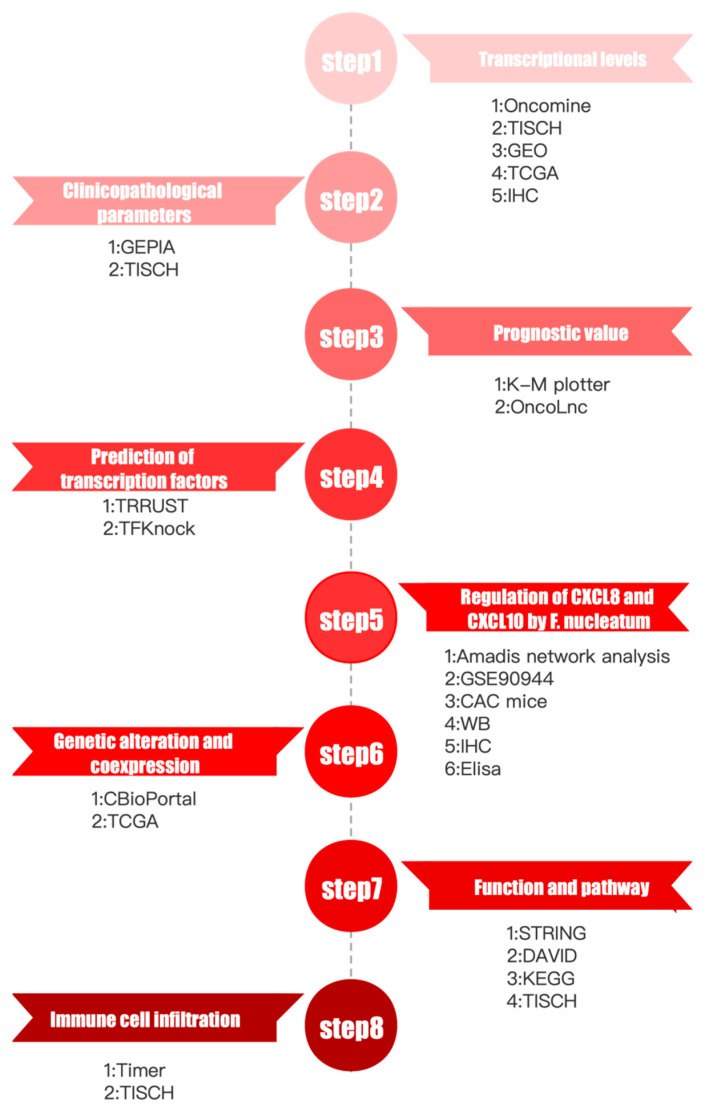
Analysis process and data processing of CXCs in 8 steps.

The analysis process is shown in Figure 1. The data used by this study are from TCGA datasets and Gene Expression Omnibus (GEO, https://www.ncbi.nlm.nih.gov/geo, accessed on 15 March 2021) datasets. We conducted a comprehensive analysis of CXCs in eight steps (Figure 1).

### 3.2. Transcriptional Levels of CXCs in Various Cancers

**Figure 2:**
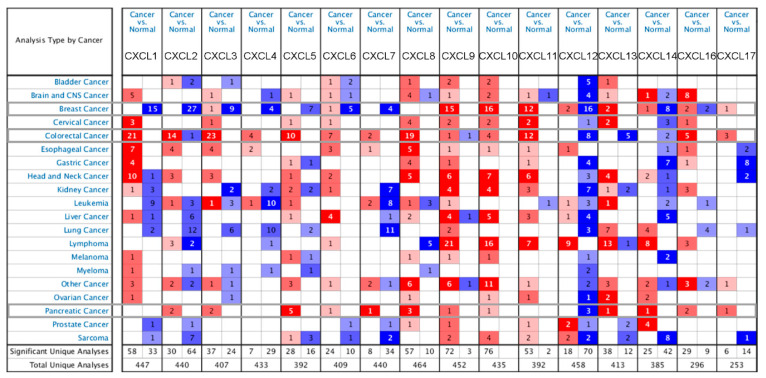
Transcriptional expression of CXCs in 20 different kinds of cancer diseases (ONCOMINE database). Difference in transcriptional expression was compared using Students’ *t*-test. Cut-off of *p* value and fold change were as follows: *p* value: 0.05; fold change: 2.0; gene rank: 10%; data type: mRNA.

First, we used the Oncomine database to analyze the differential expression levels of CXC transcripts in 20 types of cancer tissues versus the corresponding normal tissues. We found that each of the 16 genes of this family had approximately 400 unique analyses, except CXCL16 and 17. For these 16 genes, we identified cancer types with significant differences in the expression confirmed by multiple unique analyses (Figure 2).

CXCL1 was significantly expressed at high levels in 21 unique analyses in colon cancer and significantly expressed at low levels in 15 unique analyses in breast cancer. CXCL2 was significantly expressed at high levels in 14 unique analyses and significantly expressed at low levels in 27 unique analyses in breast cancer. CXCL3 was significantly expressed at high levels in 23 unique analyses in colon cancer. CXCL8 was significantly expressed at high levels in 19 unique analyses in colon cancer and 3 unique analyses in pancreatic cancer. CXCL9 was significantly expressed at high levels in 15 unique analyses in breast cancer and 21 unique analyses in lymphoma. CXCL10 was significantly expressed at high levels in 16 unique analyses in breast cancer and 16 unique analyses in lymphoma. There were 12 unique analyses with significantly high expression of CXCL11 in breast cancer and 12 unique analyses in colon cancer, and 16 unique analyses with significantly low expression of CXCL12 in breast cancer, and 3 unique analyses with significantly low expression in pancreatic cancer. According to these analyses, we found significant differences in the expression of the CXCs in BRCA, COAD, and PDAC; thus, we selected these three types of cancer for follow-up analysis.

In the TCGA datasets with more than 100 samples, invasive ductal breast cancer samples showed low expression of CXCL1, 2, 3, 12, and 14 (fold change > 2). The Curtis dataset of invasive ductal breast cancer showed significant differences in the expression of CXCL2, 8, 9, 10, 12, and 14 (fold change > 2) [25]. Colon cancer samples from the TCGA showed high expression of CXCL1, 3, 5, 6, and 11 and low expression of CXCL12 (fold change > 2); the Bittner poly-cancerous dataset confirmed the differential expression of CXCs in colon cancer and breast cancer. There were fewer samples of pancreatic cancer. In the Barretina dataset with 44 samples, the levels of CXCL2, 3, 5, and 16 (fold change > 2) were significantly higher [26], and the levels of CXCL3, 5, 8, 10, and 16 (fold change > 2) were significantly higher in the Badea and Pei datasets (Table 1) [27].

**Figure 3:**
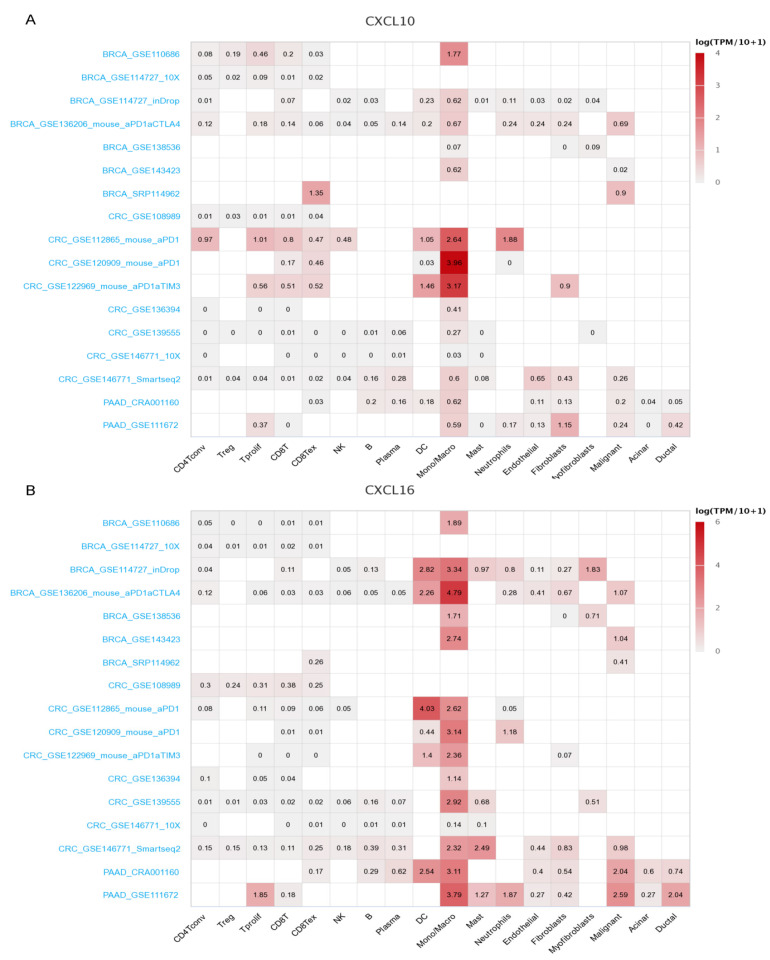
Expression levels of CXCL10 and 16 analysis by using TISCH in cell subpopulations of BRCA, COAD, and PDAC. Heatmap of CXCL10 (**A**) and 16 (**B**) expression in different cell subpopulations.

We used the TISCH database to analyze subpopulation distribution (Material S1) of 16 genes in single-cell sequencing datasets of breast, colon, and pancreatic cancers. Among them, CXCL10 and 16 were significantly increased in mononuclear/macrophage cells of the three cancers (Figure 3).

According to the number of cells detected in the dataset and the expression of CXC in each dataset, the breast cancer dataset BRCA_GSE114727_inDrop, colon cancer dataset CRC_GSE146771_10X, and pancreatic cancer dataset PAAD_CRA001160 were selected for further analysis.

### 3.3. mRNA and Protein Expression of CXCs in Three Kinds of Cancer

**Figure 4:**
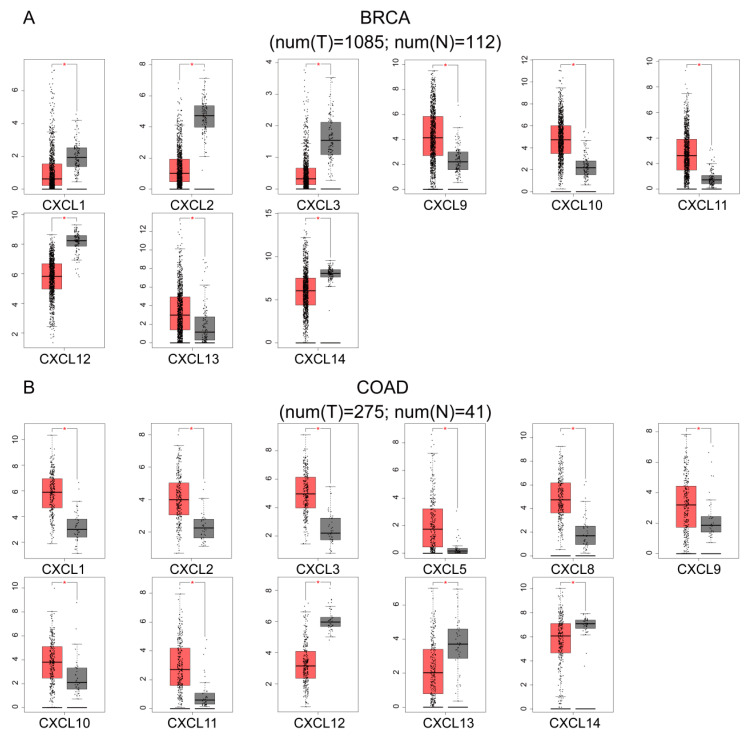

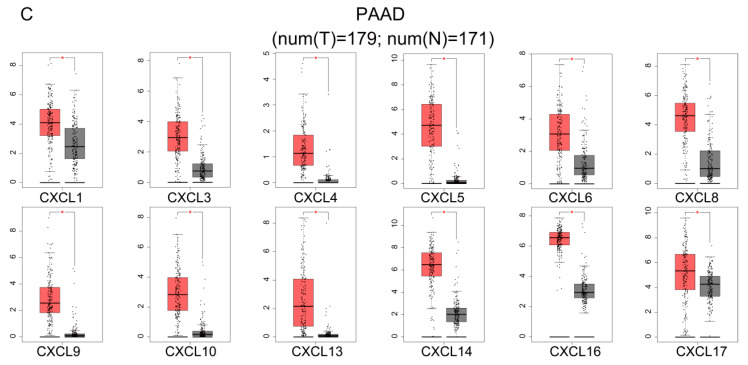
GEPIA analysis of the mRNA expression levels of CXCs in BRCA, COAD, and PDAC. Box plots of individual CXC expression in BRCA (**A**), COAD (**B**), and PDAC (**C**) tissue and normal tissues; *p* value ≤ 0.05.

Using the GEPIA dataset, we compared the expression of mRNAs in three types of cancer tissues versus normal tissues. The results showed that the expression of CXCL1, 2, 3, 12, and 14 in BRCA was lower in tumor tissues, and the expression of CXCL9, 10, 11, and 13 was higher than its expression in normal tissues; in COAD, the expression of CXCL12, 13 and 14 was lower than that in normal tissues, and the expression of CXCL1, 2, 3, 5, 8, 9, 10 and 11 was higher than that in normal tissues; in PDAC, the expression of CXCs was significantly higher than that in normal tissues, except CXCL2, 7, 11 and 12 (Figure 4).

In addition, we detected expression differences of CXCRs (CXC receptor) in three cancers. The results showed that there was no significant difference between normal tissue and cancer tissue, except for the fact that CXCR4 and 6 were less expressed in pancreatic cancer tissue (Material S2).

**Figure 5:**
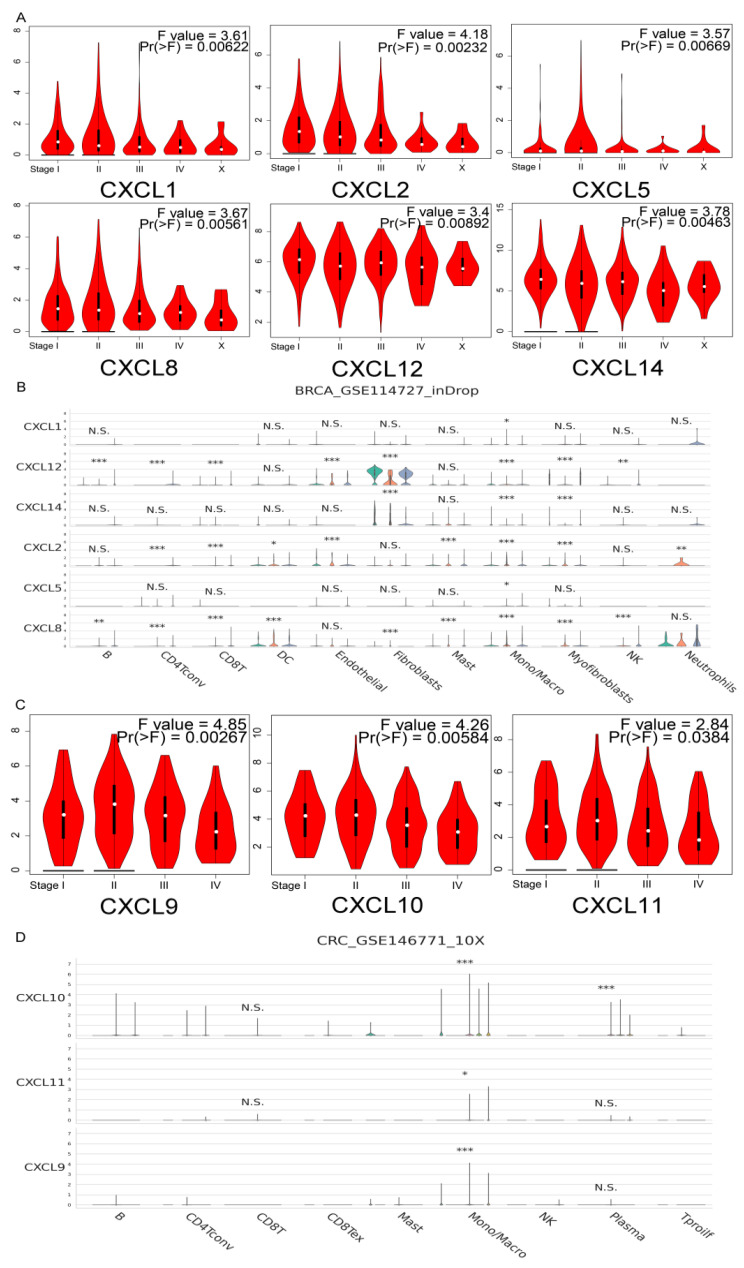

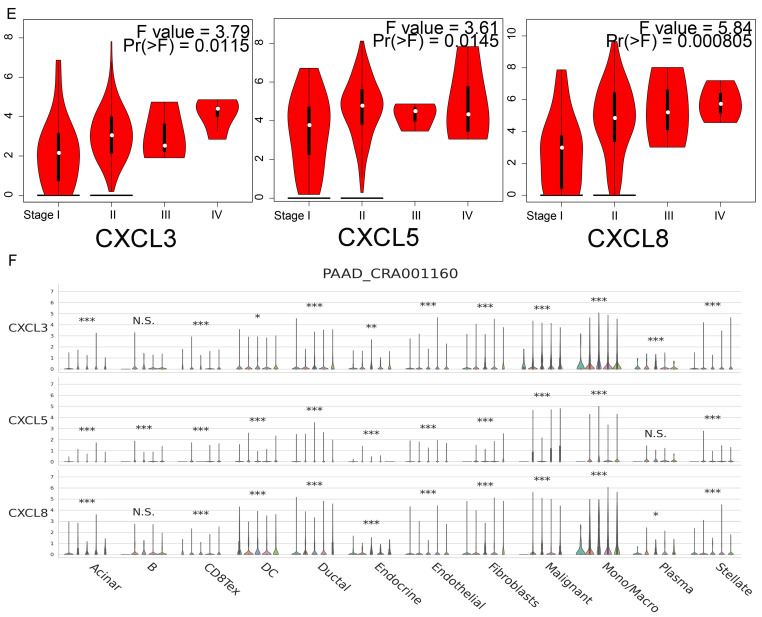
Correlation between CXC expression and tumor stage of BRCA (**A**,**B**), COAD (**C**,**D**), and PDAC (**E**,**F**) patients in GEPIA and TISCH.

We also analyzed the expression of CXCs in three types of cancers at various stages. The TISCH database was used to analyze the relationship between CXC expression and staging in different subsets of cells. In BRCA, the staging differences in CXCL1, 2, 5, 8, 12, and 14 were statistically significant (Figure 5A). Among them, CXCL2, 8, and 12 were generally significantly correlated with staging in each cell subgroup, and six genes were significantly correlated with staging in the mononuclear/macrophage subgroup (Figure 5B). In COAD, only the staging differences in CXCL9, 10, and 11 were statistically significant (Figure 5C) and were significantly associated with staging in the mononuclear/macrophage subpopulation (Figure 5D). In PDAC, the staging differences in CXCL3, 5, and 8 were statistically significant (Figure 5E) and were generally significantly associated with staging in all cell subsets (Figure 5F).

**Figure 6:**
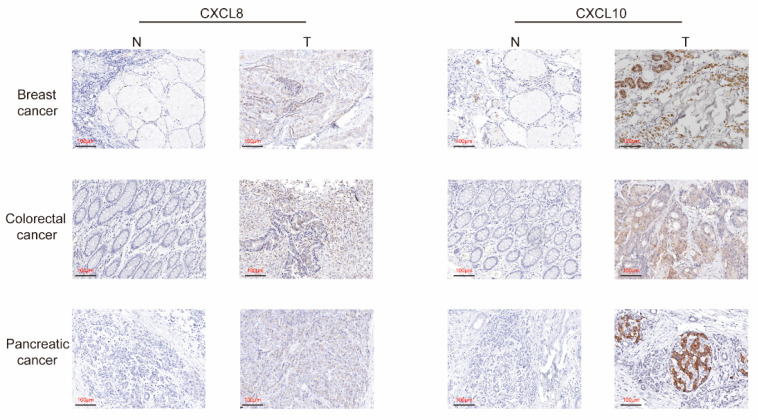
IHC analyses of CXCL8 and 10 in BRCA, COAD, and PDAC patients.

We examined CXCL protein levels through IHC and found that the protein expressions of CXCL8 and 10 were statistically significantly up-regulated in human breast cancer, pancreatic cancer, and colon cancer tissues versus the corresponding normal samples (Figure 6).

### 3.4. Prognostic Value of CXCs in Three Kinds of Cancer

We further made the survival analysis of CXCs in three cancers. A public dataset was used to analyze the associations between CXC mRNA levels and the survival time of breast cancer patients using the Kaplan–Meier mapping tool. The public dataset OncoLnc was used to analyze the associations between CXC mRNA levels and the survival of patients with colon and pancreatic cancer.

**Figure 7:**
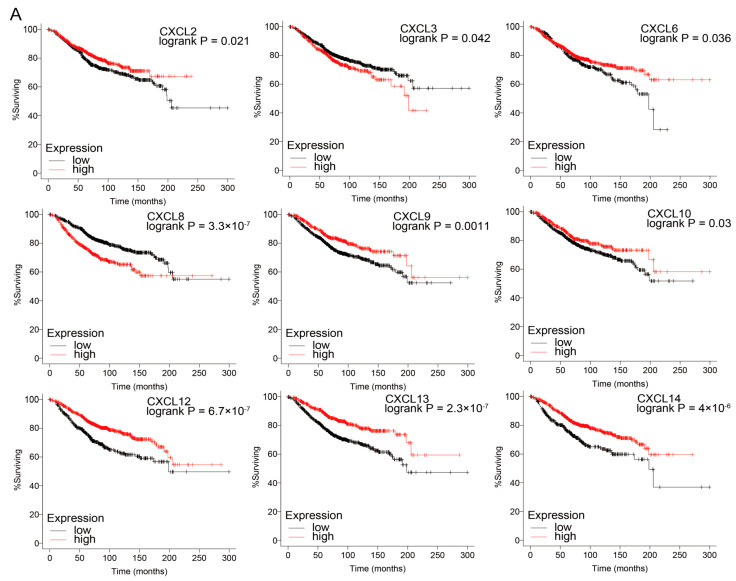

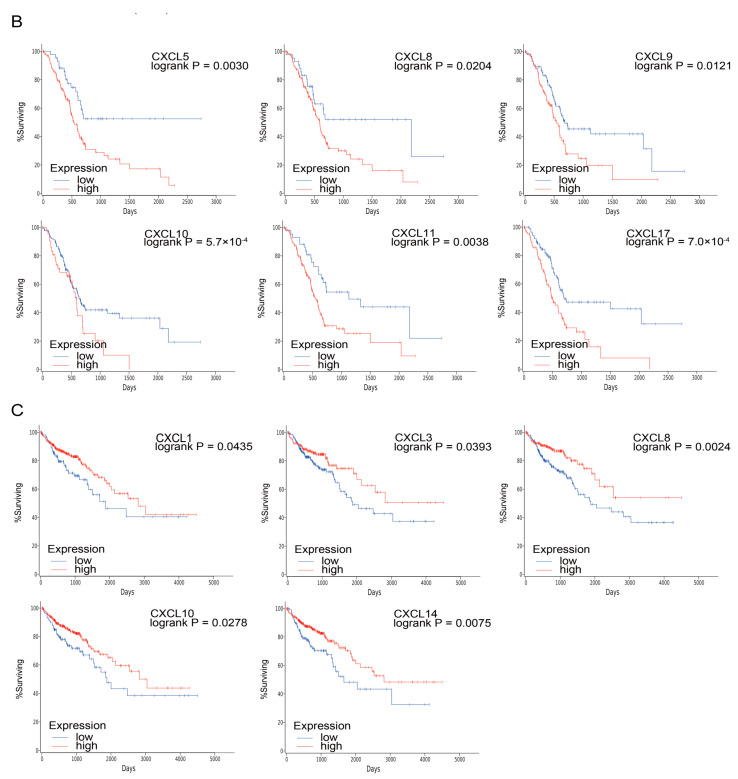
The prognostic value of mRNA level of CXCs in BRCA (Kaplan–Meier plotter) (**A**), COAD (**B**), and PDAC (**C**) patients (OncoLnc).

The Kaplan–Meier curves showed that in breast cancer patients, higher expression of mRNAs of CXCL2, 6, 9, 10, 12, 13, 14 and lower expression of mRNAs of CXCL3, 8, and 17 was significantly associated with longer overall survival (OS) (*p* < 0.05) (Figure 7A). In colon cancer tissues, higher mRNA expression of CXCL1, 3, 8, 10, and 14 was significantly associated with longer OS (*p* < 0.05) (Figure 7B). In patients with pancreatic cancer, lower mRNA expression of CXCL5, 8, 9, 10, 11, and 17 was significantly associated with longer OS (*p* < 0.05) (Figure 7C).

### 3.5. Prediction of Transcription Factors (TFs) Regulating CXCs

Because of the significant differences in the expression of CXCs in the cancer tissues versus normal tissues, we used the TRRUST database and KnockTF database to identify possible TFs and regulatory relationships between CXCs and TF.

**Figure 8:**
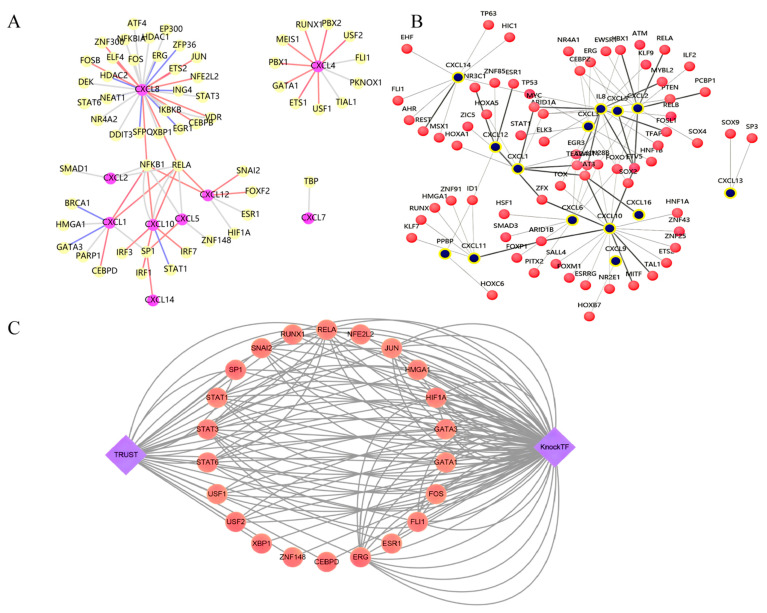
Prediction of TFs regulating CXCs. (**A**) TFs and regulatory relationships searched in the TRRUST database. The red line represents the activation regulatory relationship, the blue line represents the repression regulatory relationship, and the grey line represents the unknown regulatory relationship. (**B**) TFs and corresponding intergenes searched in the KnockTF database. The grey line represents ChIP-seq data supporting TF–target relationships. (**C**) Intersections of these two databases.

We determined that the key TFs of the CXC family include RELA, NFKB1, and SP1 (Table 2) (predicted). Additionally, we evaluated all TFs of the nine CXCs, including possible regulation modes (Figure 8A, Table 3) (experimentally validated). Interestingly, the same TF may induce different regulations in different studies, such as ELF4, NFKB1, and RELA, which are present in the lists of transcriptional activators and transcriptional suppressors of CXCL8. Meanwhile, we extracted a transcriptional regulatory subnetwork between CXCs and TFs using the KnockTF database. TF–target relationships supported by the ChIP-seq data were represented by the thick lines in the subnetwork. (Figure 8B, Table 4) TFs including RELA, NFE2L2, JUN, HMGA1, HIF1A, GATA3, GATA1, FOS, FLI1, ESR1, ERG, CEBPD, ZNF148, XBP1, USF1, STAT6, STAT3, SP1, SNAI2, and RUNX1 were involved in both databases (Figure 8C).

### 3.6. Regulation of CXCL8 and 10 by F. nucleatum in CAC

**Figure 9:**
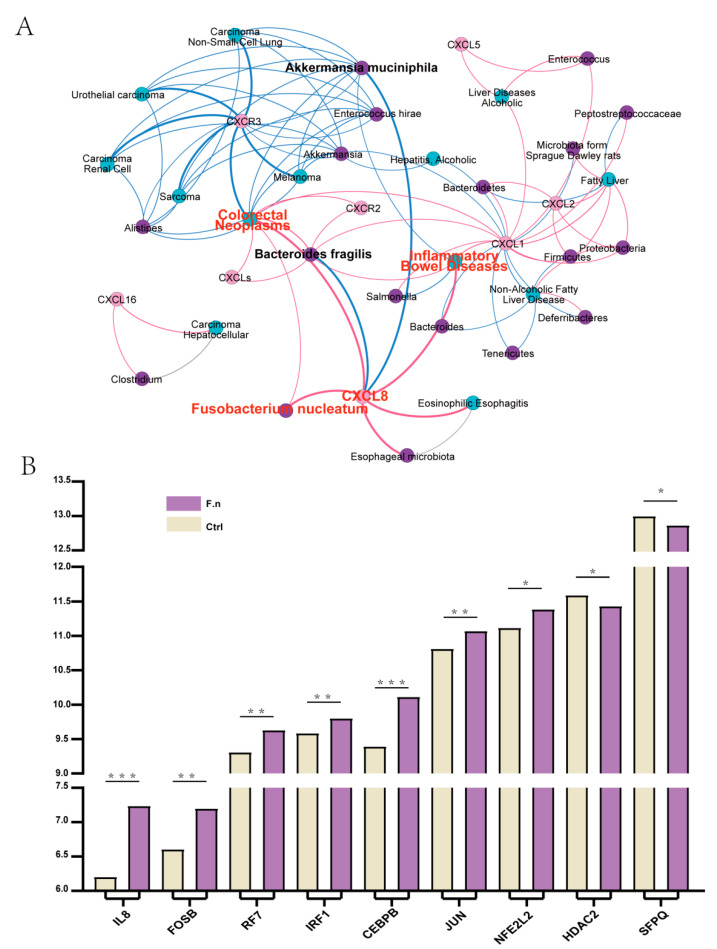
(**A**). Network analysis of CXCs, CXCRs, intestinal flora, and human diseases. (**B**). Expression of CXCL8 and TFs in GSE90944. (* *p* < 0.05, ** *p* < 0.01, *** *p* < 0.001).

The important contribution of the gut microbiota to human health and disease is widely recognized. Until now, more and more online databases have been developed to manage signatures of microbiota genomes, disease-related genes and proteins, as well as providing some analysis. Amadis is a database that provides microbiota–disease associations supported by experiments and interaction networks between them. By constructing an interaction network of CXCs, CXCRs, intestinal flora, and human diseases, we found that there could be a possible association between CXCL8, *Fusobacterium nucleatum*, and human diseases (including inflammatory bowel disease (IBD) and colon cancer) (Figure 9A).

**Figure 10:**
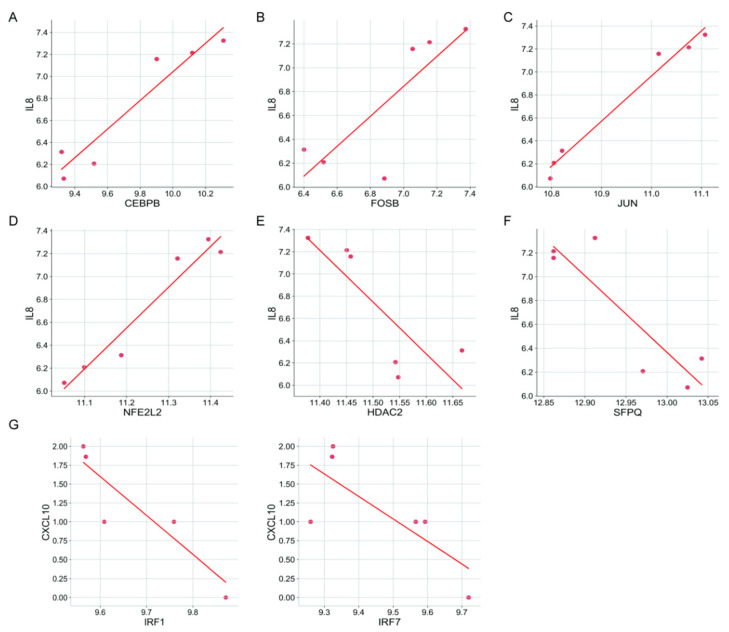
Correlation of CXCL8 and 10 with TFs. (**A**–**D**) Significant positive correlation of CXCL8 with CEBPB, FOSB, JUN, and NFE2L2. (**E**,**F**) Significant negative correlation of CXCL8 with HDAC2 and SFPQ. (**G**) Significant negative correlation of CXCL10 with IRF1 and IRF7.

Additionally, we analyzed the gene expression profile of the RNA-seq dataset (GSE90944) in HT-29 cell lines treated with or without *F. nucleatum*. Differential expression of CXCL8 and significant differential expression of TFs (predicted as shown in Table 3) could have activated CXCL8 transcription and activated CXCL10 transcription (Figure 9B). Correlation analysis showed that CXCL8 was significantly correlated with CEBPB, FOSB, JUN, NFE2L2, HDAC2, and SFPQ. CXCL10 was significantly correlated with IRF1 and IRF7 (Figure 10).

**Figure 11:**
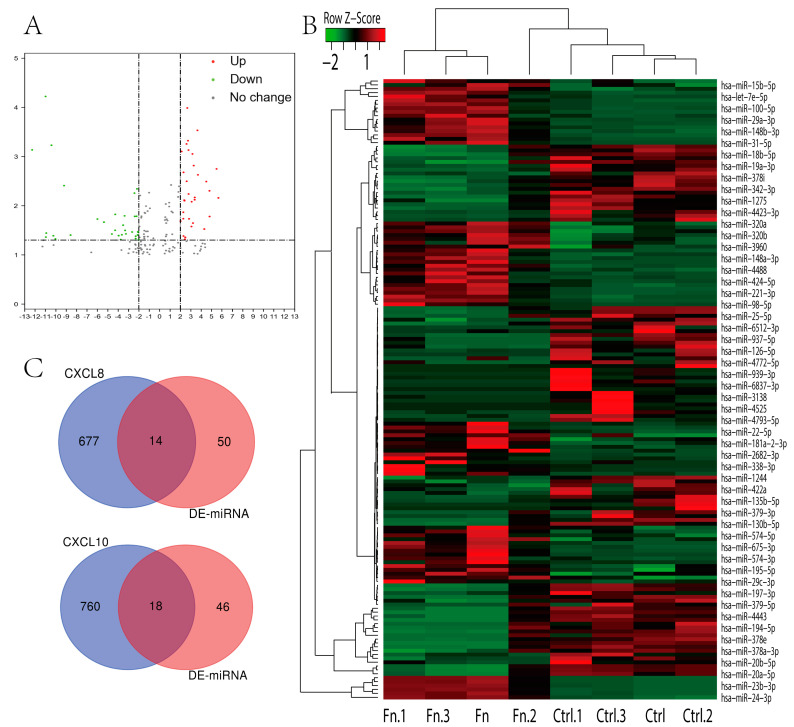
Expression of miRNA and possibly combined miRNA with CXCL8 and 10. (**A**,**B**) Volcano figure and heatmap of significantly differential miRNA. (**C**) Venn diagram of differential miRNA and possibly combined miRNA with CXCL8 and 10.

Subsequent analysis of the miRNA data presented in the Supplementary Materials identified a total of 64 differentially expressed miRNAs (*p* < 0.05, log2FC > 2) (Figure 11A,B). Analysis of the miRWalk database identified 804 miRNAs that can bind to the 3’-UTR of CXCL8 and 1015 miRNAs that can bind to the 3’-UTR of CXCL10 (Material S3) (predicted using bioinformatics tools). The intersection of differentially expressed miRNA with possibly bound miRNA identified seven downregulated miRNAs in the case of CXCL8 and 13 downregulated miRNAs in the case of CXCL10 (Figure 11C; Table 5).

**Figure 12:**
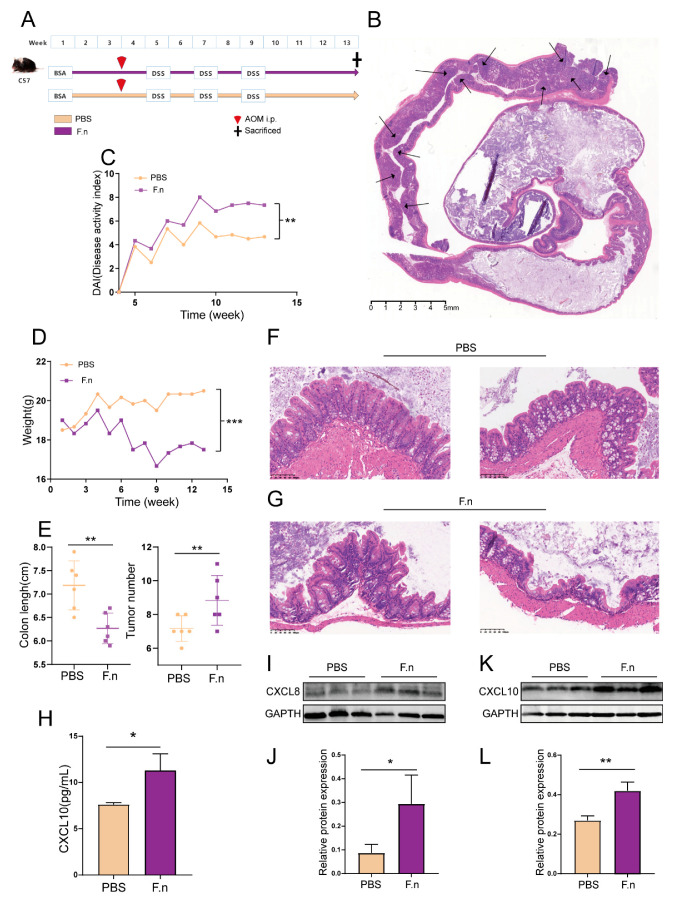
Treatment with *F. nucleatum* aggravates inflammation of the intestine, promotes tumorigenesis, and increases CXCL8 and 10 gene expression in AOM/DSS-induced CAC mice. (**A**) Timeline of the experimental schedule. (**B**) Representative images of H&E staining of the Swiss roll section of the colon segment. Changes in (**C**) disease activity index (DAI) and (**D**) body weight during a period of 13 weeks. (**E**) Colon length and tumor number of F.n-treated and PBS-treated mice. (**F**,**G**) Representative images of H&E staining of colon tissue (×200; scale bar, 100 μm). (**H**) Serum CXCL10 levels were evaluated by ELISA. (**I**–**L**) WB showed an increase in CXCL8 and 10 protein levels in the colon tissue of *F. nucleatum*-treated mice.Data are presented as means ± SD. * *p* < 0.05; ** *p* < 0.01; *** *p* < 0.001; Student’s *t*-test (two-tailed). The original Western blot images of (**I**&**K**) was shown in Material S8.

The result of the above analysis is in accordance with the in vivo experiment. By using the AOM/DSS-induced, colitis-associated cancer mouse model, we verified *F. nucleatum*’s regulatory role in the expression of CXCs (Figure 12A). Oral gavage with *F. nucleatum* aggravates the loss of body weight in CAC mice (Figure 12C,D). At the time of sacrifice, colons were removed, and colon length and tumor number were measured. Treatment with *F. nucleatum* significantly shortened the colon length and promoted tumorigenesis (Figure 12B,E). Inflammation of the intestine was histologically analyzed. Compared with the control group, treatment with *F. nucleatum* significantly increased the mucosal breaks of the oral administration group (Figure 12F,G). Blood was collected and assayed by ELISA. CXCL10 levels in the blood of mice with *F. nucleatum* gavage were significantly up-regulated (Figure 12H). WB analyses of colon tissue from CAC mice after their *F. nucleatum* administration revealed significant up-regulation of CXCL8 and 10 (Figure 12I–L). Generally, these results proved that treatment with *F. nucleatum* could aggravate inflammation of the intestine, promote tumorigenesis, and increase CXCL8 and 10 gene expression in AOM/DSS-induced CAC mice. These results suggest that in the presence of *F. nucleatum*, the expression of TFs and miRNAs is different and thus regulates the expression of CXCL8 and 10 to influence the occurrence and development of colon cancer.

### 3.7. Genetic Alteration and Co-Expression of CXCs in Three Types of Cancer

**Figure 13:**
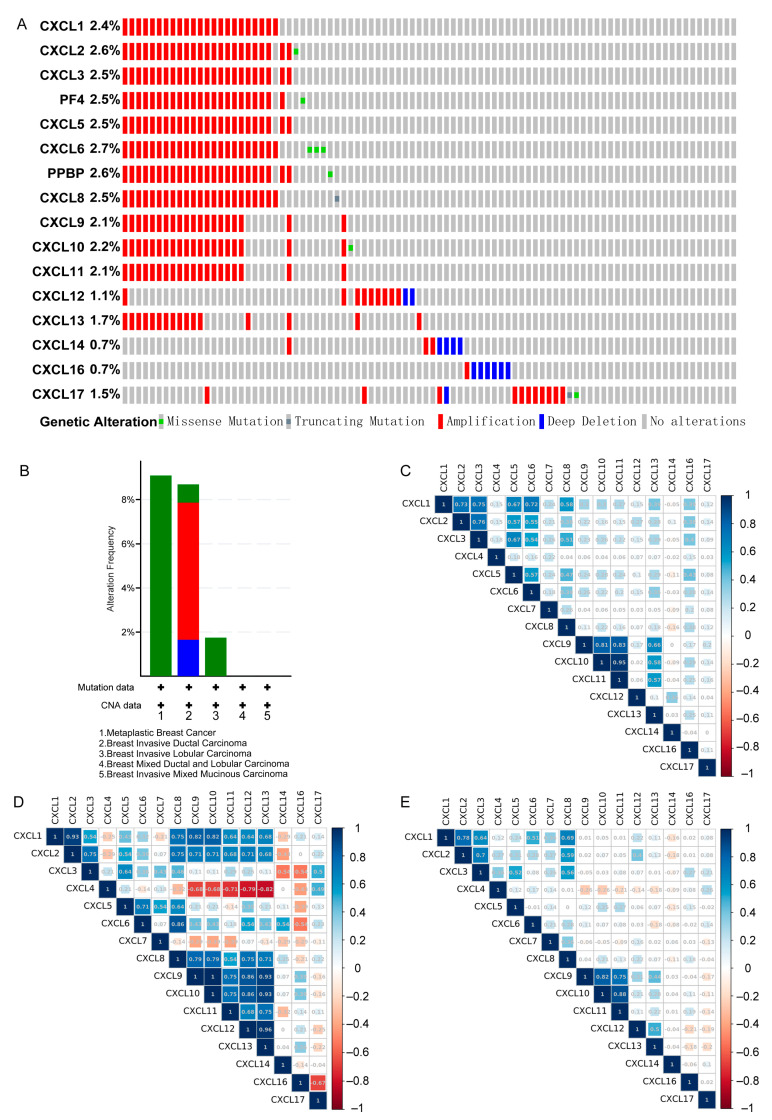
Genetic alteration and interaction analyses of different expressed CXCs (cBioPortal). (**A**,**B**) CXC gene expression and mutation analysis in breast cancer. (**C**) Correlation heat map of different expressed CXCs in BRCA. (**D**) Heat map of different expressed CXCs correlations in COAD. (**E**) Correlation heat map of different expressed CXCs in PDAC.

A comprehensive molecular characterization analysis of differentially expressed CXCs was performed. Genetic changes in CXCs were analyzed using the TCGA dataset in cBioPortal. The results indicated that 2.4%, 2.5%, 2.1%, 2.1%, 2.1%, 2.1%, 1.1%, 1.7%, and 0.7% genetic changes were present in CXCL1, 2, 3, 9, 10, 11, 12, 13, and 14 in the BRCA samples, respectively, and amplification was the most common type of the changes (Figure 13A,B). In the COAD samples, only 13 patients (6%) had genetic changes. In the PDAC samples, only six patients (4%) had genetic changes (Material S4).

We have explored the co-expression relationships of CXCs. In BRCA samples, there were significant positive correlations between the expression of CXCL1 and that of CXCL2, 3, 5, 6, and 8. These correlations were also found in the expression of CXCL2 and CXCL3, 5 and 6. and in the expression of CXCL3 and CXCL5, 6 and 8. It is also found that CXCL5, 6, and 9 were positively correlated with CXCL10, 11, and 13, and so is the expression of CXCL10 with the expression of CXCL11 and 13; similarly, the expression of CXCL11 was found to be positively correlated with the expression of CXCL13 (*p* < 0.05, R^2^ > 0.5).

In COAD samples, there are similarities in the correlations and also distinct differences. The expression of CXCL1 was found to be highly correlated with the expression CXCL2 and 9; however, CXCL4 was negatively correlated with CXCL12 and 13 (*p* < 0.05, R^2^ < −0.5). Positive correlations were also found between CXCL6 and CXCL8; CXCL8 and CXCL9, 10; and so were CXCL9 and CXCL10, 11, 12, and 13. It is also found that CXCL10 was positively correlated with CXCL12 and 13; and that CXCL12 was positively correlated with CXCL13 (*p* < 0.05, R^2^ > 0.5).

In PDAC samples, CXCL1 was highly correlated with CXCL2, 3, 6, and 8; CXCL2 was positively correlated with CXCL3 and 8. These correlations were also found in CXCL3 and CXCL5 and 8 and in CXCL9 and CXCL10 and 11. Similarly, the expression of CXCL10 was positively correlated with the expression of CXCL11 (*p* < 0.05, R^2^ > 0.5) (Figure 13C–E).

With the above co-expression analysis results, we found that the co-expression of CXCs may be related to the chromosomal localization of genes and transcription factors. CXCL1, 2, 3, 5, 6, 7, and 8 are located at 4q12-13. In BRCA samples, the expression of CXCL1 was highly correlated with CXCL2, 3, 5, 6, and 8. In PDAC samples, the expression of CXCL1 was positively correlated with CXCL2, 3, 6, and 8. Meanwhile, according to the analysis of transcription factors, the co-expressed genes may be regulated by the same one or more transcription factors, as NFκB and RELA may be responsible for multiple CXCs, including CXCL1, 2, 5, 8, 10, and 12.

### 3.8. Prediction of CXC-Interacting Proteins and Their Functions and Pathways

**Figure 14:**
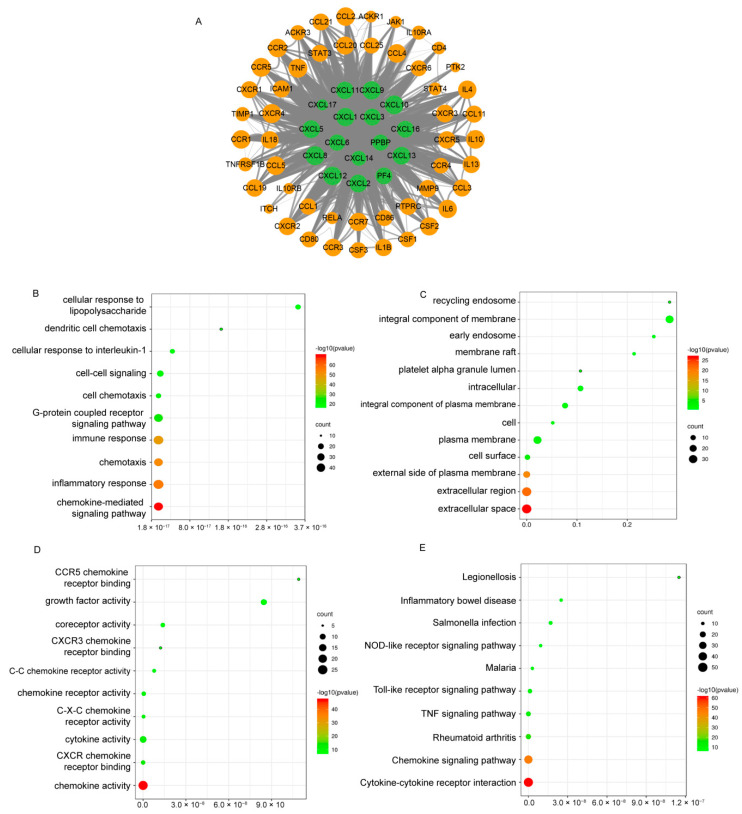
Predicted functions and pathways of CXCs and their 50 frequently interacting proteins in BRCA, COAD, and PDAC patients (String and DAVID). (**A**) Protein–protein interaction network of differentially expressed CXCs and 50 interacting proteins. (**B**–**D**) GO functional enrichment analysis predicted three main functions of CXCs and their 50 interacting proteins, namely, the biological process, cellular components, and molecular functions. (**E**) KEGG pathway analysis on CXCs and their 50 interacting proteins.

The CXC family performed functions by binding to receptors, so it is important to analyze the relation between CXCs and proteins interacting with CXCs. We analyzed 50 proteins interacting with CXCs using the String database. As a result, 66 nodes and 1498 edges were obtained in the PPI network, and a network map was constructed using Cytoscape (Figure 14A).

Additionally, the functions of CXCs and their 50 interacting proteins were analyzed using the DAVID database by GO and KEGG enrichment analysis. The results presented the top 10 highly enriched biological processes pathways include chemokine-mediated signaling pathways, inflammatory responses, chemotaxis, immune responses, G protein-coupled receptor signaling pathways, cell chemotaxis, and other biological processes, suggesting that CXCs in cancer are involved in chemotaxis and function in the inflammatory response (Figure 14B). The extracellular space, extracellular region, outer plasma membrane, cell surface, plasma membrane, and cell area were the main enrichment terms of CXCs (Figure 14C). In the molecular function categories, CXCs and CXCs-interacting proteins were enriched in chemokine activity and CXCR-chemokine-receptor-binding activity (Figure 14D).

It is known that CXCL1, 2, 3, 5, 6, 7, and 8 are bound to CXCR1 and 2; CXCL9, 10, and 11 are bound to CXCR3. Correlation analysis was performed for CXCs sharing the same receptor and their targets. All correlation coefficients between CXCR and CXCs were not significant (R < 0.8), suggesting that there was no strong correlation between CXCs and CXCR expressed in colon cancer (Material S5).

In KEGG analysis, the main enriched signal pathways were as follows: hsa04060: cytokine–cytokine receptor interaction; hsa04062: chemokine signal pathway; hsa05323: rheumatoid arthritis; hsa04668: tumor necrosis factor signal pathway; hsa04620: Toll-like receptor signal pathway; hsa05144: malaria; hsa04621: node-like receptor signal pathway; hsa05132: *Salmonella* infection; hsa05321: inflammatory bowel disease (IBD); and hsa05134: Legionnaires’ disease (Figure 14E). Most of these pathways are tightly related to inflammation and the development of cancer.

**Figure 15:**
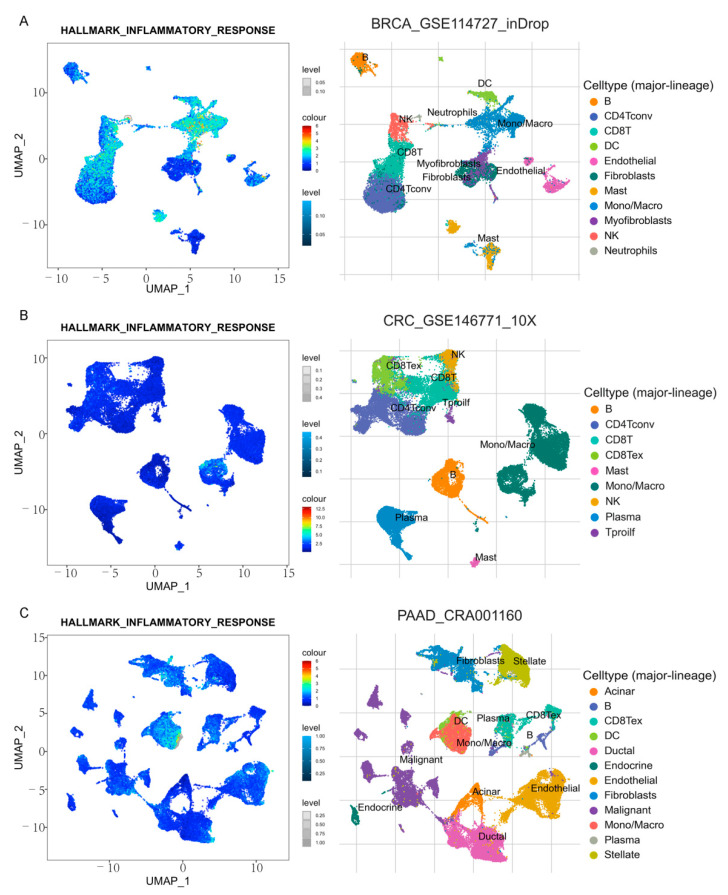
Computing and visualizing GSEA pathway enrichment score of inflammatory response (TISCH). Enrichment analysis in BRCA (**A**), COAD (**B**), and PDAC(**C**) patients.

The TISCH database was used to analyze and visualize the enrichment scores of inflammatory response signaling pathways in each cell subgroup. Inflammatory response signaling pathways were found to be enriched in mononuclear/macrophage subsets in all three cancer datasets (Figure 15).

### 3.9. Immune Cell Infiltration and CXCs in Three Types of Cancer

At present, the function of CXCs is still controversial. Some studies have found that tumor cells secrete CXCs to act on their own surface receptors [28], while other studies have revealed that CXCs can act as a signal to recruit immune cells [29]. The results of the functional enrichment and pathway analyses suggest that CXCs may influence the clinical outcome of cancer patients through regulating inflammatory response and immune cell infiltration. Therefore, we used the TIMER database to explore specific features of CXCs.

**Figure 16:**
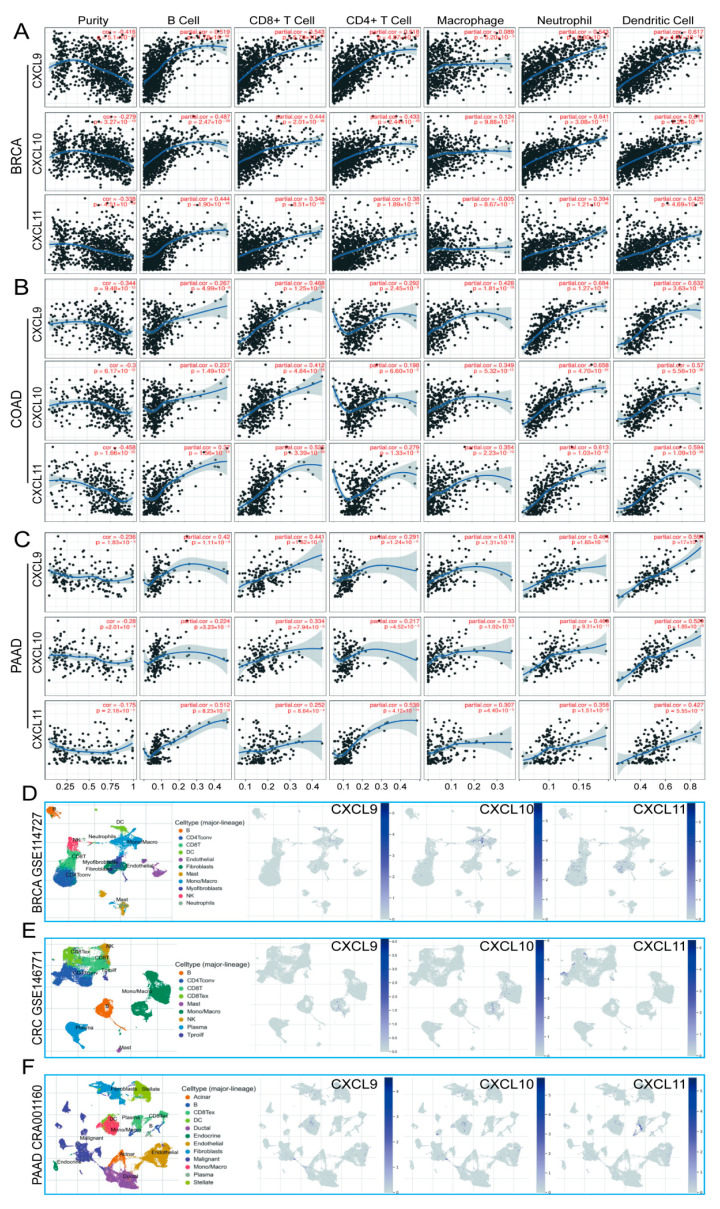
Correlation and distribution of differentially expressed CXCs and immune cell infiltration in BRCA, COAD, and PDAC (TIMER and TISCH). The association between the expression of CXCL9, 10, and 13 and the abundance of tumor-infiltrating immune cells in BRCA (**A**,**D**), COAD (**B**,**E**), and PDAC (**C**,**F**).

We analyzed the correlations between each CXC and tumor purity, B cells, CD8 + T cells, CD4 + T cells, macrophages, neutrophils, and dendritic cells in three types of cancer. A total of 244 pairs with significant correlation were detected, including 24 pairs with a partial correlation coefficient (Partial.cor) > 0.5; all pairs were positively correlated. As shown, for these 24 pairs of data, we mainly focused on the association between CXCL9, 10, and 13 and infiltrating immune cells (Figure 16A,B,C). Other related data are shown in Material S6.

We used the TISCH database to analyze the distribution of CXCL9, 10, and 13 cells in each subgroup of three types of cancer. It was found that CXCL9 and 10 were essentially enriched in mononuclear/macrophage subsets among the three cancers. CXCL13 is enriched in fibroblasts and CD8 + T cells in breast cancer, CD8 + Tex and CD4 + Tconv cells in colon cancer, and plasma in pancreatic cancer (Figure 16D,E,F).

We also analyzed the distribution of CXCR in colon cancer. The results showed that CXCR1 mainly expresses in NK cells. CXCR2 mainly expresses in neutrophils and monocytes/macrophages. CXCR3 is widely distributed in Treg, Tprolif, CD8T, CD8Tex, and CD4Tconv cells. CXCR4 is widely distributed in T cells, such as Treg, Tprolif, and CD8T, as well as in NK cells and B cells. CXCR5 is mainly distributed in B cells. CXCR6 is mainly distributed in NK cells and T cells, such as Treg, Tprolif, and CD8T (Material S7). These results may indicate that CXCs play a role in recruiting immune cells by binding to receptors on the surface of immune cells.

## 4. Discussion

The imbalance of CXC expression has a considerable impact on tumorigenesis, tumoral cell proliferation, apoptosis, and tumor metastasis. Intercellular communications between stromal cells and tumor cells affect the expression of CXCs in various types of cells, thus regulating tumor metastasis and invasion. Some studies have already shown correlations between CXCs and the tumor microenvironment, suggesting that CXCs can regulate tumor progression and immunotherapy. Our previous studies have shown that the protection of colorectal cancer cells from radiotherapy by CXCL12/CXCR4 is mediated by survivin in colorectal cancer [12]. CXCL10 is considered a potential therapeutic target for melanoma [30]. The application of CXCL8 for the diagnosis of CRC is more practical than the use of the classical tumor marker CEA. Serum CXCL8 may be a potential biomarker of colorectal cancer progression [31]. Some studies have demonstrated unique weak binding between CXCL8 and CXCR2 and interaction between CXCR2 and G proteins [32]. However, there is a lack of a bioinformatics analysis that demonstrates the prognostic values and biological functions of CXCs in multiple tumors. In this study, we demonstrated abnormal expression of CXCs in 20 types of cancer and significant differences in the mRNA expression of the CXC family members that have significant prognostic value in breast cancer, colon -cancer, and pancreatic cancer. This study is the first to suggest that the CXCL family may be involved in the interactions between intestinal flora and colonic epithelium of the host. We hope that our findings will help to improve our understanding of the roles of the CXCL family members and improve treatment design and the accuracy of prognosis in patients with these tumors.

We initially investigated the expression of CXC chemokines and their relationships with pathological stages of the tumors. We found that nine genes were differentially expressed in breast cancer versus normal tissues (CXCL9, 10, 11, and 13 were up-regulated, and CXCL1, 2, 3, 12, and 14 were downregulated). Additionally, we demonstrated that the expression of CXCL1, 2, 5, 8, 12, 13, and 14 was closely associated with the stage of breast cancer. Similarly, 11 genes were differentially expressed in colon cancer (CXCL1, 2, 3, 5, 8, 9, 10, and 11 were up-regulated, and CXCL11, 12, 13, and 14 were downregulated). The development of tumors was associated with an increase in the expression of CXCL9, 10, and 11. The results on pancreatic cancer data showed that 12 genes were up-regulated (CXCL1, 2, 3, 5, 6, 8, 9, 10, 13, 14, 16, and 17). The expression of CXCL1, 3, 5, and 8 were associated with the stages of pancreatic cancer. These data suggest that differentially expressed CXC chemokines may play important roles in these three types of tumors.

Analysis using large groups of patients with breast, colon, or pancreatic cancer in the K–M plotter database indicated that a number of CXC family members were significantly associated with survival and had specific associations. In patients with colon cancer, the survival time of patients with higher levels of expression of CXCL1, 3, 8, 10, and 14 was longer than that of patients with lower expression. In pancreatic cancer patients, the survival time of patients with higher levels of expression of CXCL5, 8, 9, 10, 11, and 17 was remarkably shortened. In breast cancer, the groups with higher expression of mRNAs of CXCL2, 6, 9, 10, 12, 13, and 14 and the groups with lower expression of CXCL3, 8, and 17 had significantly better overall survival (OS).

There are contradictory evidences in the role of CXCL8 in the development and progression of colon cancer. High amounts of serum CXCL8 prevent liver metastasis of CRC and are correlated with good favorable prognostic outcomes [33]. In contrast, elevated CXCL8 levels promote carcinogenesis and are associated with poor prognosis [34]. The analysis showed that the expression of CXCL8 in colon cancer was higher than in normal controls, and patients with high expression of CXCL8 in colon cancer had a longer survival time. In breast cancer and pancreatic cancer patients, the expression of CXCL8 was higher in the tumor tissues; however, the OS time of patients who had a higher expression of CXCL8 was significantly shorter. This contradictory phenomenon reflects the complex role of CXCL8 in the occurrence and development of colon cancer. The intestinal microflora is closely linked to colonic disease. Colonic tissue directly interacts with intestinal flora, and multiple studies noted that intestinal microorganisms play a significant role in the development and progression of colon cancer [35], inflammation-related colon cancer [36], the colon cancer microenvironment [37], and colon cancer drug resistance [38]. Network analyses were carried out using the Amadis database analysis tool, and a possible association between CXCL8, *Fusobacterium nucleatum*, and human diseases (including inflammatory bowel disease and colon cancer) was found. We investigated the results of sequencing obtained after coculture of *Fusobacterium nucleatum* with colon cancer cell lines and determined that the expression of CXCL8 was significantly increased, and the expression of CXCL10 was decreased in the HT29 cell line cocultured with *Fusobacterium nucleatum*. *Fusobacterium nucleatum* may change tumor proliferation, invasion, metastasis, and drug resistance by increasing the expression of CXCL8 and reducing the expression of CXCL10, thus affecting the prognosis of patients. Combining these data with the data on differential expression of miRNAs in SW480 cells cocultured with *Fusobacterium nucleatum* indicated changes in the expression levels of transcription factors related to CXCL8 and 10, transcriptional suppressors, and miRNAs acting on the corresponding 3’-UTR of mRNAs. At present, there are no reports on the influence of the intestinal flora on the prognosis of colon cancer patients and the progression of colon cancer mediated by the expression of chemokines. Our analysis demonstrated that *Fusobacterium nucleatum* might influence the changes in the chemokine family members at the transcriptional and posttranscriptional levels and, thus, influence the development and progression of colon cancer and the prognosis. Although the expression of CXCL8 and 10 increased significantly, their role in the process of *F. nucleatum* aggravating CAC remains unclear. Future experimental verification of the mechanism may identify new pathogenic pathways and therapeutic targets.

Co-expression analysis revealed that the co-expression of CXCs might be related to their chromosomal locations, and this co-expression might be regulated by transcription factors. Interestingly, in the analysis of the expression of CXCRs, we found that there was no significant difference in the expression of CXCRs in tumor tissue and normal tissue, and there was no diagnostic or prognostic value of CXCRs. Through the single-cell sequencing database data, we found that CXCRs are mostly distributed on the surface of immune cells, which may indicate that CXCs play a role in recruiting immune cells by binding to receptors on the surface of immune cells.

Our study has certain limitations. The results at the transcriptional level can reflect the immune status; however, this analysis cannot reflect the overall changes. Independent cohort studies should be performed to verify our results. The CXC family members may play a dual role in disease progression. Increased expression of CXCs in tumor tissues may promote carcinogenesis and regulate the tumor microenvironment; however, in some tumors, high expression of the CXC family members may suggest a better overall survival time. Most of the results were predicted by bioinformatics analysis, and, as a result, further experiments in vitro or in vivo are needed to demonstrate the associations between these factors.

## 5. Conclusions

In this study, we systematically analyzed the expression and prognostic value of CXCs in a variety of tumors and provided a thorough evaluation of the heterogeneity and complexity of the molecular and biological characteristics of the tumors. High expression of certain CXCs can be used as a molecular marker to identify tumor patients in high-risk groups. This is the first study to propose the theory that intestinal flora may influence disease by influencing the transcriptional changes in CXCs, thus providing a direction for further research. Our results indicate that CXCs are a potential therapeutic target in a variety of tumors and a potential prognostic marker to improve the survival of cancer patients and accuracy of prognosis, and they may be involved in diseases caused by intestinal flora.

## Figures and Tables

**Table 1:** Differential expression of CXCs in datasets of Oncomine.

CXCs	Type	Fold Change	*p* Value	*t*-Test	Dataset
CXCL1	Invasive ductal breast carcinoma	−6.050	9.30 × 10^−30^	−14.245	TCGA
	Breast cancer	−3.805	9.82 × 10^−75^	−20.522	Bittner Multi-cancer Statistics
	Colorectal cancer	6.308	7.49 × 10^−121^	29.225	Bittner Multi-cancer Statistics
	Colon adenocarcinoma	6.365	4.39 × 10^−12^	10.236	TCGA
CXCL2	Invasive ductal breast carcinoma	−36.063	4.18 × 10^−59^	−32.023	TCGA
	Invasive ductal breast carcinoma	−3.671	1.28 × 10^−51^	−23.342	Curtis Breast Statistics
	Colorectal cancer	3.055	4.69 × 10^−71^	20.072	Bittner Multi-cancer Statistics
	Pancreatic cancer	3.904	2.20 × 10^−7^	5.798	Barretina CellLine Statistics
CXCL3	Breast cancer	−3.484	1.51 × 10^−61^	−18.125	Bittner Multi-cancer Statistics
	Invasive ductal breast carcinoma	−7.462	4.22 × 10^−34^	−19.062	TCGA
	Colorectal cancer	9.737	7.64 × 10^−152^	36.102	Bittner Multi-cancer Statistics
	Colon adenocarcinoma	8.551	2.61 × 10^−13^	11.423	TCGA
	Pancreatic carcinoma	5.062	3.80 × 10^−7^	5.806	Pei Pancreas Statistics
	Pancreatic ductal adenocarcinoma	3.907	1.19 × 10^−8^	6.423	Badea Pancreas Statistics
	Pancreatic cancer	2.088	4.70 × 10^−4^	3.536	Barretina CellLine Statistics
CXCL4	Colorectal cancer	2.314	7.21 × 10^−40^	14.283	Bittner Multi-cancer Statistics
CXCL5	Colon adenocarcinoma	2.739	3.6 × 10^−22^	11.937	TCGA
	Colorectal cancer	4.493	1.43 × 10^−42^	15.077	Bittner Multi-cancer Statistics
	Pancreatic carcinoma	12.881	5.58 × 10^−9^	6.828	Pei Pancreas Statistics
	Pancreatic cancer	7.439	1.22 × 10^−6^	5.395	Barretina CellLine Statistics
CXCL6	Breast cancer	−3.256	1.33 × 10^−54^	−16.970	Bittner Multi-cancer Statistics
	Colon adenocarcinoma	2.859	5.44 × 10^−12^	8.66	TCGA
CXCL7	Breast cancer	−2.707	3.97 × 10^−33^	−12.629	Bittner Multi-cancer Statistics
	Colorectal cancer	3.02	3.35 × 10^−27^	11.438	Bittner Multi-cancer Statistics
CXCL8	Breast cancer	−4.229	3.32 × 10^−60^	−17.871	Bittner Multi-cancer Statistics
	Invasive ductal breast carcinoma	−2.746	2.20 × 10^−2^	−2.338	Curtis Breast Statistics
	Colorectal cancer	7.457	2.12 × 10^−85^	23.265	Bittner Multi-cancer Statistics
	Pancreatic ductal adenocarcinoma	9.800	9.92 × 10^−12^	7.971	Badea Pancreas Statistics
	Pancreatic carcinoma	8.378	3.51 × 10^−6^	5.473	Pei Pancreas Statistics
CXCL9	Invasive ductal breast carcinoma	4.663	7.27 × 10^−91^	29.060	Curtis Breast Statistics
CXCL10	Invasive ductal breast carcinoma	5.022	4.40 × 10^−71^	26.711	Curtis Breast Statistics
	Pancreatic carcinoma	3.950	1.41 × 10^−4^	4.190	Pei Pancreas Statistics
CXCL11	Colon adenocarcinoma	2.304	7.74 × 10^−5^	3.885	TCGA
CXCL12	Invasive ductal breast carcinoma	−5.734	2.59 × 10^−110^	−41.690	Curtis Breast Statistics
	Invasive ductal breast carcinoma	−6.730	3.41 × 10^−51^	−25.411	TCGA
	Colon adenocarcinoma	−10.511	5.53 × 10^−34^	−22.305	TCGA
CXCL13	Ductal breast carcinoma	2.238	5.72 × 10^−4^	3.358	Bittner Breast Statistics
CXCL14	Invasive ductal breast carcinoma	−2.991	4.38 × 10^−55^	−20.207	Curtis Breast Statistics
	Invasive ductal breast carcinoma	−3.074	5.92 × 10^−34^	−14.493	TCGA
CXCL16	Pancreatic ductal adenocarcinoma	2.311	6.64 × 10^−12^	8.384	Badea Pancreas Statistics
	Pancreatic cancer	2.395	2.11 × 10^−6^	5.172	Barretina CellLine Statistics
CXCL17	Colorectal cancer	−2.896	4.16 × 10^−41^	−13.988	Bittner Multi-cancer Statistics

**Table 2:** Key transcription factor of CXCs.

Key TF	Description	*p* Value	Overlapped Genes
RELA	A subunit of NF-kappa B that is primarily responsible for its transactivation function. It contains a C-terminal transactivation domain and an N-terminal domain with homology to PROTO-ONCOGENE PROTEINS C-REL.	1.09 × 10^−7^	CXCL10, CXCL8, CXCL12, CXCL2, CXCL5, CXCL1
NFKB1	Nuclear factor of kappa light polypeptide gene enhancer in B-cells 1 protein, human.	1.14 × 10^−7^	CXCL12, CXCL8, CXCL10, CXCL5, CXCL1, CXCL2
SP1	Promoter-specific RNA polymerase II transcription factor that binds to the GC box, one of the upstream promoter elements, in mammalian cells.	0.00683	CXCL5, CXCL1, CXCL14

**Table 3:** TFs and regulatory relationships searched in TRRUST database.

CXCs	Type	TF	Ref (PMID)
CXCL1	Activation	CEBPD, NFKB1, RELA	23028973; 10530453; 15958549
	Repression	BRCA1, GATA3	22120723
	Unknown	HMGA1, NFKB1, PARP1, RELA, SP1	11112786; 16040075; 7479086
CXCL2	Unknown	NFKB1, RELA, SMAD1	17363596
PF4	Activation	ETS1, GATA1, MEIS1, PBX1, PBX2, RUNX1, USF1, USF2	12609849; 12732210; 21129147; 15187018
	Repression	RUNX1	17150917
	Unknown	ETS1, FLI1, PKNOX1, RUNX1, TIAL1	23848403; 12732210; 9207209
CXCL5	Activation	SP1	11559712
	Unknown	NFKB1, RELA, ZNF148	11559712
PPBP	Unknown	TBP	7958954
CXCL8	Activation	CEBPB, ELF4, ETS2, FOSB, JUN, NFE2L2, NFKB1, RELA, VDR, ZNF300	15085176; 15688424; 14625302; 12438253; 20826776; 16701870; 18684922; 21925595; 11512674; 16764699; 17040605; 17041011; 17917246; 18074095; 18996370; 22340043; 10506755; 10530453; 11297551; 11564889; 12058956; 15350531; 15387324; 15958549; 22340043; 22891766; 7876168; 9431991; 21777376
	Repression	EGR1, ELF4, ERG, HDAC2, ING4, NEAT1, NFKB1, RELA, SFPQ, ZFP36	12438253; 19359602; 22235125; 19411311; 17848618; 20707719; 15517885; 8413215; 24507715; 21593445
	Unknown	ATF4, CEBPB, DDIT3, DEK, EGR1, EP300, FOS, HDAC1, HDAC2, IKBKB, JUN, NFKB1, NFKBIA, NR4A2, RELA, STAT3, STAT6, VDR, XBP1	16931790; 11120852; 18772138; 16829531; 19837667; 19966855; 12218154; 11564889; 12296854; 12643600; 12716652; 14670967; 10477716; 11342414; 11953364; 12485925; 12707271; 14631383; 14670967; 15289496; 15950427; 16004996; 16105834; 16803583; 17045242; 18281166; 19376732; 19732956; 19786024; 8878392; 9698090; 8617886; 17726017; 24250750
CXCL10	Activation	IRF1, IRF3, IRF7, NFKB1, RELA	19342664; 18691624; 24257594; 16982926; 20164184; 24701034
	Repression	STAT1	22022583
	Unknown	NFKB1, RELA, STAT1	16818736; 19479051; 24257594; 23153456
CXCL12	Activation	FOXF2, NFKB1, RELA, SNAI2	19562724; 17530707; 22074556
	Unknown	ESR1, HIF1A	18563714; 12384916
CXCL14	Activation	SP1	22382027

**Table 4:** TFs and corresponding intergenes searched in KnockTF database.

TF	Intergenes	Numbers	*p* Value	FDR
AHR	CXCL16; CXCL11; CXCL14; CXCL9; CXCL6; CXCL3; CXCL5	7	1.13 × 10^−6^	0.000347
ARID1A	IL8; CXCL1; CXCL5; CXCL12; CXCL2; CXCL6	6	0.00161	0.0216
CREB1	CXCL13; CXCL1; CXCL12; CXCL9; PF4; CXCL5; CXCL17; CXCL14; CXCL11; CXCL6; CXCL10; CXCL16; CXCL2	13	0.0039	0.0428
EGR3	CXCL1; CXCL6; CXCL3; CXCL5; CXCL13; CXCL10; CXCL2; CXCL11; CXCL9; CXCL16	10	1.59 × 10^−5^	0.000976
ERG	CXCL3; CXCL5; CXCL2; CXCL1; CXCL6; PPBP; PF4; CXCL10; CXCL12; CXCL9; CXCL16; CXCL17; CXCL14; CXCL11	14	3.21 × 10^−5^	0.00164
ESRRG	CXCL5; CXCL1; IL8; CXCL6; CXCL10	5	0.000465	0.00892
ETV5	CXCL10; IL8; CXCL5; CXCL2; CXCL1; CXCL6	6	0.000321	0.00704
FOSL1	IL8; CXCL6; CXCL10; CXCL5	4	0.000703	0.0121
FOXO1	CXCL14; CXCL12; CXCL6; CXCL3; IL8; CXCL2; CXCL10; CXCL1; CXCL5	9	7.42 × 10^−5^	0.00228
FOXP1	PPBP; CXCL3; PF4; CXCL5; CXCL13; CXCL6; IL8; CXCL1; CXCL2; CXCL11; CXCL17; CXCL14; CXCL10; CXCL9; CXCL16; CXCL12	16	8.60 × 10^−6^	0.000976
HNF1A	CXCL12; CXCL10; CXCL11; CXCL9; CXCL3; CXCL1; CXCL2; CXCL5; PF4; IL8; CXCL17; CXCL14; CXCL6; CXCL16	14	1.31 × 10^−5^	0.000976
HNF1B	CXCL3; CXCL1; CXCL5; CXCL16; PPBP; CXCL14	6	0.00329	0.0404
HOXA5	CXCL1; IL8; CXCL2; CXCL16	4	1.23 × 10^−5^	0.000976
HOXD9	CXCL6; CXCL16; CXCL5; CXCL10; PPBP; CXCL17; CXCL1; PF4; CXCL13; CXCL3; CXCL2; CXCL11	12	0.000157	0.00402
KLF4	CXCL5; CXCL17; CXCL2; CXCL14; CXCL10; PPBP	6	0.000429	0.00878
LIN28B	CXCL1; CXCL6; CXCL10; CXCL2; CXCL3	5	0.00162	0.0216
MYBL2	IL8; PPBP	2	0.00405	0.0429
NR2F2	CXCL13; CXCL9; CXCL14; PF4; CXCL3; CXCL10; CXCL11; CXCL6; CXCL1; PPBP; CXCL5; CXCL2; CXCL16	13	0.00358	0.0423
PITX2	CXCL10; CXCL6; CXCL1	3	0.00111	0.017
POLR3A	PPBP; CXCL14; CXCL5; CXCL3; CXCL13; CXCL9; CXCL11; CXCL10; CXCL6	9	0.00378	0.0428
Prox1	CXCL3; CXCL13; CXCL11; CXCL5; CXCL2; CXCL16; CXCL1; CXCL17; PPBP; CXCL6	10	0.00122	0.0178
PTEN	CXCL14; CXCL13; CXCL16; PF4; PPBP; CXCL9; CXCL17; CXCL12; CXCL10; IL8; CXCL1; CXCL3; CXCL11; CXCL2; CXCL5	15	6.24 × 10^−5^	0.00228
RARA	PPBP; CXCL9; CXCL13; CXCL17; CXCL11; CXCL3; CXCL1; CXCL2; CXCL14	9	0.00021	0.00496
SOX17	PPBP; IL8; CXCL6; CXCL10; CXCL1; CXCL11; PF4; CXCL3; CXCL2; CXCL12; CXCL5	11	8.18 × 10^−5^	0.00228
SOX4	CXCL2; CXCL3; CXCL14; CXCL1; CXCL13; CXCL16; IL8; CXCL10	8	0.00101	0.0163
SOX9	CXCL13; CXCL5; CXCL6; CXCL3; PF4; CXCL17; PPBP; CXCL1; CXCL10; CXCL2	10	5.16 × 10^−5^	0.00226
SPDEF	CXCL2; PF4; CXCL14; CXCL13; PPBP; CXCL11; CXCL6; CXCL1; CXCL9	9	0.00071	0.0121
YY1	CXCL11; CXCL6; CXCL12; PF4; CXCL9; CXCL17; CXCL13; CXCL5; CXCL16; CXCL3	10	6.72 × 10^−5^	0.00228
ZNF25	CXCL10; CXCL5; CXCL6	3	0.0029	0.0371

**Table 5:** Downregulated miRNAs binding with CXCL8 and up-regulated miRNAs binding with CXCL10.

Binding Gene	Mirnaid	Binding *p* Value	Accessibility	Number of Pairings	Binding Region Length	Longest Consecutive Pairings
CXCL8	hsa-miR-19a-3p	0.923077	0.016853	17	22	10
	hsa-miR-3138	0.846154	0.009999	19	23	9
	hsa-miR-4306	0.846154	0.019455	13	16	7
	hsa-miR-4793-3p	1	0.006677	17	24	9
	hsa-miR-6793-5p	1	2.62 × 10^−5^	19	29	12
	hsa-miR-6837-5p	1	0.000791	17	20	12
	hsa-miR-6837-3p	0.846154	0.000961	18	26	8
CXCL10	hsa-let-7b-5p	0.923077	0.000462	18	21	10
	hsa-miR-23a-3p	0.846154	0.079181	12	18	8
	hsa-miR-196a-5p	0.923077	0.000483	17	19	17
	hsa-miR-34a-5p	0.846154	0.000568	15	19	6
	hsa-let-7i-5p	0.923077	0.000462	17	22	8
	hsa-miR-23b-3p	0.846154	0.079181	12	18	8

## Data Availability

Publicly available datasets were analyzed in this study. Data source and accessed date was described in materials and methods.

## Supplementary Materials

The following are available online at https://www.mdpi.com/article/10.3390/cancers13164153/s1, Material S1: Subpopulation distribution of CXCs in single-cell sequencing datasets of the three cancers. Material S2: Expression differences of CXCRs in three cancers. Material S3: MiRNAs predicted binding to CXCL8 and 10 by miRWalk database. Material S4: CXC gene expression and mutation analysis in pancreatic cancer and colon cancer. Material S5: Correlation analysis of CXCRs and CXCs in colon cancer. Material S6: Correlation of other differentially expressed CXCs and immune cell infiltration in BRCA, COAD, and PDAC. Material S7: Distribution of CXCRs in colon cancer; Material S8: The original Western blot images of (I&K).
