# Supplementary figures and images for: Pan-Cancer Analysis of Prognostic and Immune Infiltrates for CXCs
Source: Cancers (Basel). 2021 Aug 18;13(16):4153. doi: 10.3390/cancers13164153 (PMC8392715; doi:10.3390/cancers13164153)

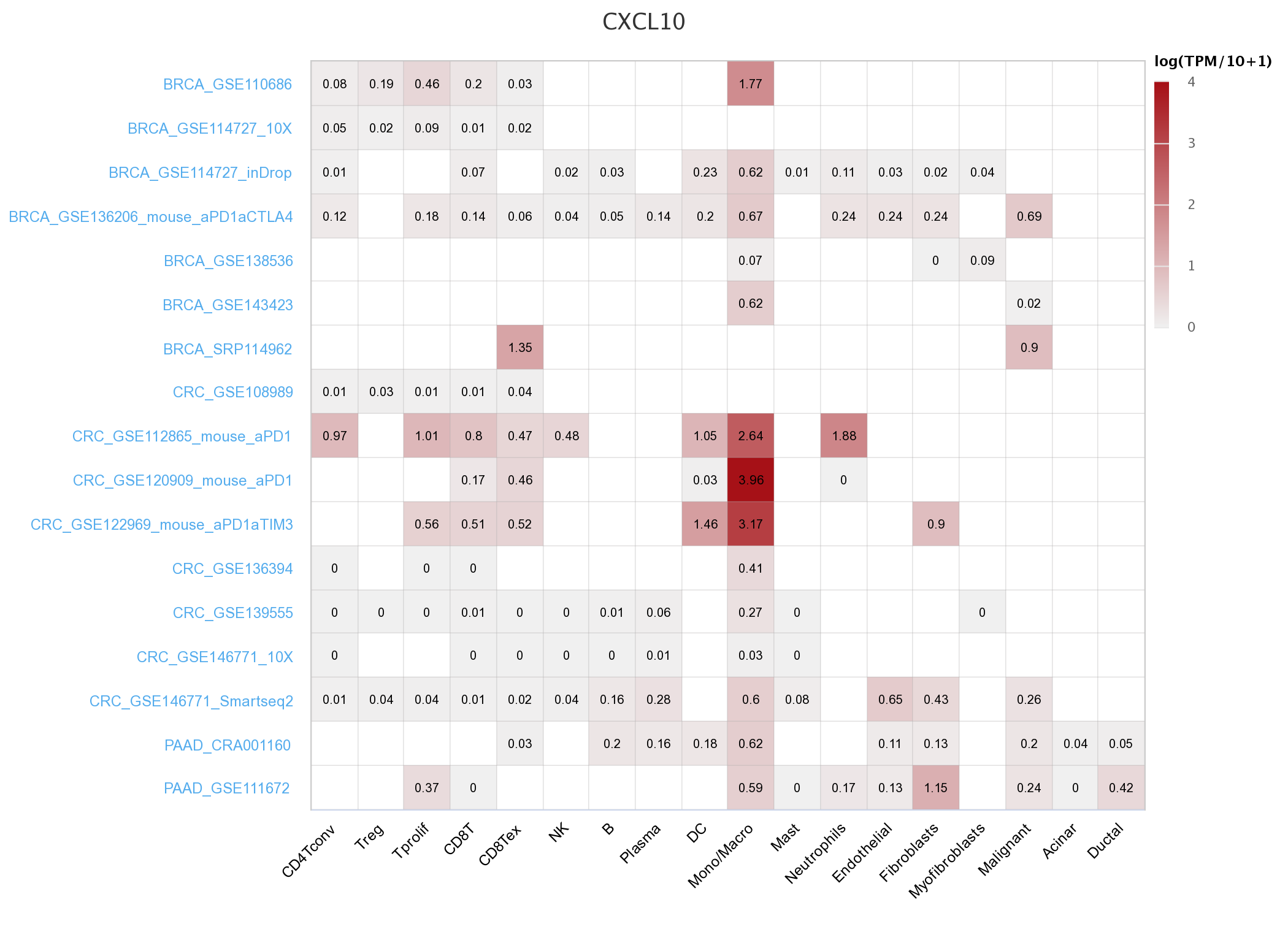

Supplement: Supplementary file 1 [file cancers-13-04153-s001.zip › Supplementary material/Material S1. Subpopulation distribution of CXCs in single-cell sequencing datasets of the three cancers/TISCH_CXCL10_heatmap.png]

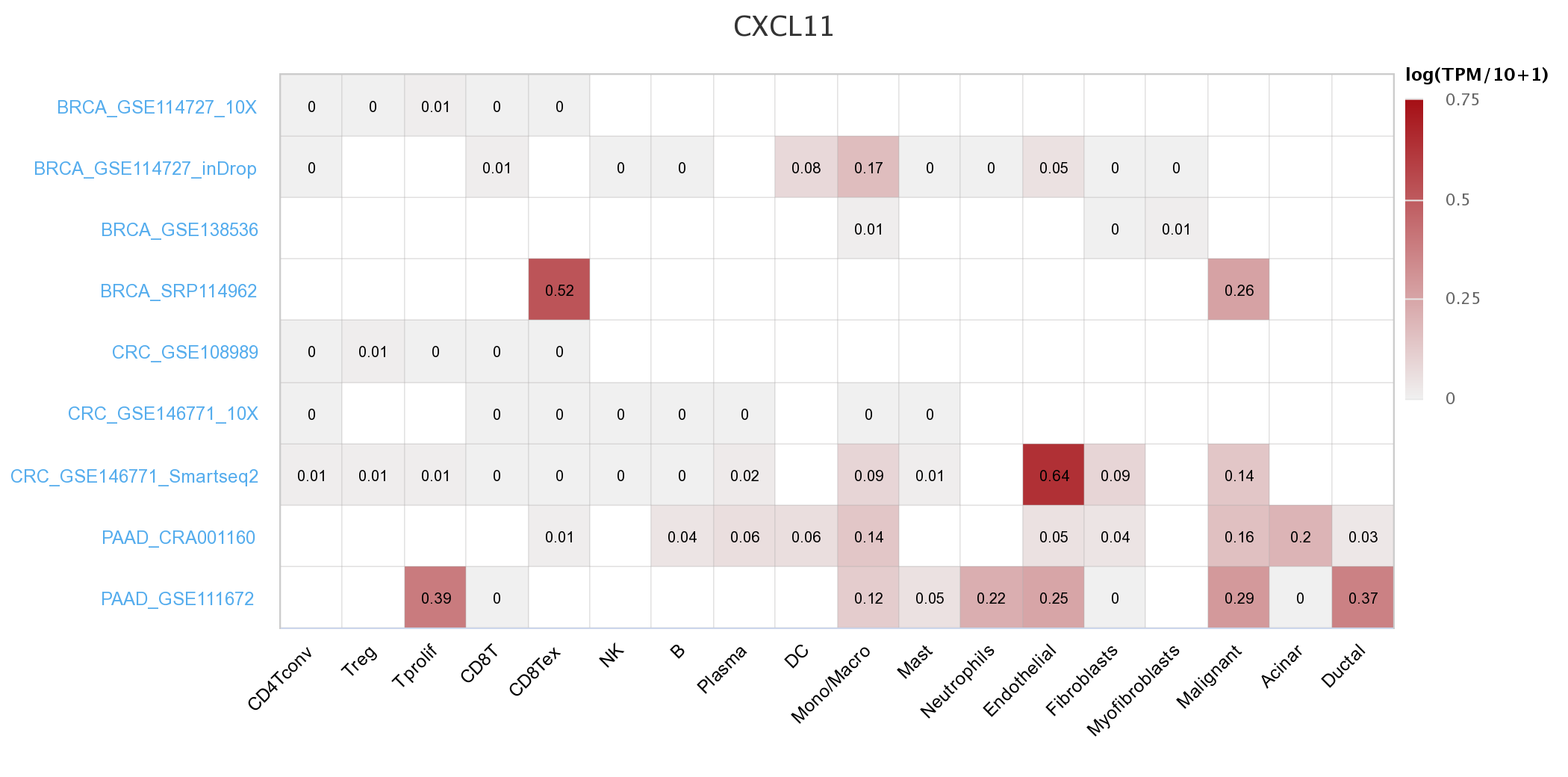

Supplement: Supplementary file 1 [file cancers-13-04153-s001.zip › Supplementary material/Material S1. Subpopulation distribution of CXCs in single-cell sequencing datasets of the three cancers/TISCH_CXCL11_heatmap.png]

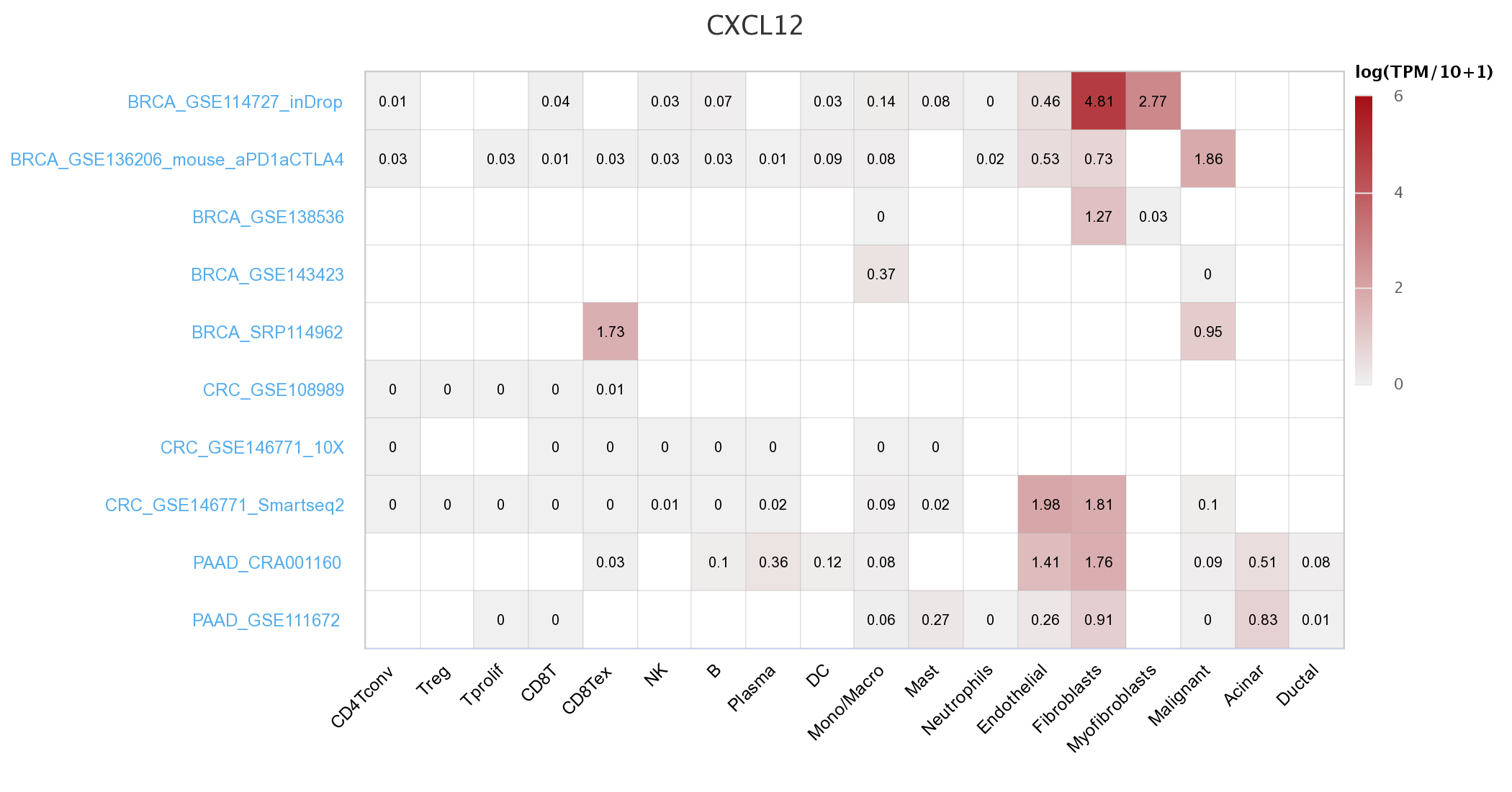

Supplement: Supplementary file 1 [file cancers-13-04153-s001.zip › Supplementary material/Material S1. Subpopulation distribution of CXCs in single-cell sequencing datasets of the three cancers/TISCH_CXCL12_heatmap.png]

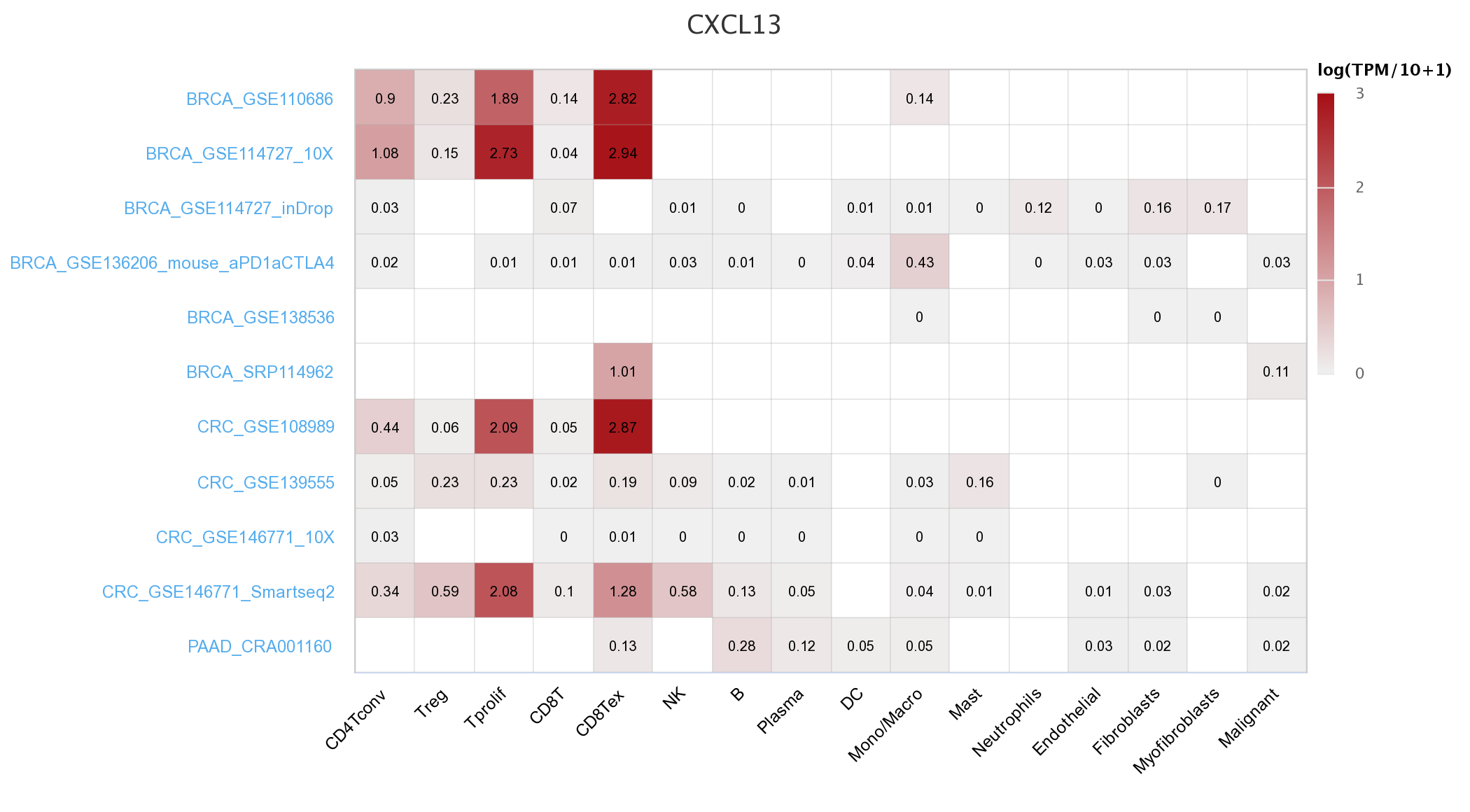

Supplement: Supplementary file 1 [file cancers-13-04153-s001.zip › Supplementary material/Material S1. Subpopulation distribution of CXCs in single-cell sequencing datasets of the three cancers/TISCH_CXCL13_heatmap.png]

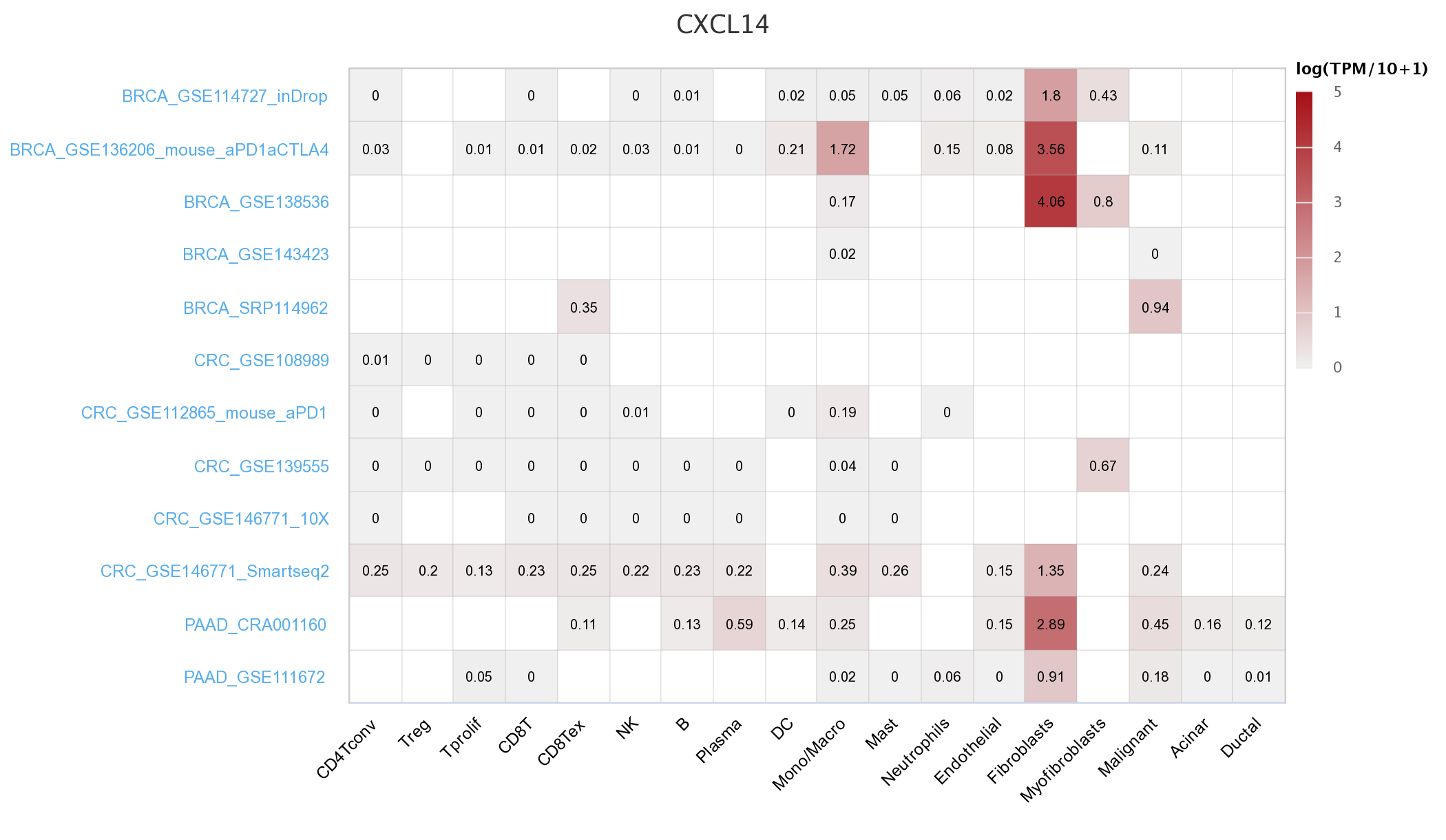

Supplement: Supplementary file 1 [file cancers-13-04153-s001.zip › Supplementary material/Material S1. Subpopulation distribution of CXCs in single-cell sequencing datasets of the three cancers/TISCH_CXCL14_heatmap.png]

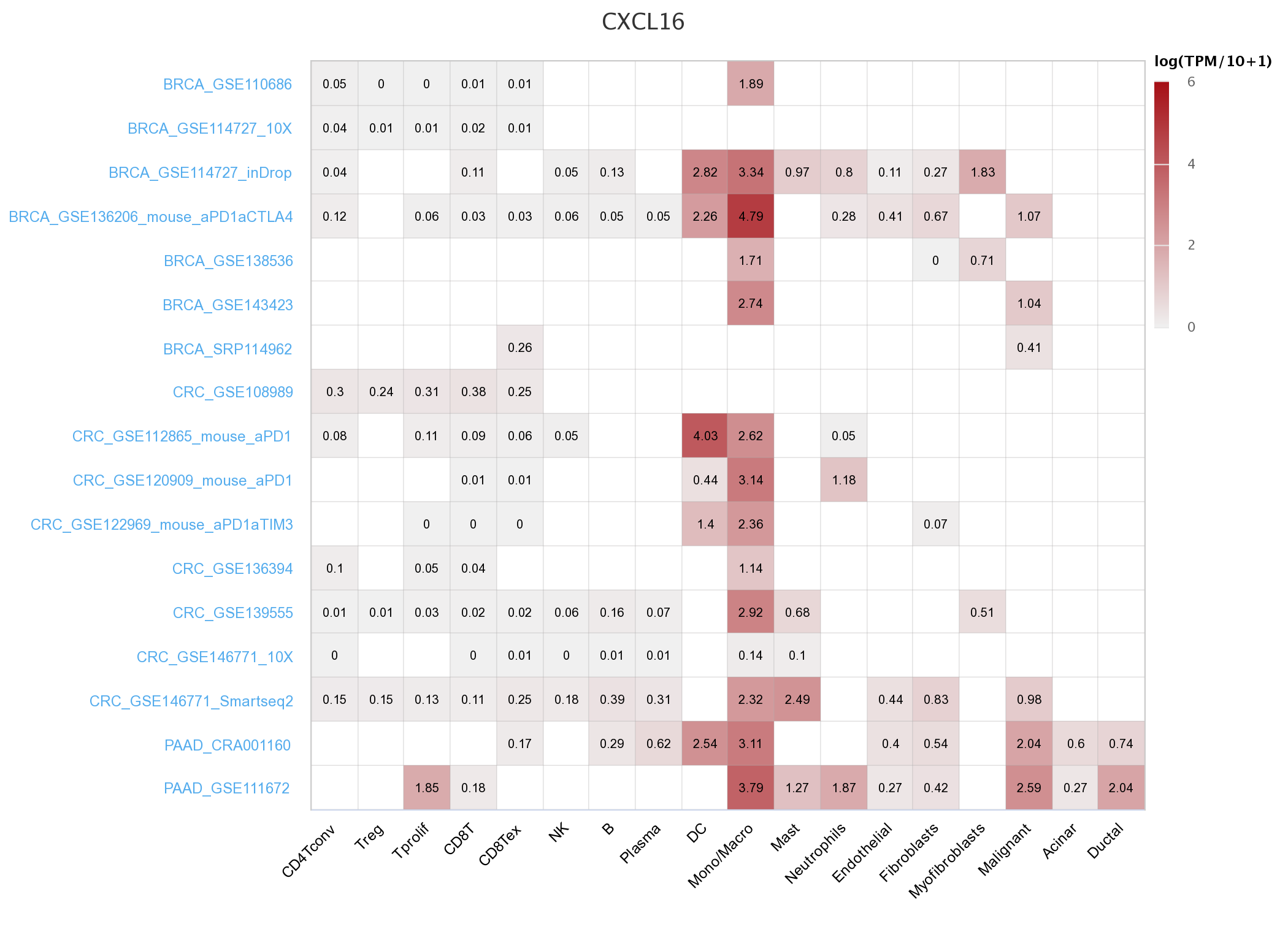

Supplement: Supplementary file 1 [file cancers-13-04153-s001.zip › Supplementary material/Material S1. Subpopulation distribution of CXCs in single-cell sequencing datasets of the three cancers/TISCH_CXCL16_heatmap.png]

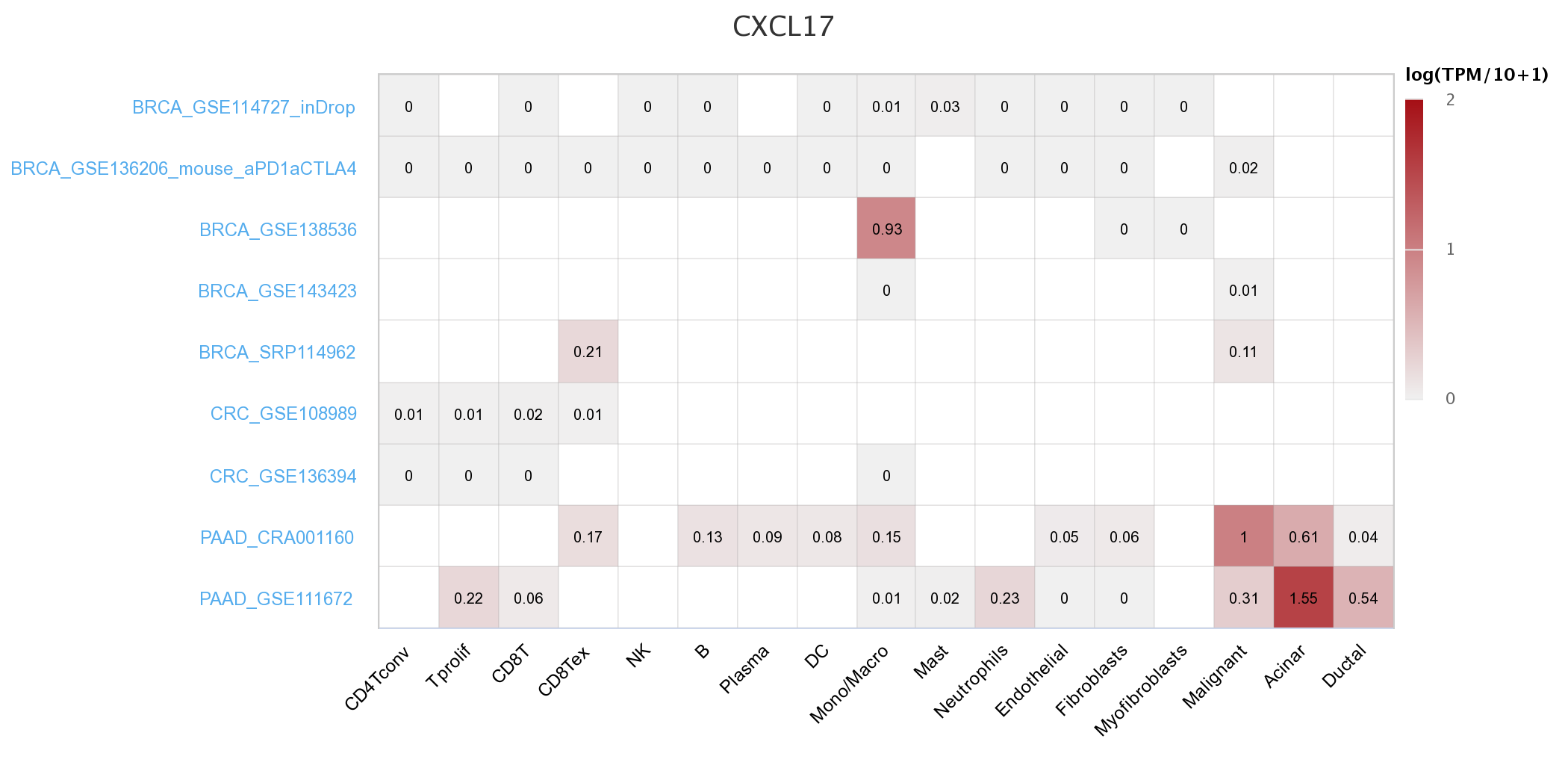

Supplement: Supplementary file 1 [file cancers-13-04153-s001.zip › Supplementary material/Material S1. Subpopulation distribution of CXCs in single-cell sequencing datasets of the three cancers/TISCH_CXCL17_heatmap.png]

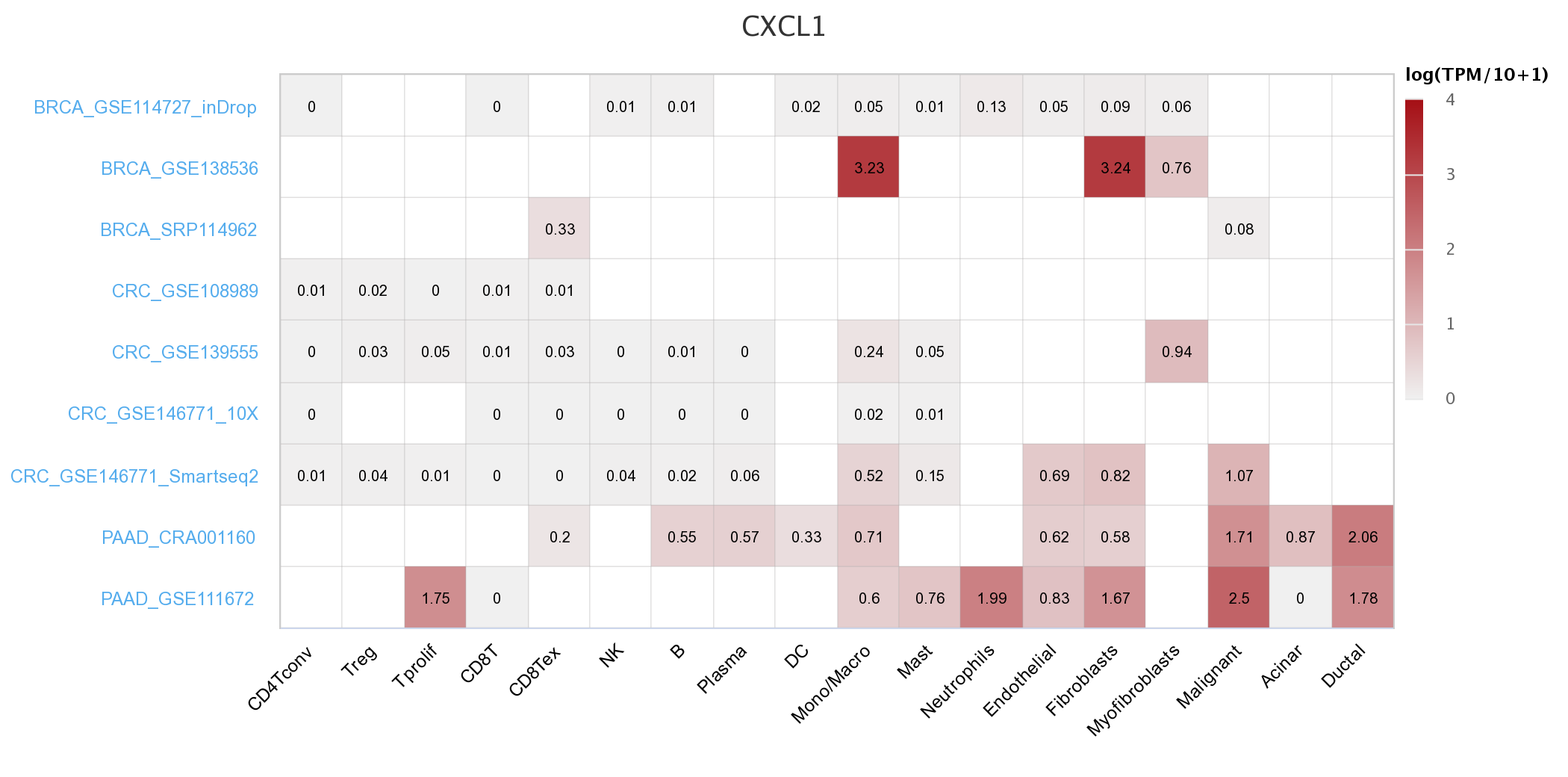

Supplement: Supplementary file 1 [file cancers-13-04153-s001.zip › Supplementary material/Material S1. Subpopulation distribution of CXCs in single-cell sequencing datasets of the three cancers/TISCH_CXCL1_heatmap.png]

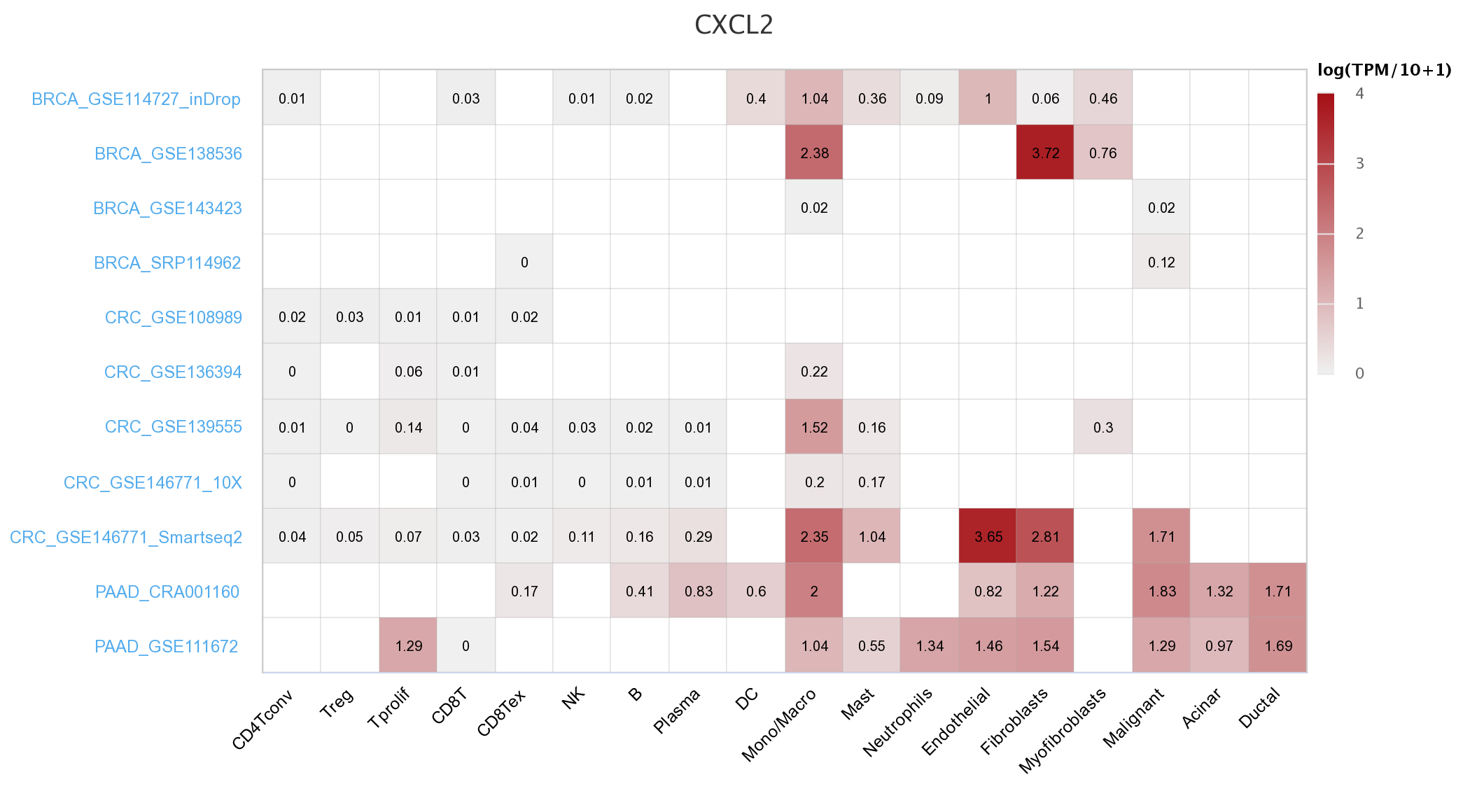

Supplement: Supplementary file 1 [file cancers-13-04153-s001.zip › Supplementary material/Material S1. Subpopulation distribution of CXCs in single-cell sequencing datasets of the three cancers/TISCH_CXCL2_heatmap.png]

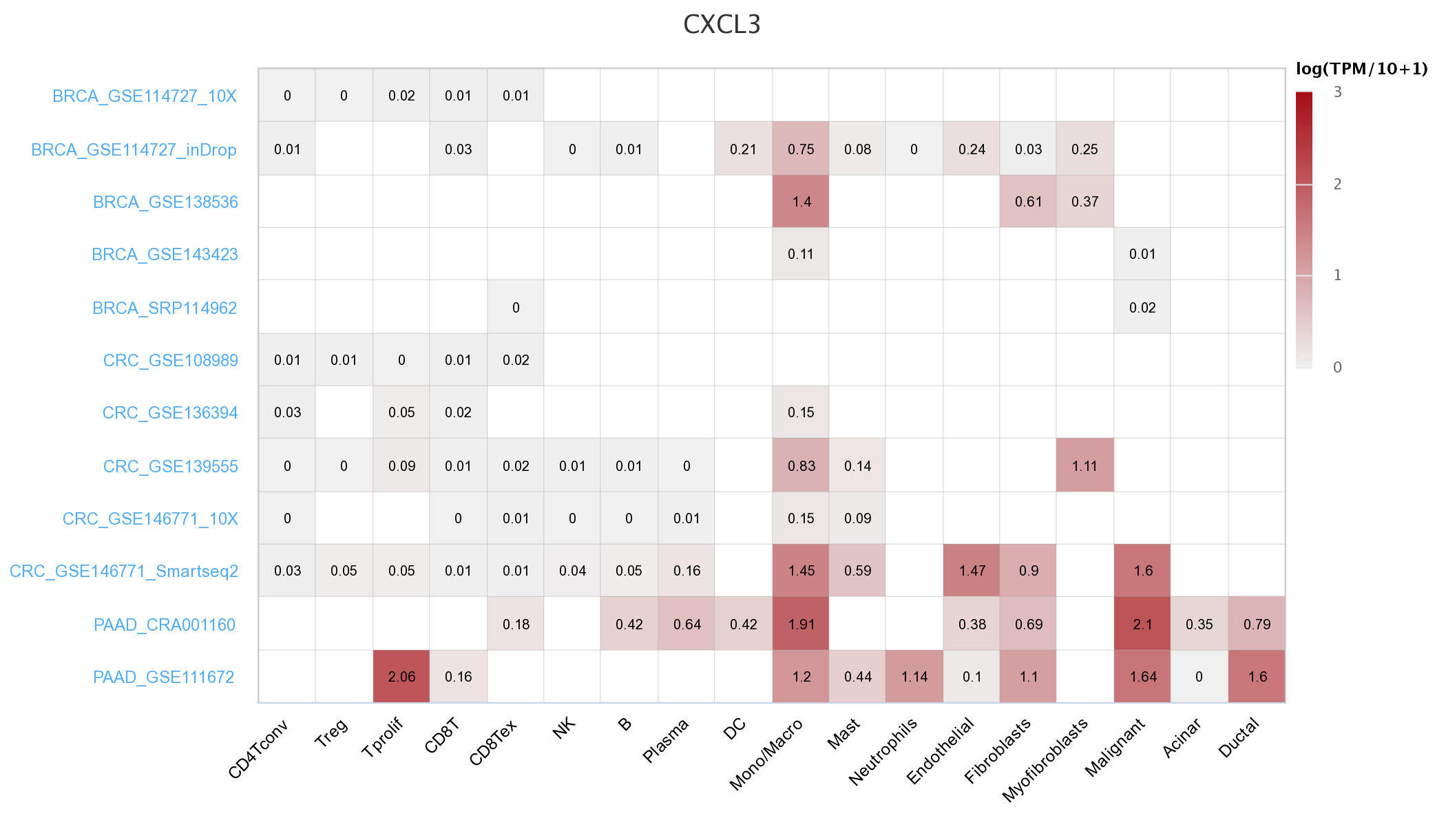

Supplement: Supplementary file 1 [file cancers-13-04153-s001.zip › Supplementary material/Material S1. Subpopulation distribution of CXCs in single-cell sequencing datasets of the three cancers/TISCH_CXCL3_heatmap.png]

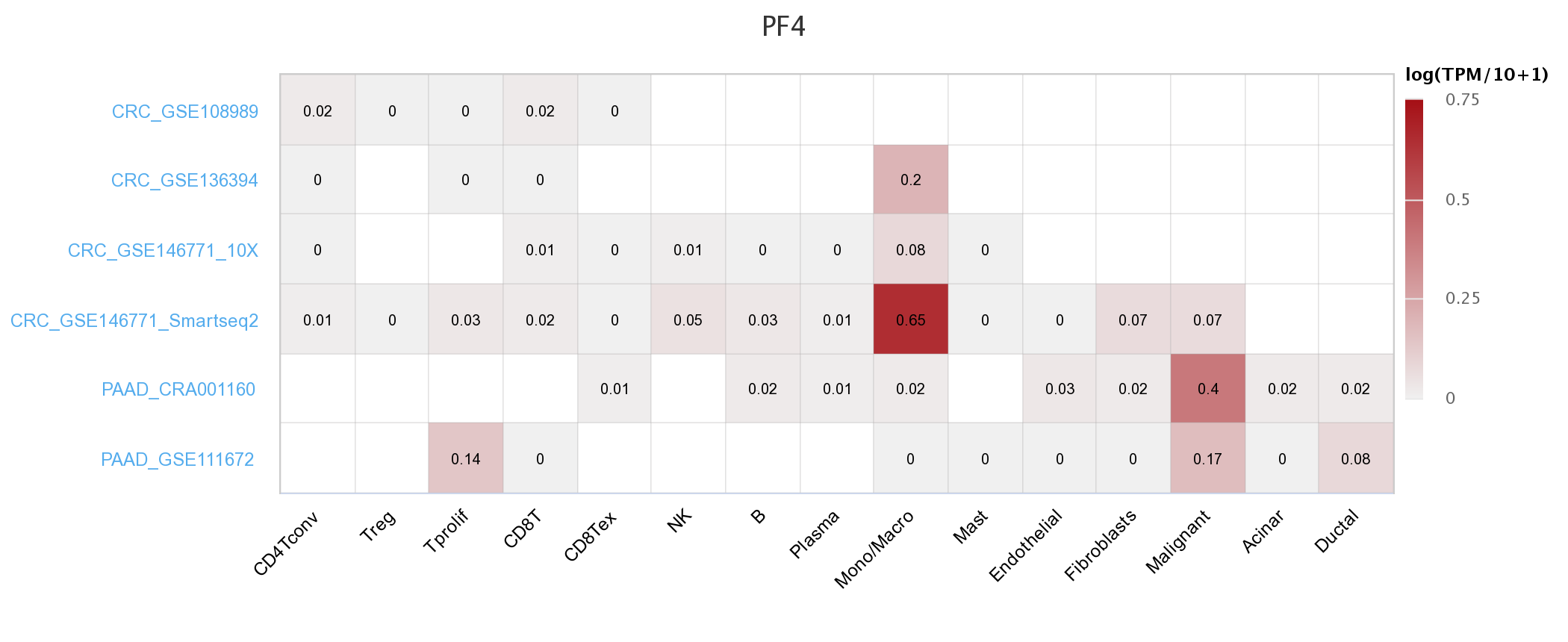

Supplement: Supplementary file 1 [file cancers-13-04153-s001.zip › Supplementary material/Material S1. Subpopulation distribution of CXCs in single-cell sequencing datasets of the three cancers/TISCH_CXCL4_heatmap.png]

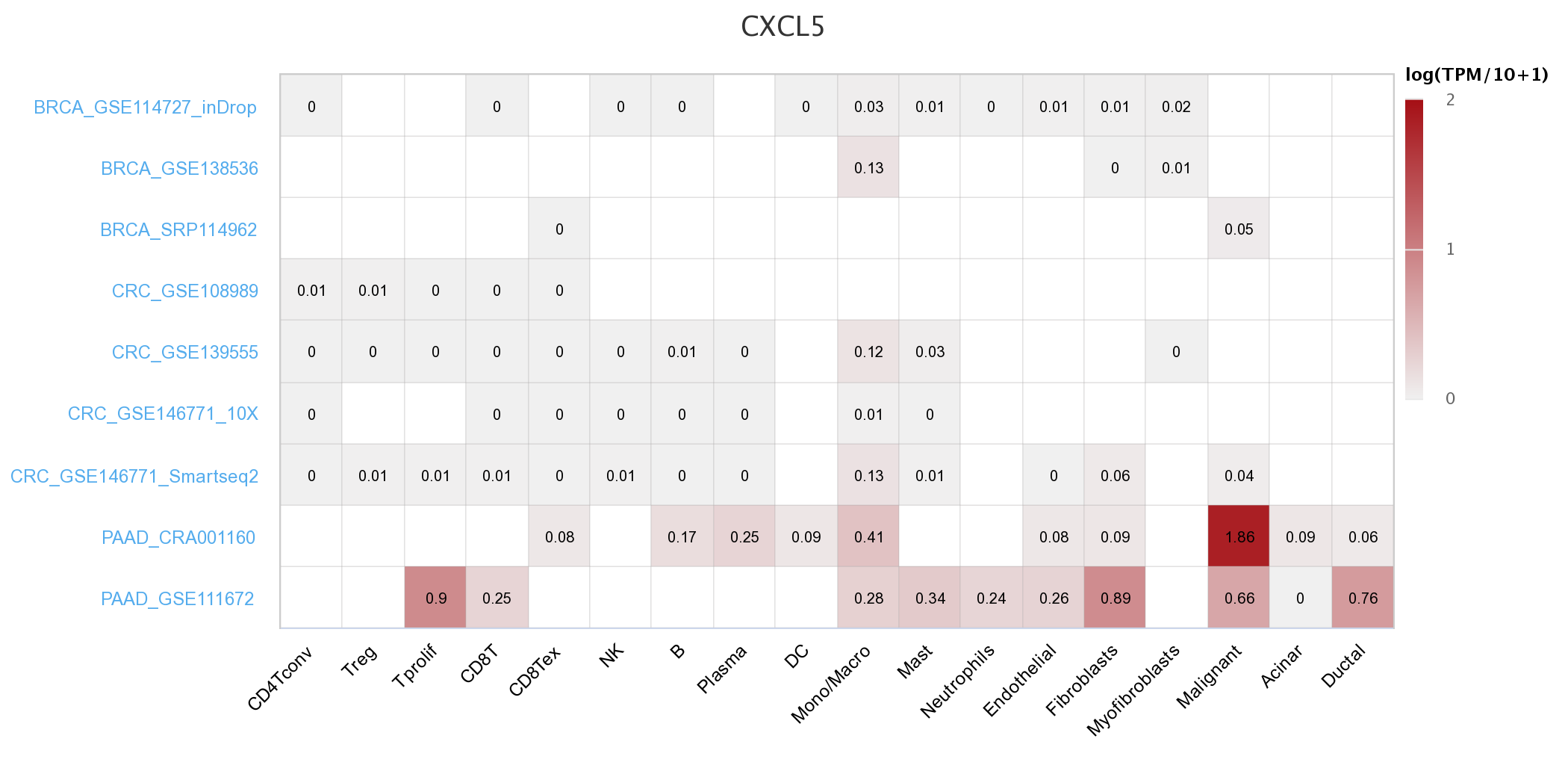

Supplement: Supplementary file 1 [file cancers-13-04153-s001.zip › Supplementary material/Material S1. Subpopulation distribution of CXCs in single-cell sequencing datasets of the three cancers/TISCH_CXCL5_heatmap.png]

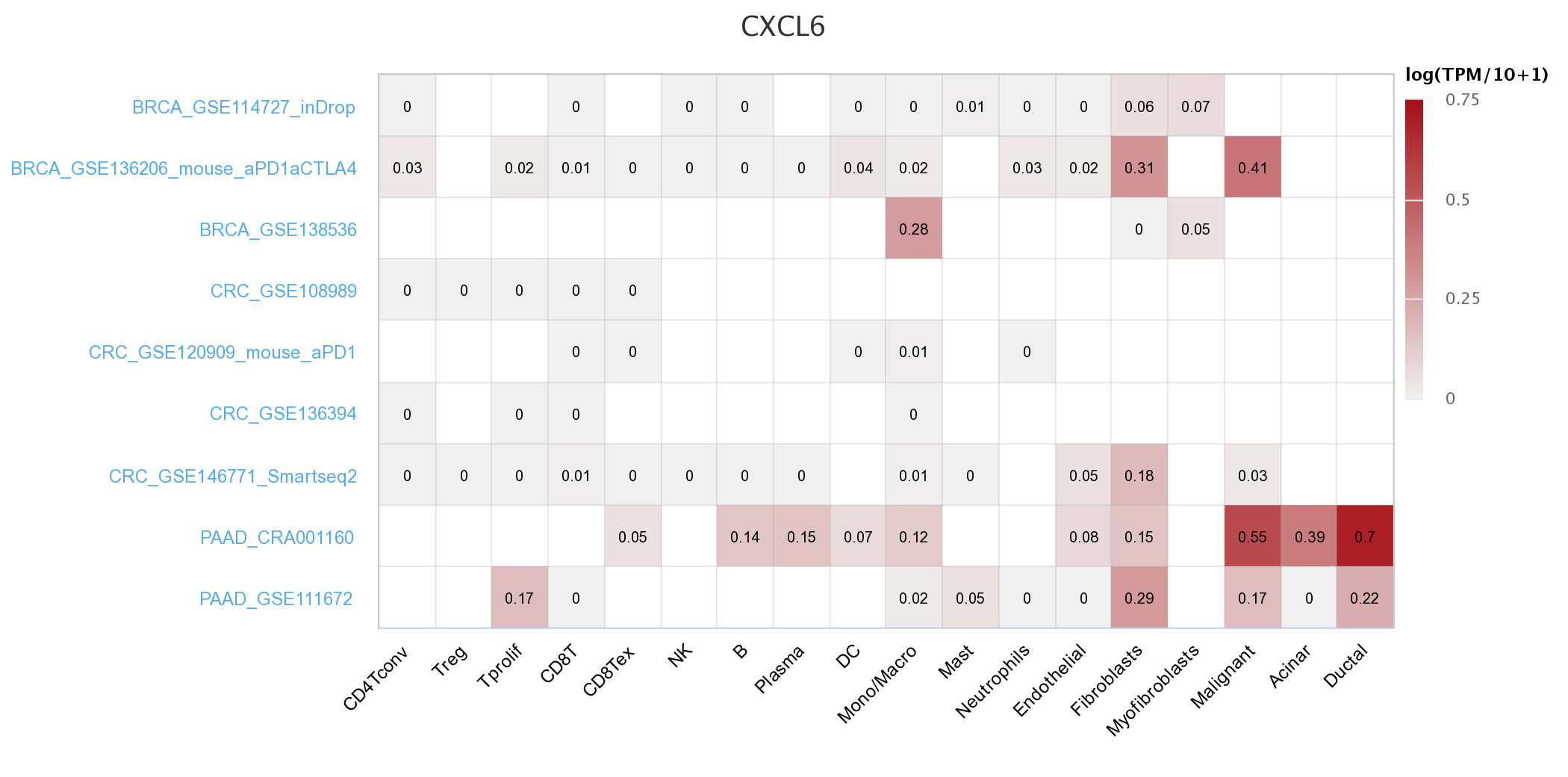

Supplement: Supplementary file 1 [file cancers-13-04153-s001.zip › Supplementary material/Material S1. Subpopulation distribution of CXCs in single-cell sequencing datasets of the three cancers/TISCH_CXCL6_heatmap.png]

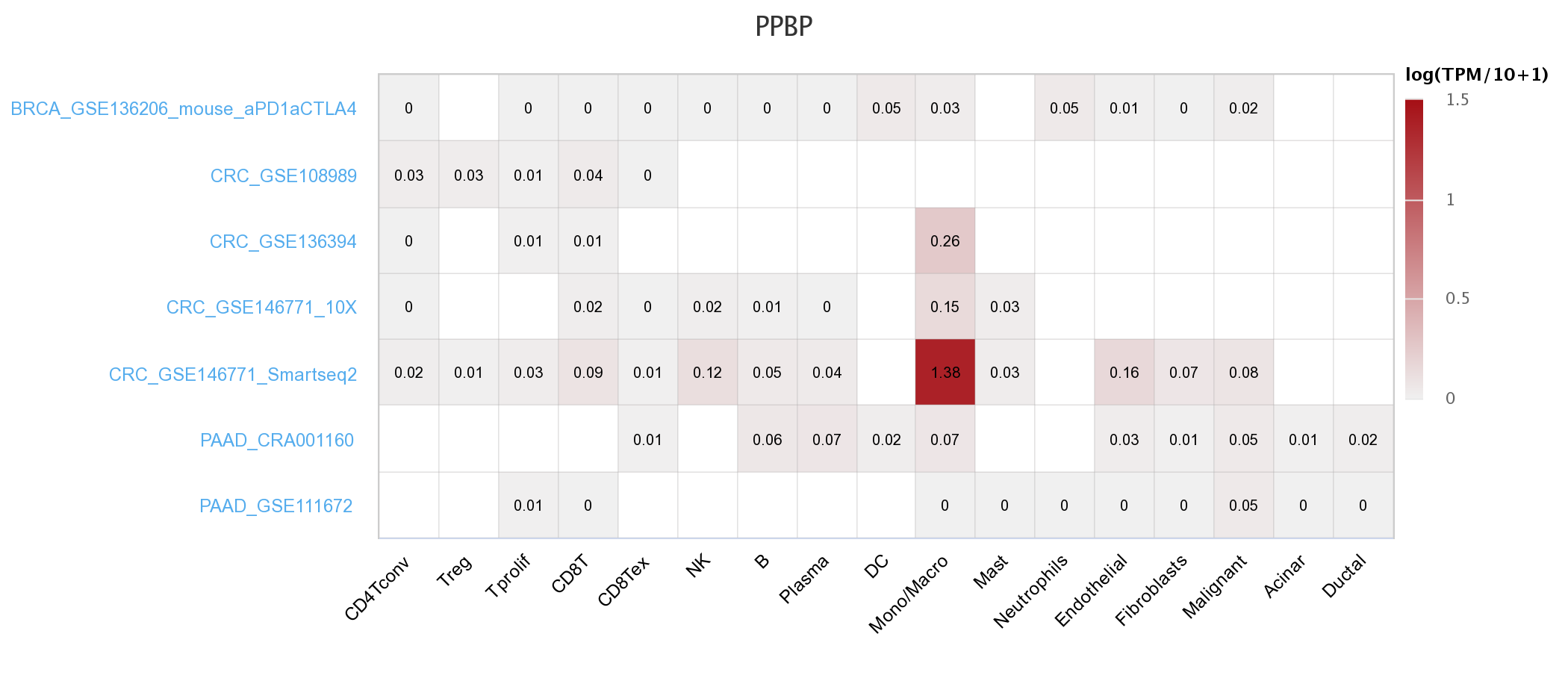

Supplement: Supplementary file 1 [file cancers-13-04153-s001.zip › Supplementary material/Material S1. Subpopulation distribution of CXCs in single-cell sequencing datasets of the three cancers/TISCH_CXCL7_heatmap.png]

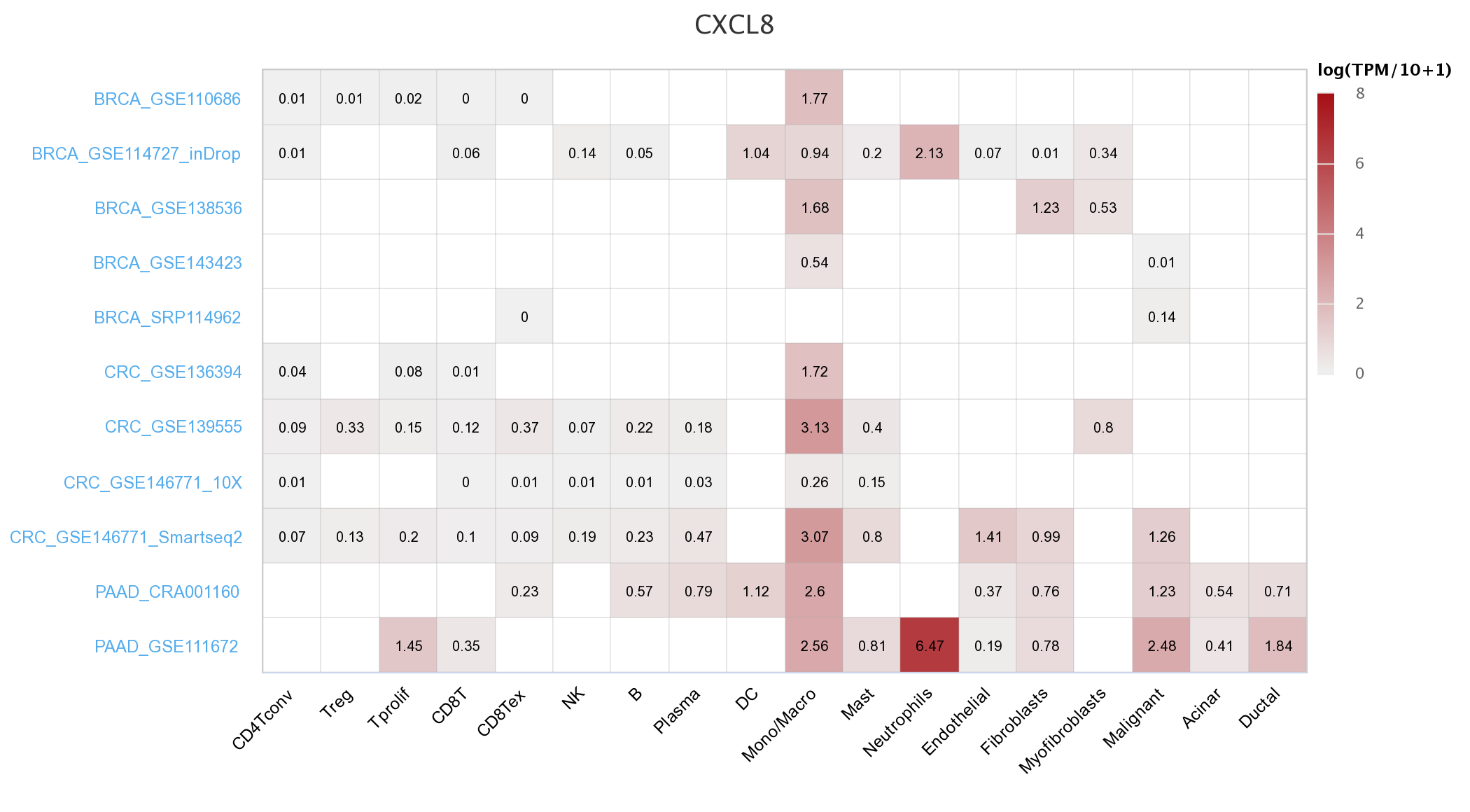

Supplement: Supplementary file 1 [file cancers-13-04153-s001.zip › Supplementary material/Material S1. Subpopulation distribution of CXCs in single-cell sequencing datasets of the three cancers/TISCH_CXCL8_heatmap.png]

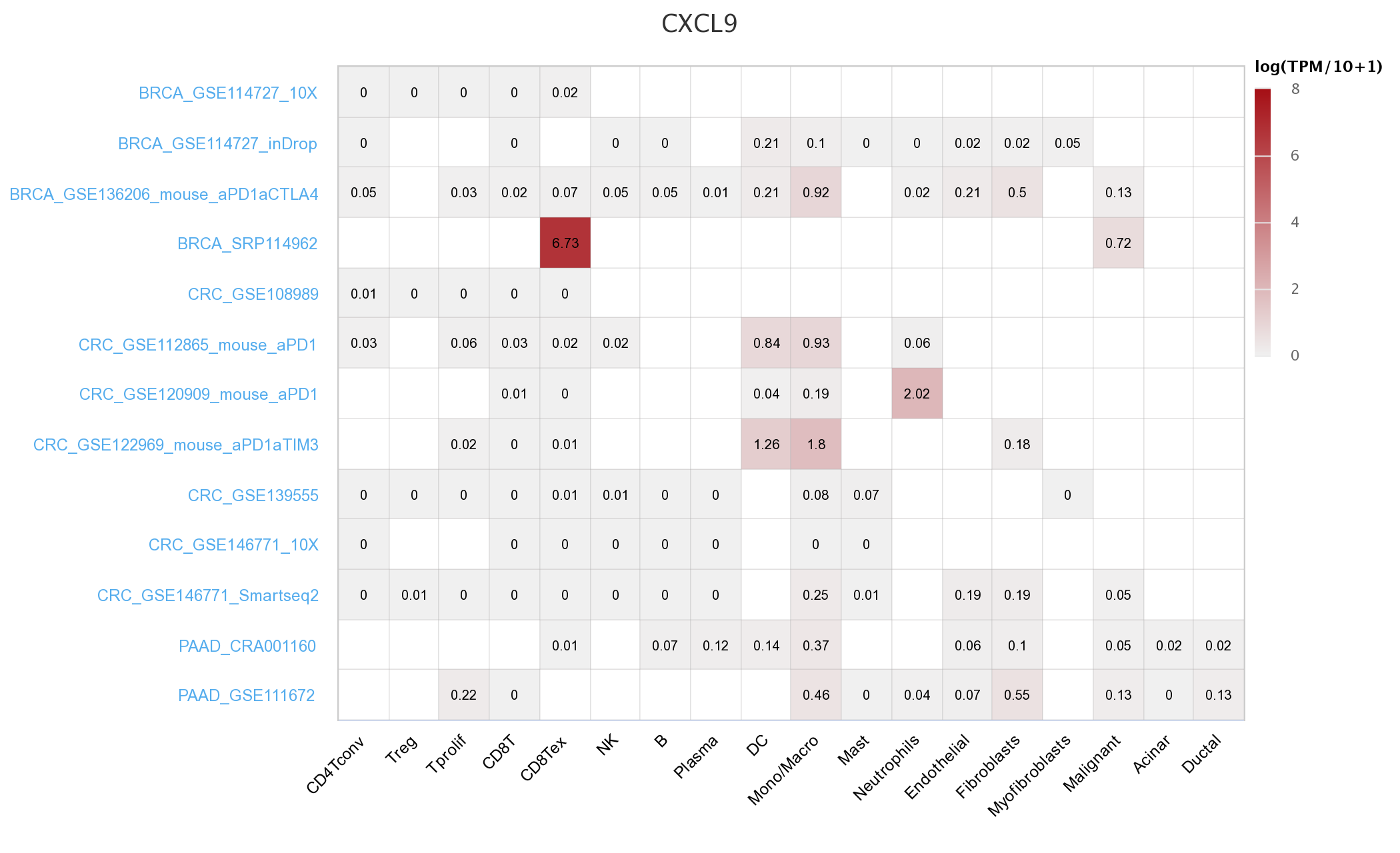

Supplement: Supplementary file 1 [file cancers-13-04153-s001.zip › Supplementary material/Material S1. Subpopulation distribution of CXCs in single-cell sequencing datasets of the three cancers/TISCH_CXCL9_heatmap.png]

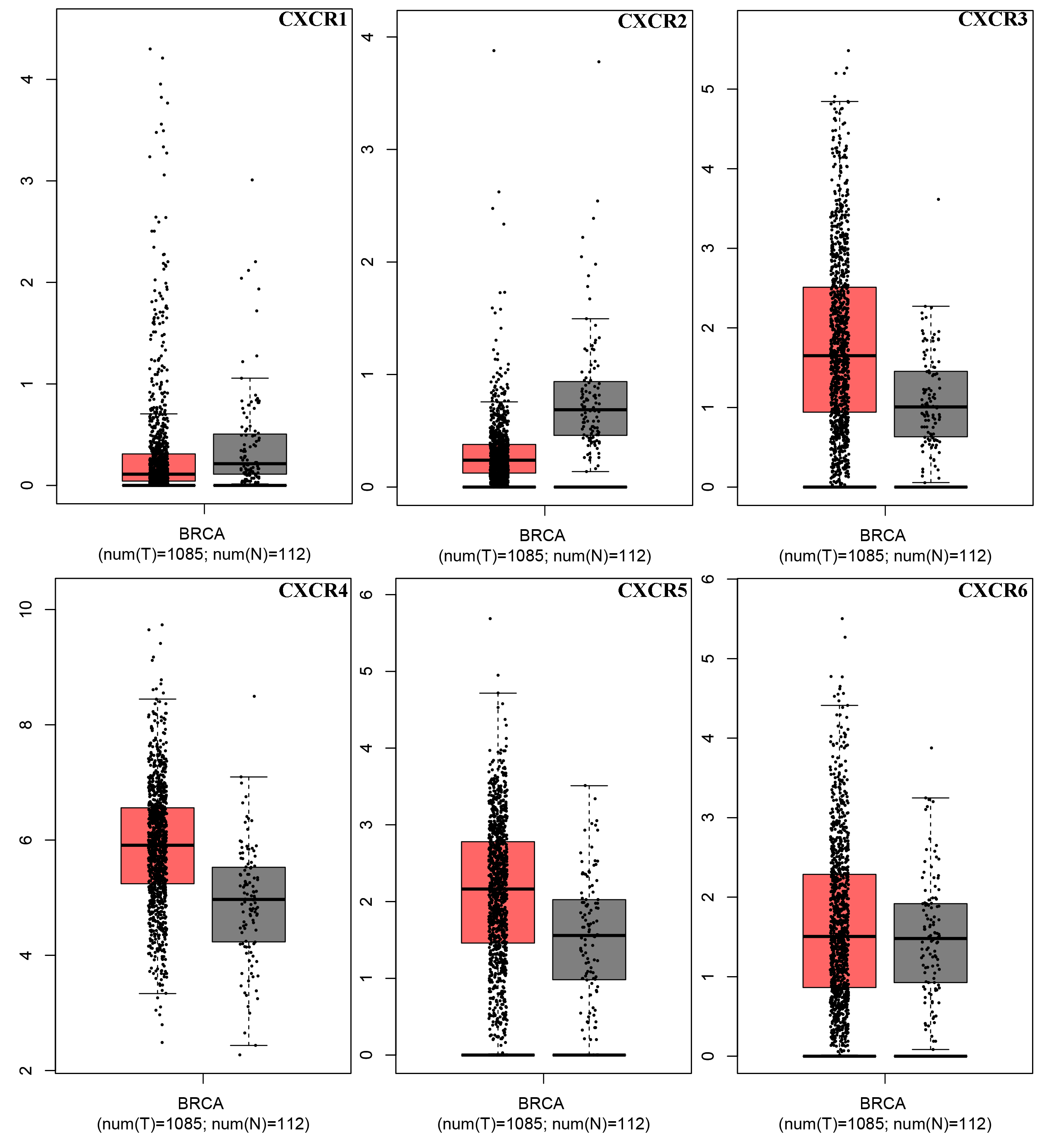

Supplement: Supplementary file 1 [file cancers-13-04153-s001.zip › Supplementary material/Material S2. Expression differences of CXCRs in three cancers/CXCR-BRCA.tif]

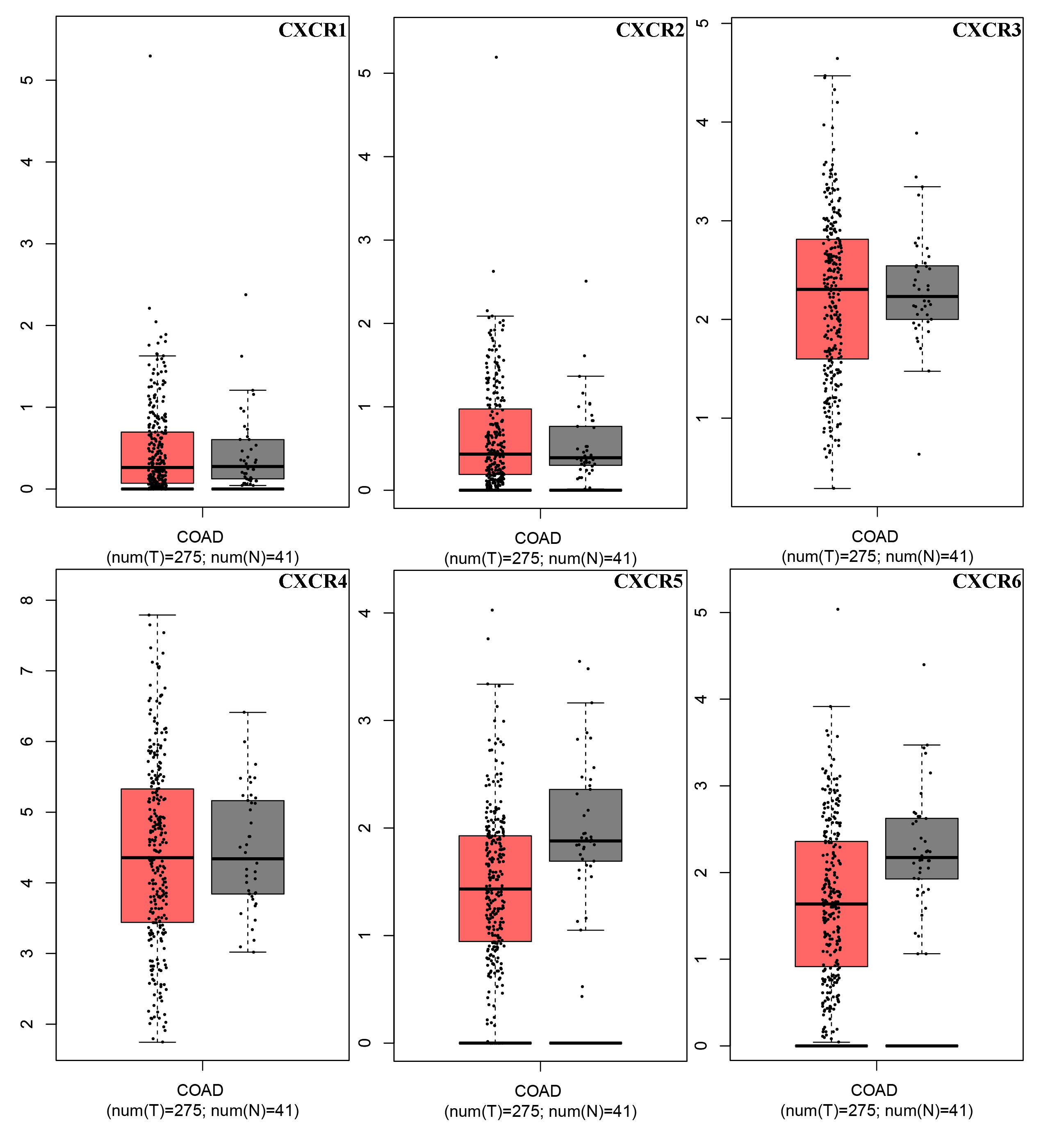

Supplement: Supplementary file 1 [file cancers-13-04153-s001.zip › Supplementary material/Material S2. Expression differences of CXCRs in three cancers/CXCR-COAD.tif]

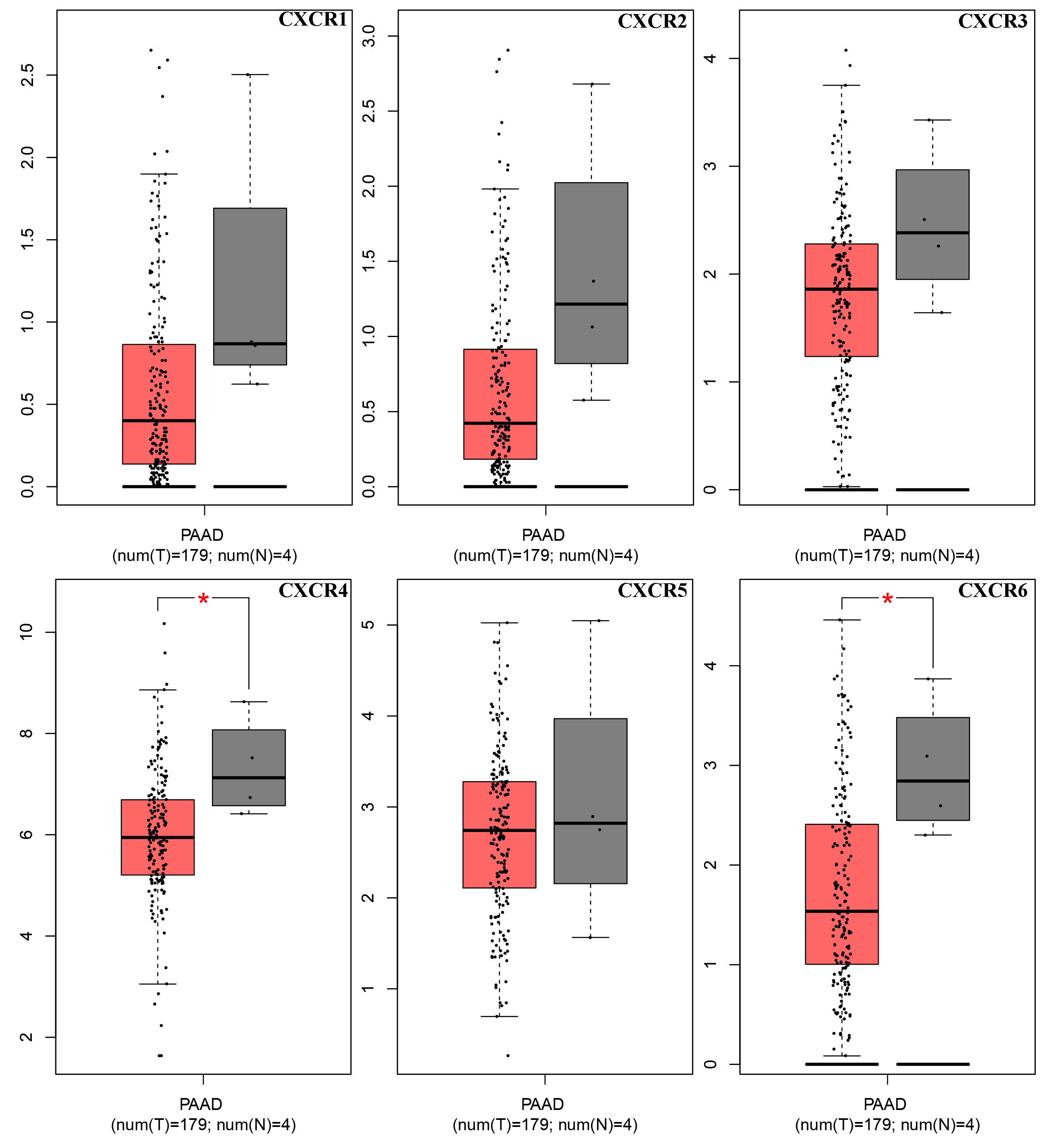

Supplement: Supplementary file 1 [file cancers-13-04153-s001.zip › Supplementary material/Material S2. Expression differences of CXCRs in three cancers/CXCR-PAAD.tif]

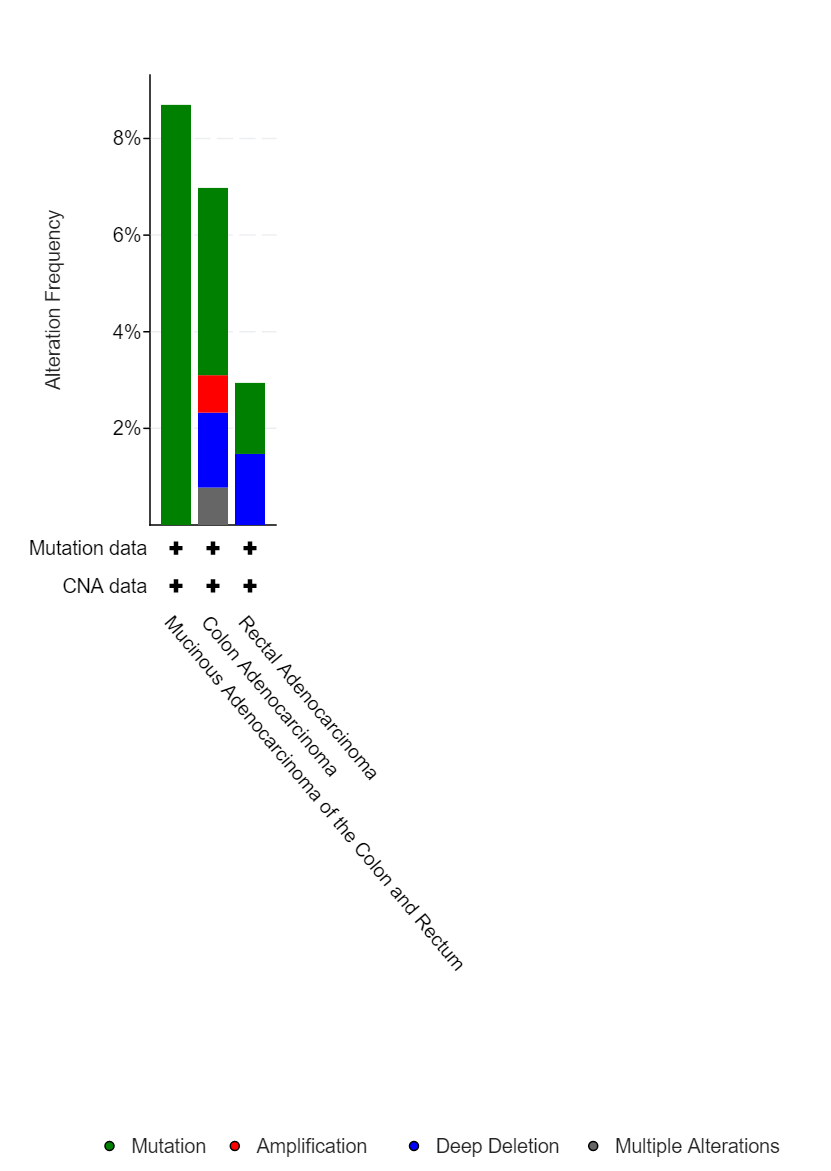

Supplement: Supplementary file 1 [file cancers-13-04153-s001.zip › Supplementary material/Material S4. CXC gene expression and mutation analysis in pancreatic cancer and colon cancer/COAD_cancer_types_summary.png]

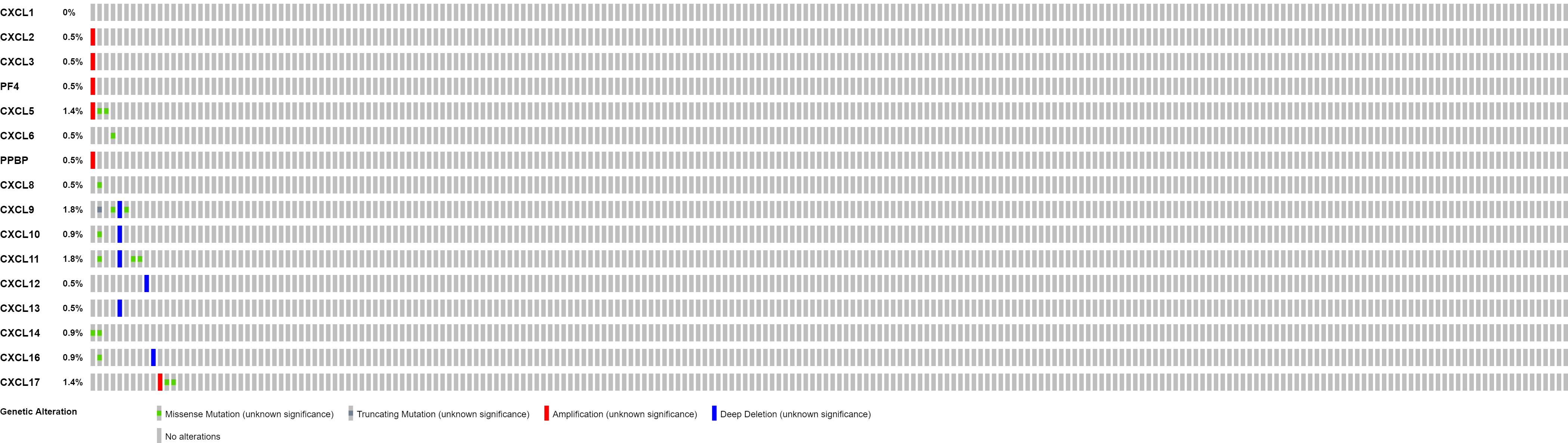

Supplement: Supplementary file 1 [file cancers-13-04153-s001.zip › Supplementary material/Material S4. CXC gene expression and mutation analysis in pancreatic cancer and colon cancer/COAD_oncoprint.png]

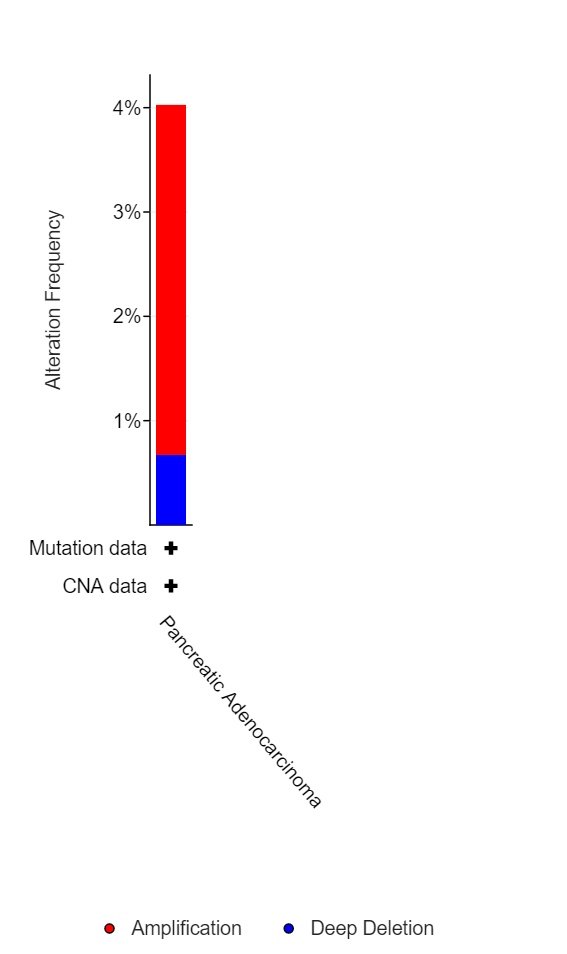

Supplement: Supplementary file 1 [file cancers-13-04153-s001.zip › Supplementary material/Material S4. CXC gene expression and mutation analysis in pancreatic cancer and colon cancer/PDAC_cancer_types_summary.png]

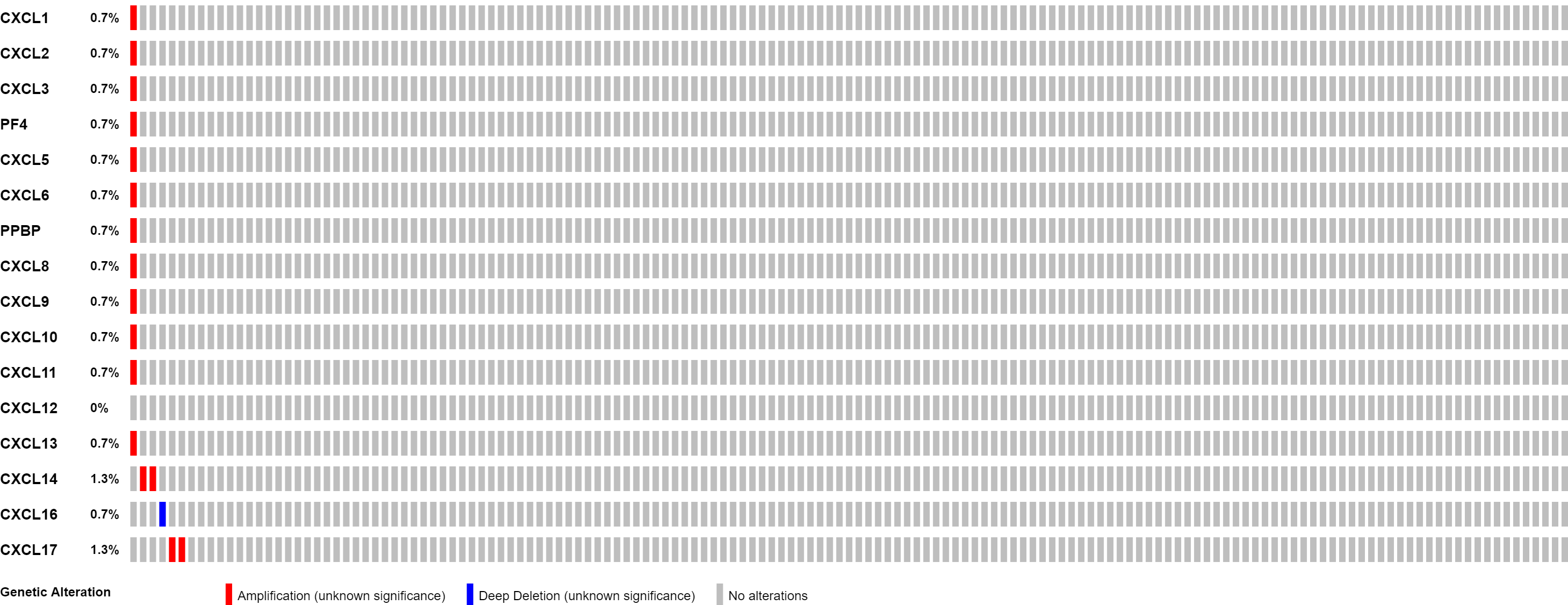

Supplement: Supplementary file 1 [file cancers-13-04153-s001.zip › Supplementary material/Material S4. CXC gene expression and mutation analysis in pancreatic cancer and colon cancer/PDAC_oncoprint.png]

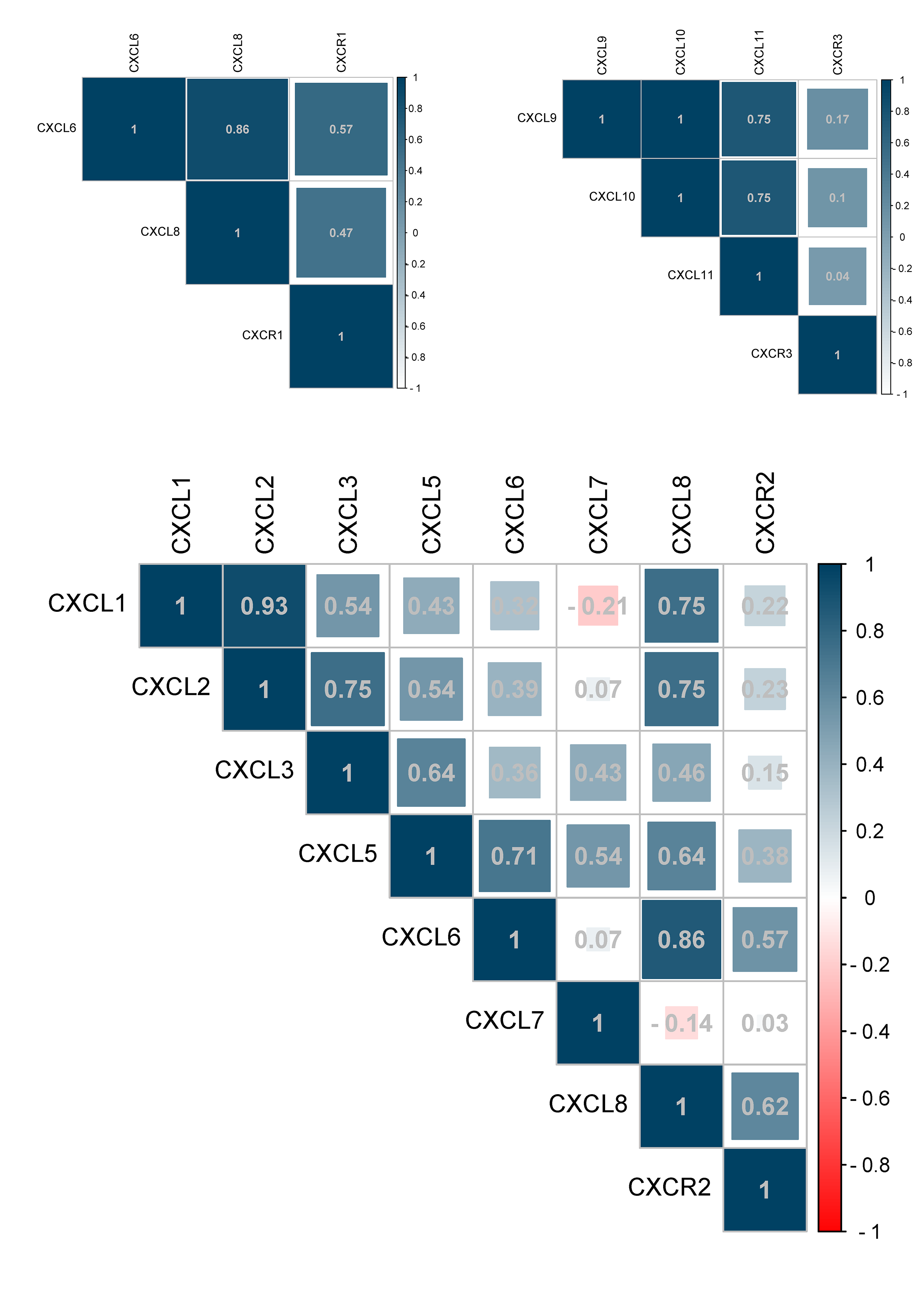

Supplement: Supplementary file 1 [file cancers-13-04153-s001.zip › Supplementary material/Material S5. Correlation analysis of CXCRs and CXCs in colon cancer/Supplement5.tif]

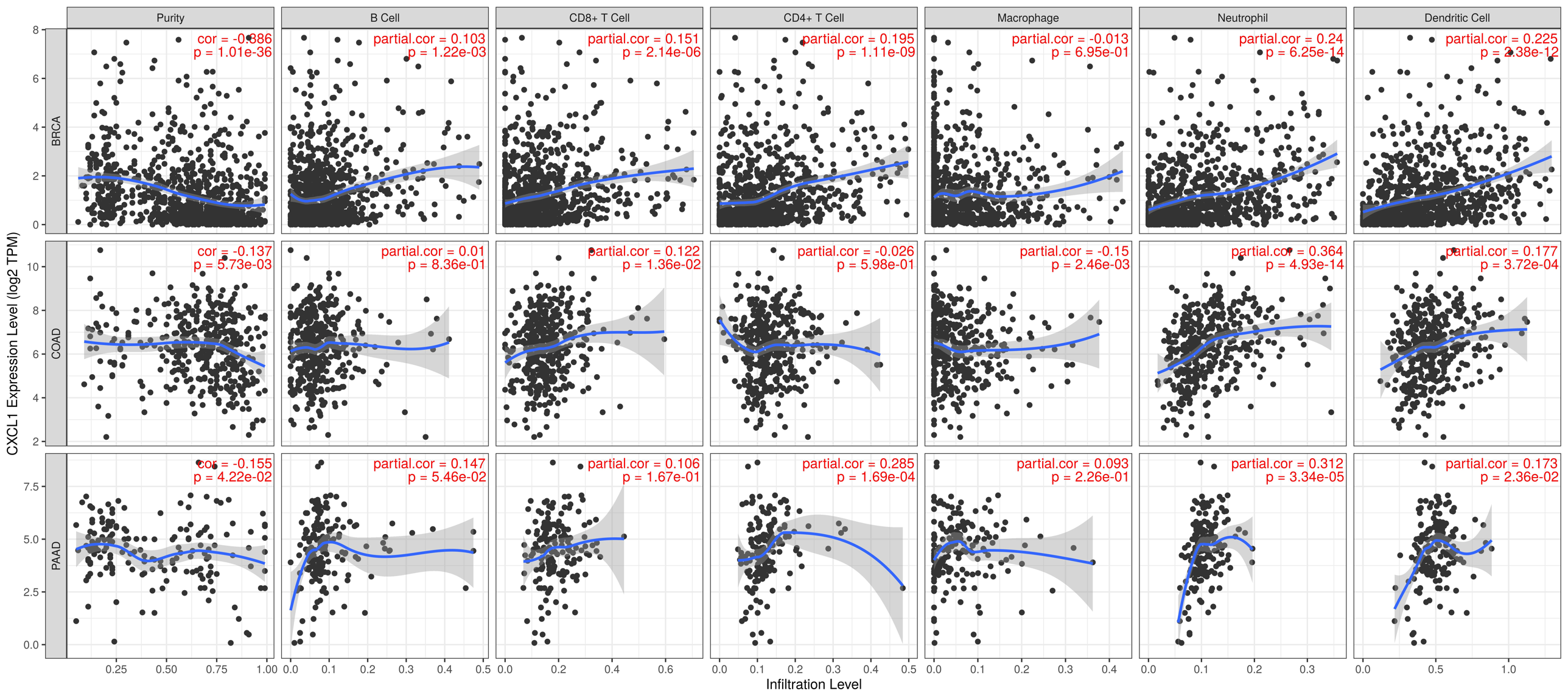

Supplement: Supplementary file 1 [file cancers-13-04153-s001.zip › Supplementary material/Material S6. Correlation of other differentially expressed CXCs and immune cell infiltration in BRCA, COAD, and PDAC/CXCL1.jpg]

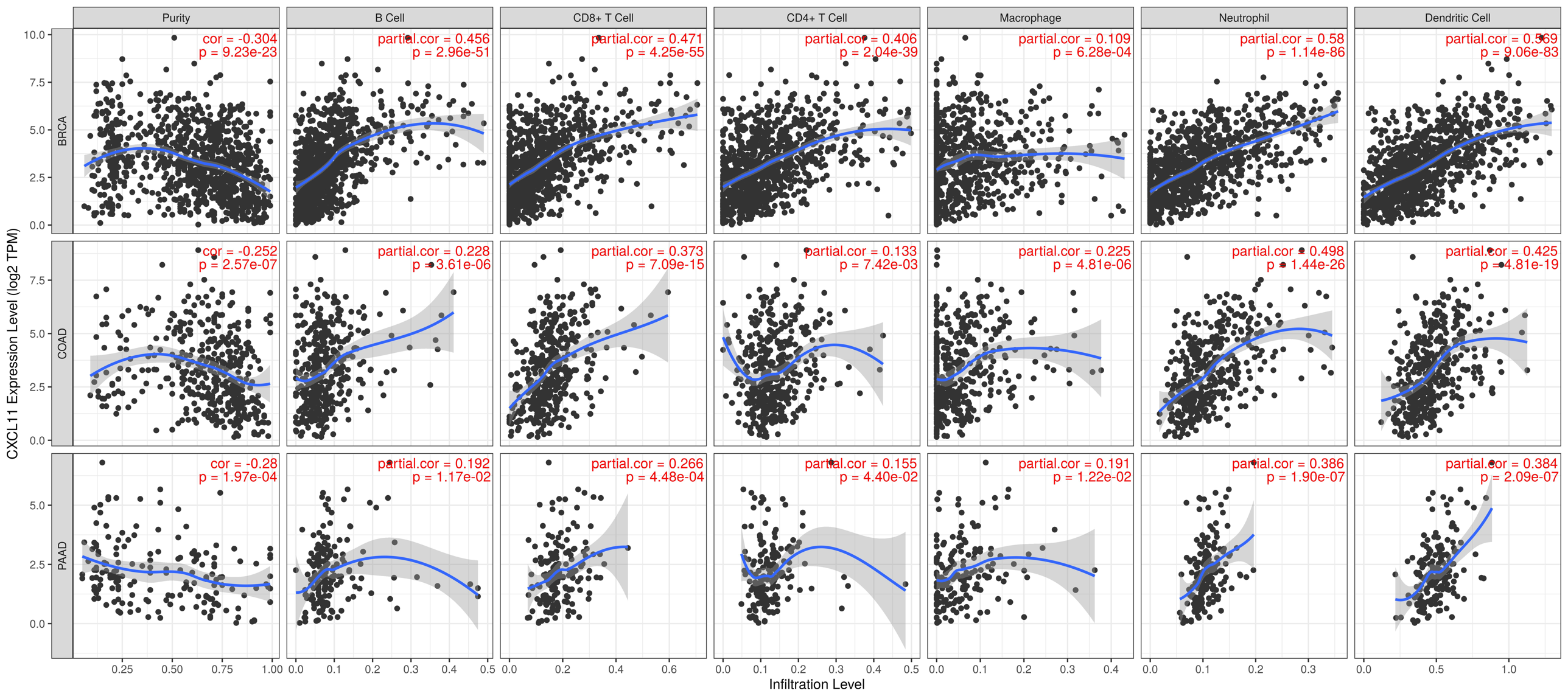

Supplement: Supplementary file 1 [file cancers-13-04153-s001.zip › Supplementary material/Material S6. Correlation of other differentially expressed CXCs and immune cell infiltration in BRCA, COAD, and PDAC/CXCL11.jpg]

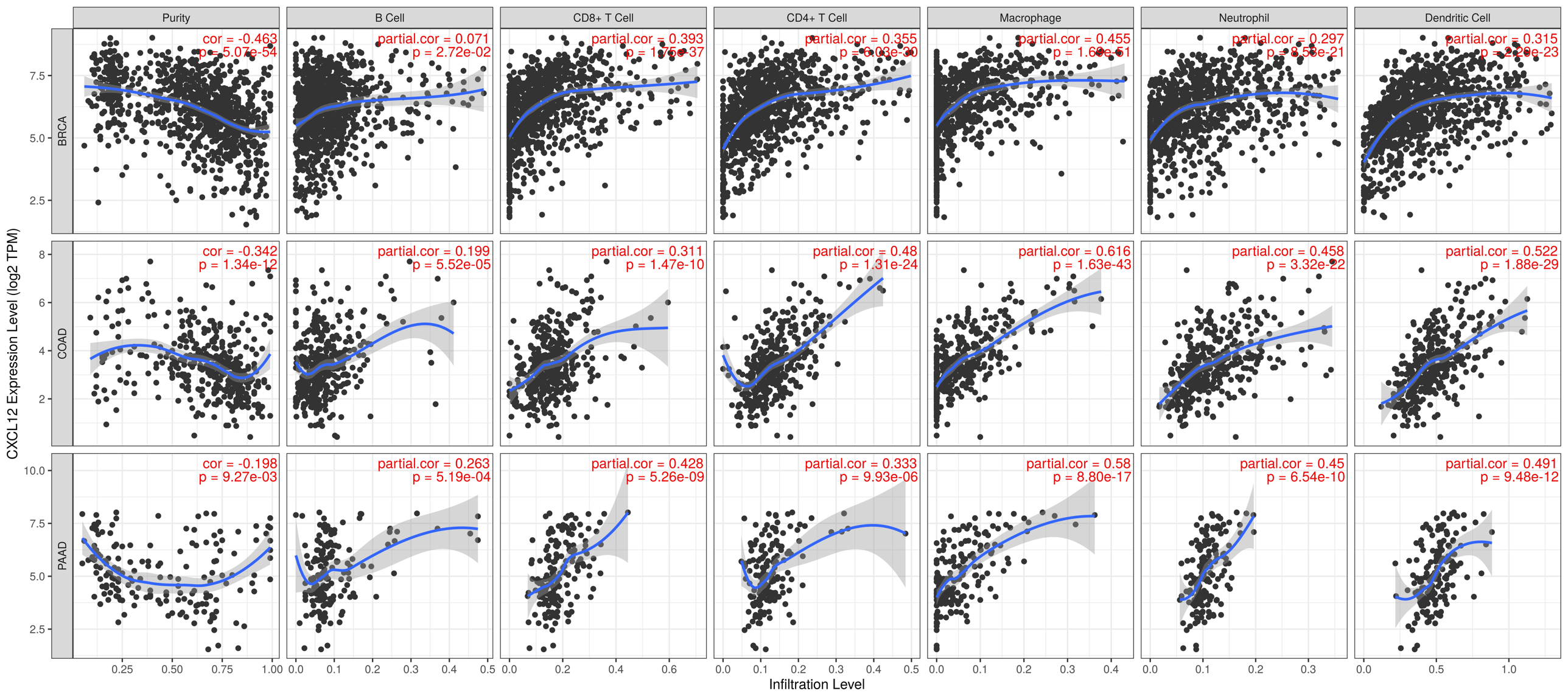

Supplement: Supplementary file 1 [file cancers-13-04153-s001.zip › Supplementary material/Material S6. Correlation of other differentially expressed CXCs and immune cell infiltration in BRCA, COAD, and PDAC/CXCL12.jpg]

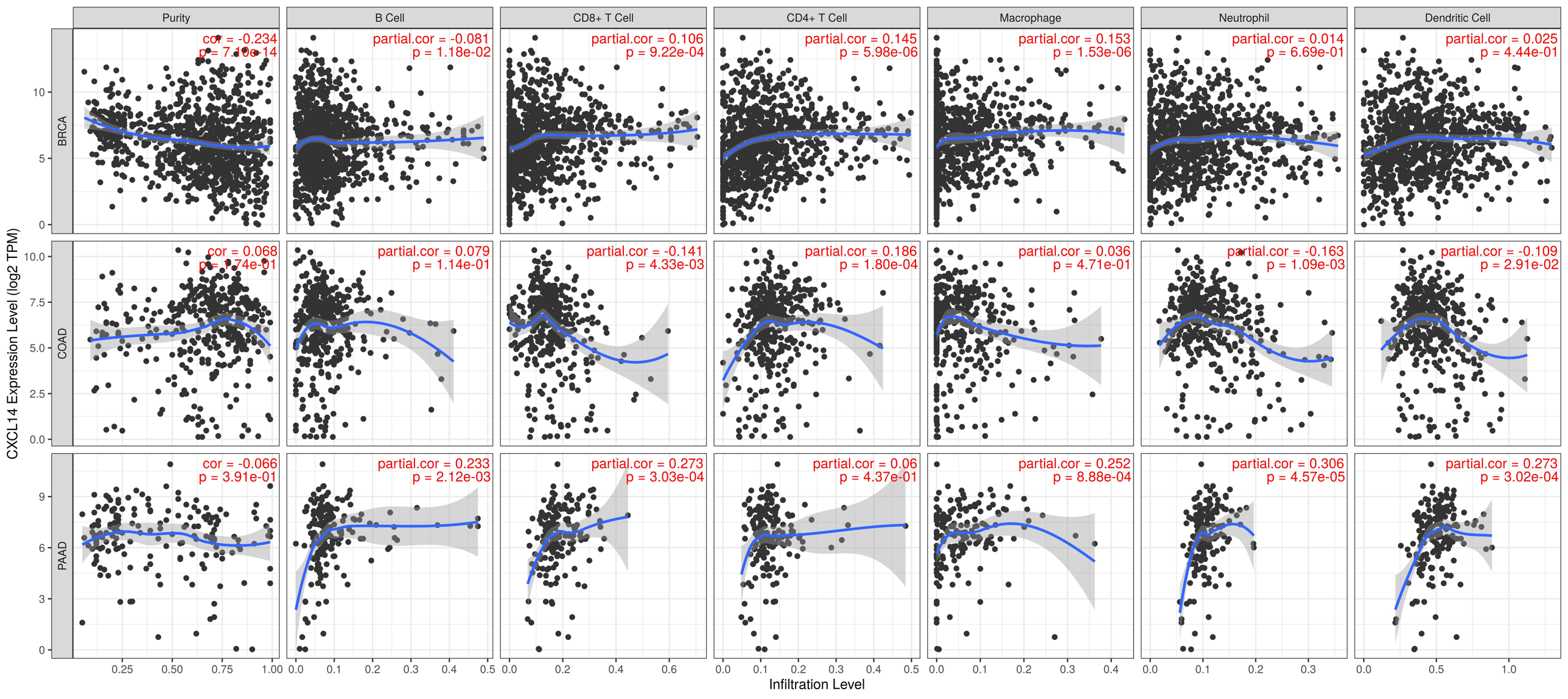

Supplement: Supplementary file 1 [file cancers-13-04153-s001.zip › Supplementary material/Material S6. Correlation of other differentially expressed CXCs and immune cell infiltration in BRCA, COAD, and PDAC/CXCL14.jpg]

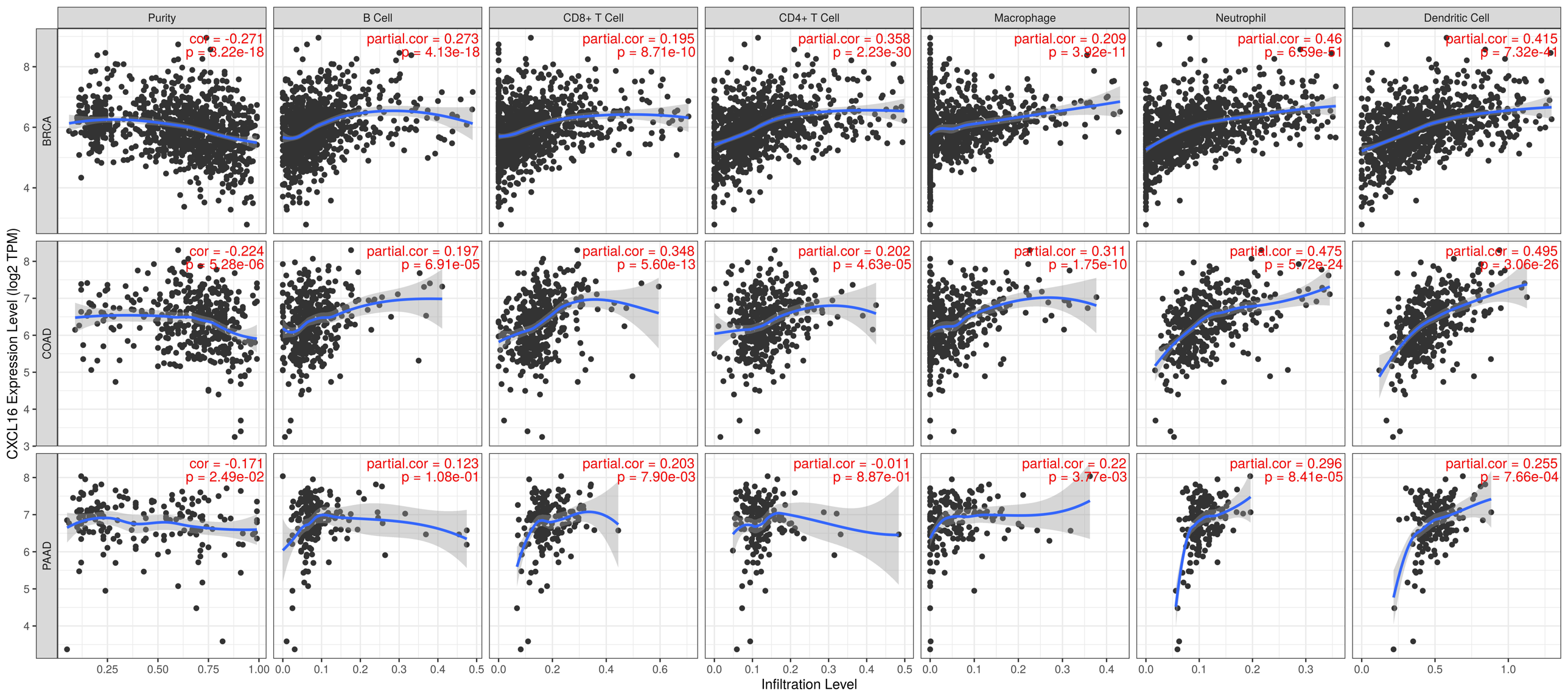

Supplement: Supplementary file 1 [file cancers-13-04153-s001.zip › Supplementary material/Material S6. Correlation of other differentially expressed CXCs and immune cell infiltration in BRCA, COAD, and PDAC/CXCL16.jpg]

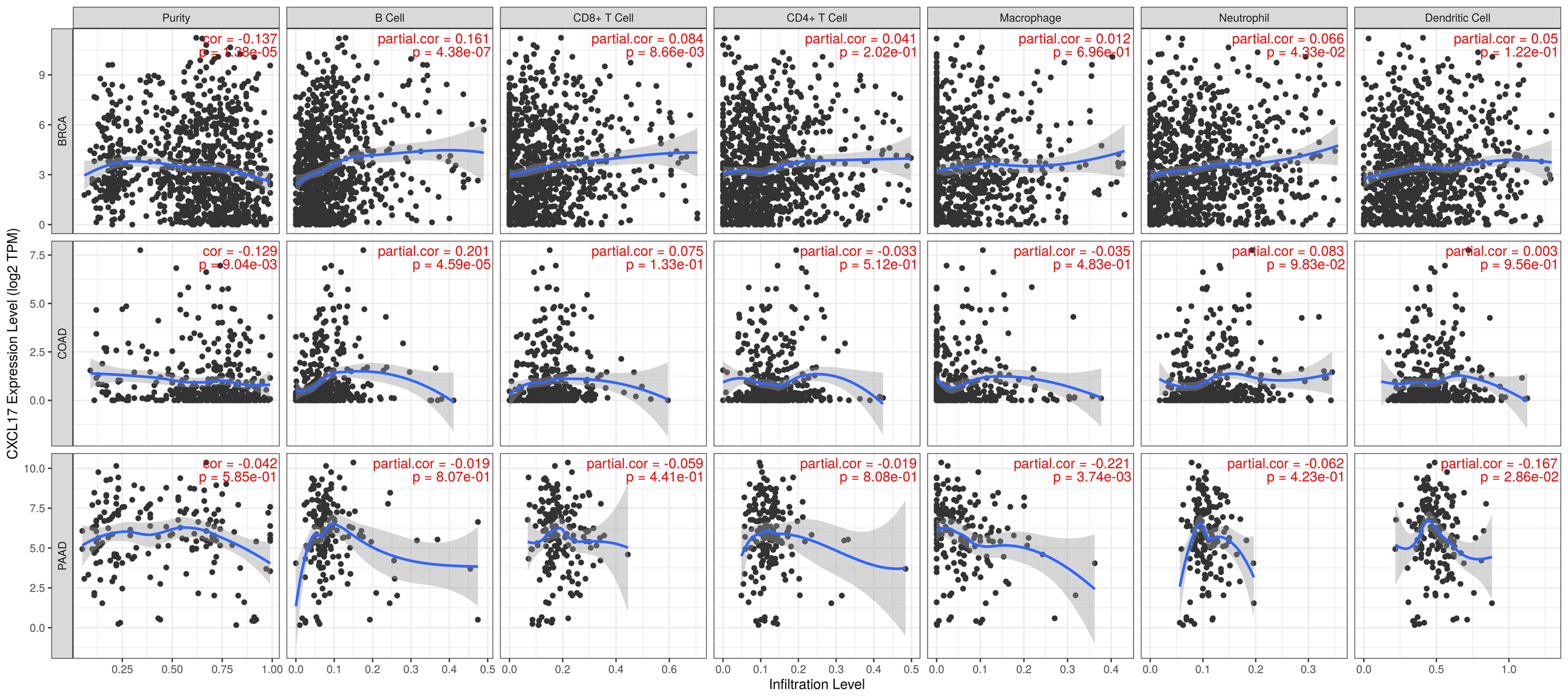

Supplement: Supplementary file 1 [file cancers-13-04153-s001.zip › Supplementary material/Material S6. Correlation of other differentially expressed CXCs and immune cell infiltration in BRCA, COAD, and PDAC/CXCL17.jpg]

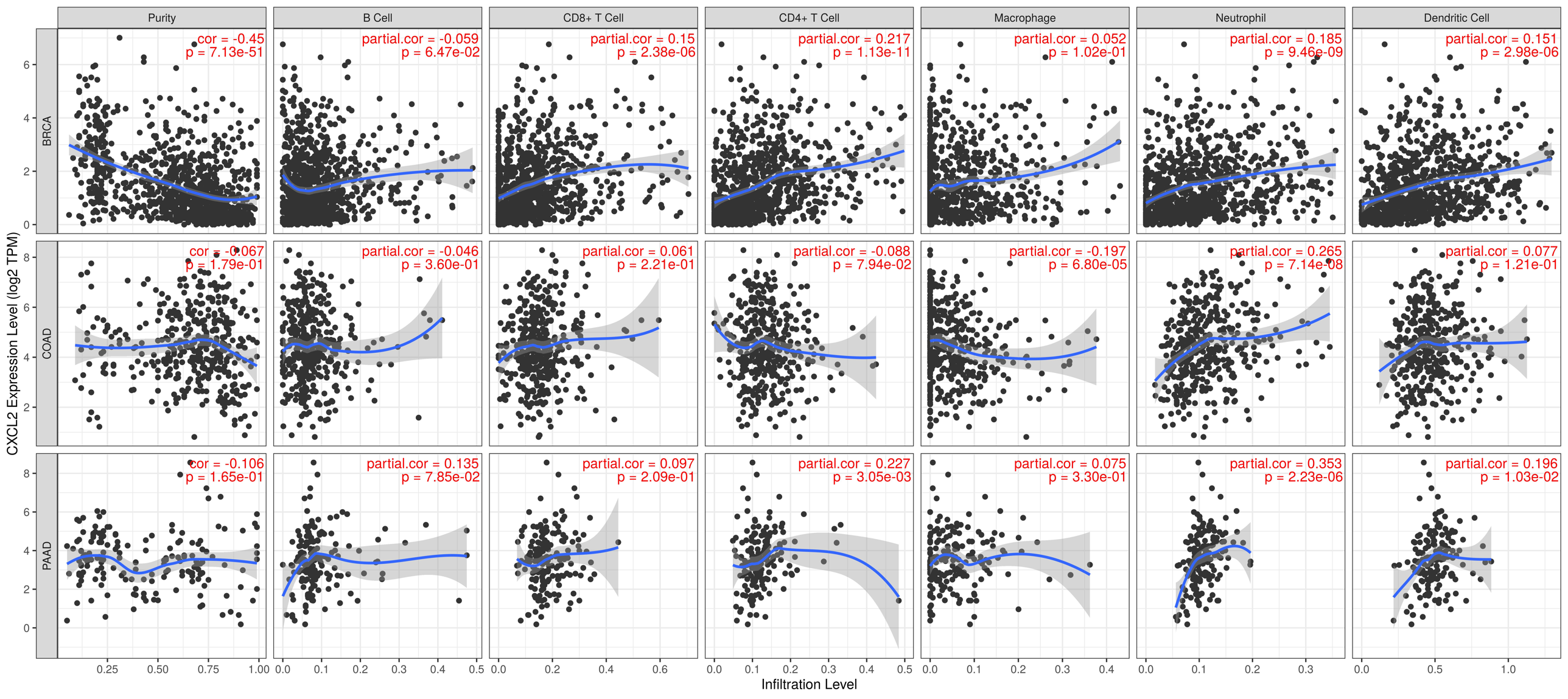

Supplement: Supplementary file 1 [file cancers-13-04153-s001.zip › Supplementary material/Material S6. Correlation of other differentially expressed CXCs and immune cell infiltration in BRCA, COAD, and PDAC/CXCL2.jpg]

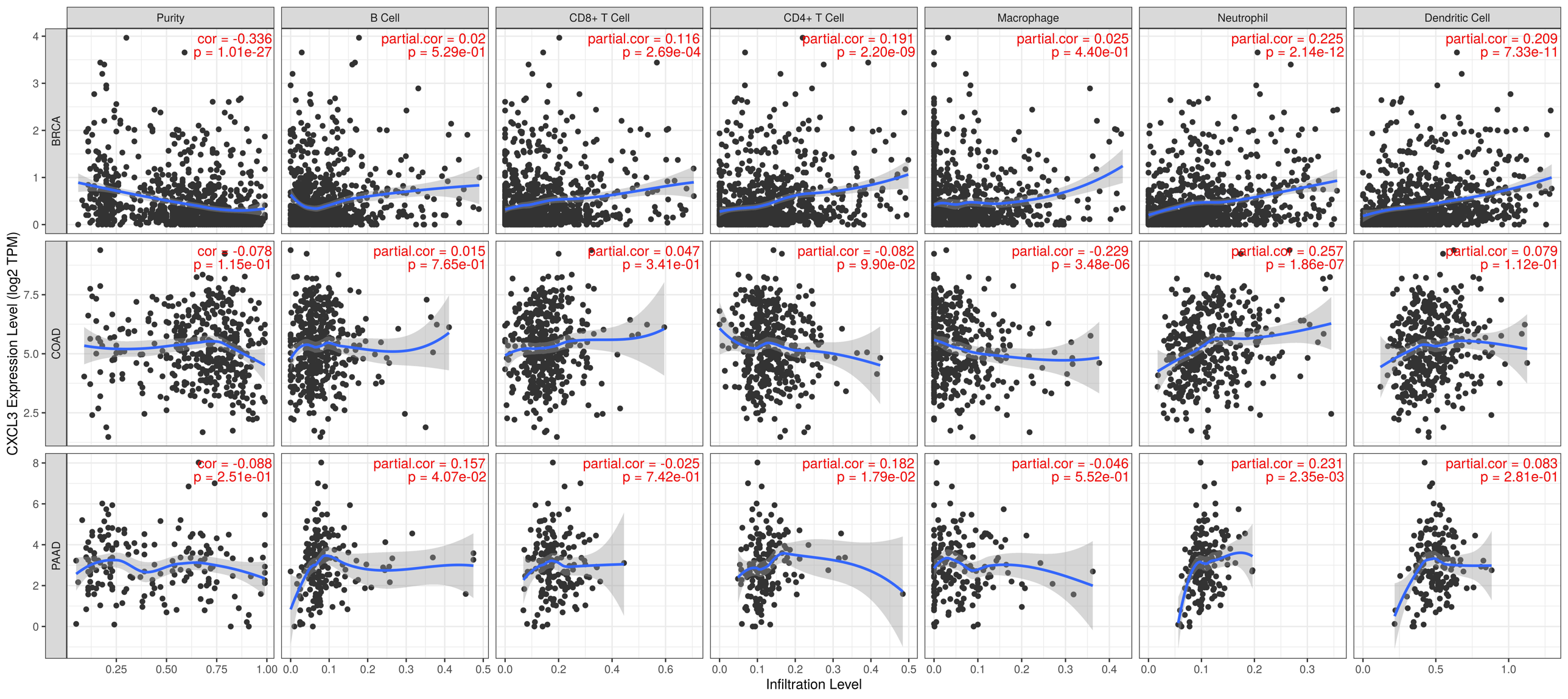

Supplement: Supplementary file 1 [file cancers-13-04153-s001.zip › Supplementary material/Material S6. Correlation of other differentially expressed CXCs and immune cell infiltration in BRCA, COAD, and PDAC/CXCL3.jpg]

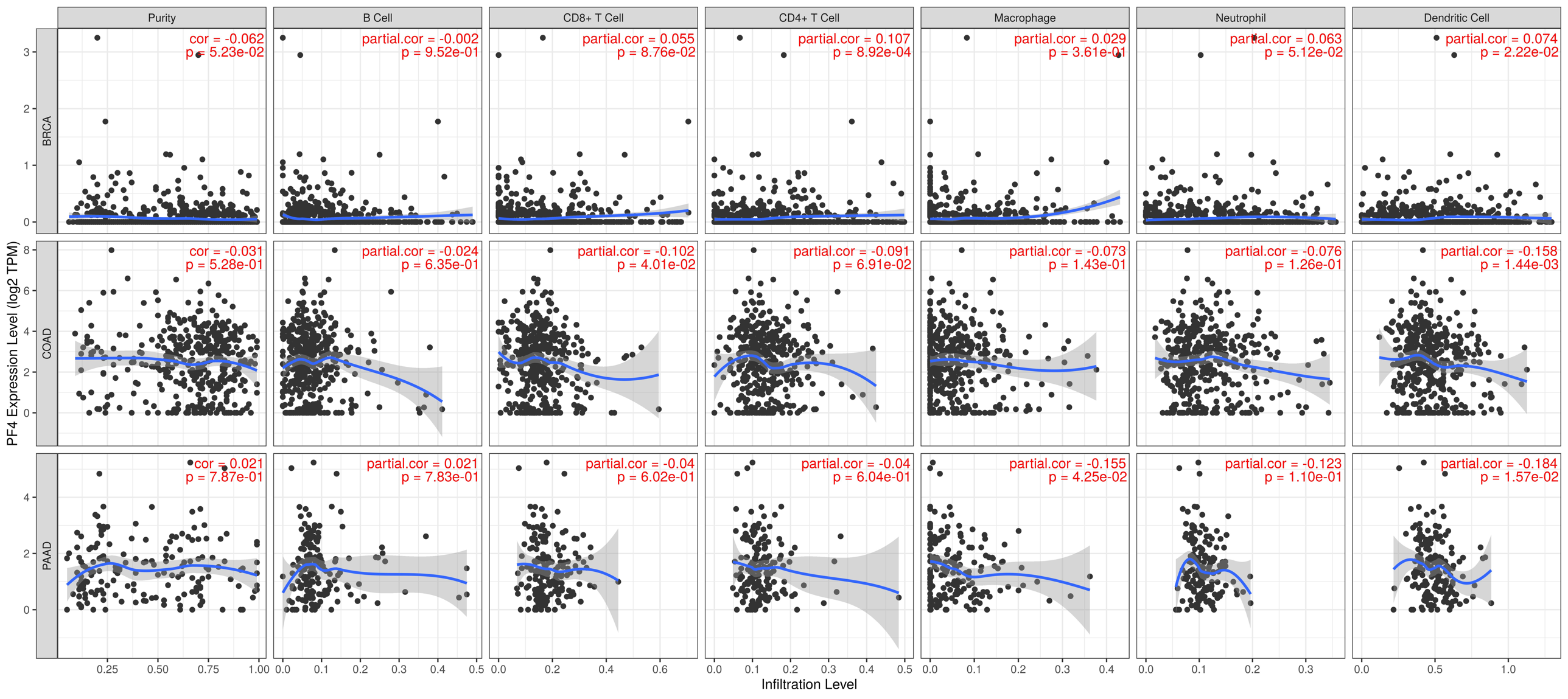

Supplement: Supplementary file 1 [file cancers-13-04153-s001.zip › Supplementary material/Material S6. Correlation of other differentially expressed CXCs and immune cell infiltration in BRCA, COAD, and PDAC/CXCL4.jpg]

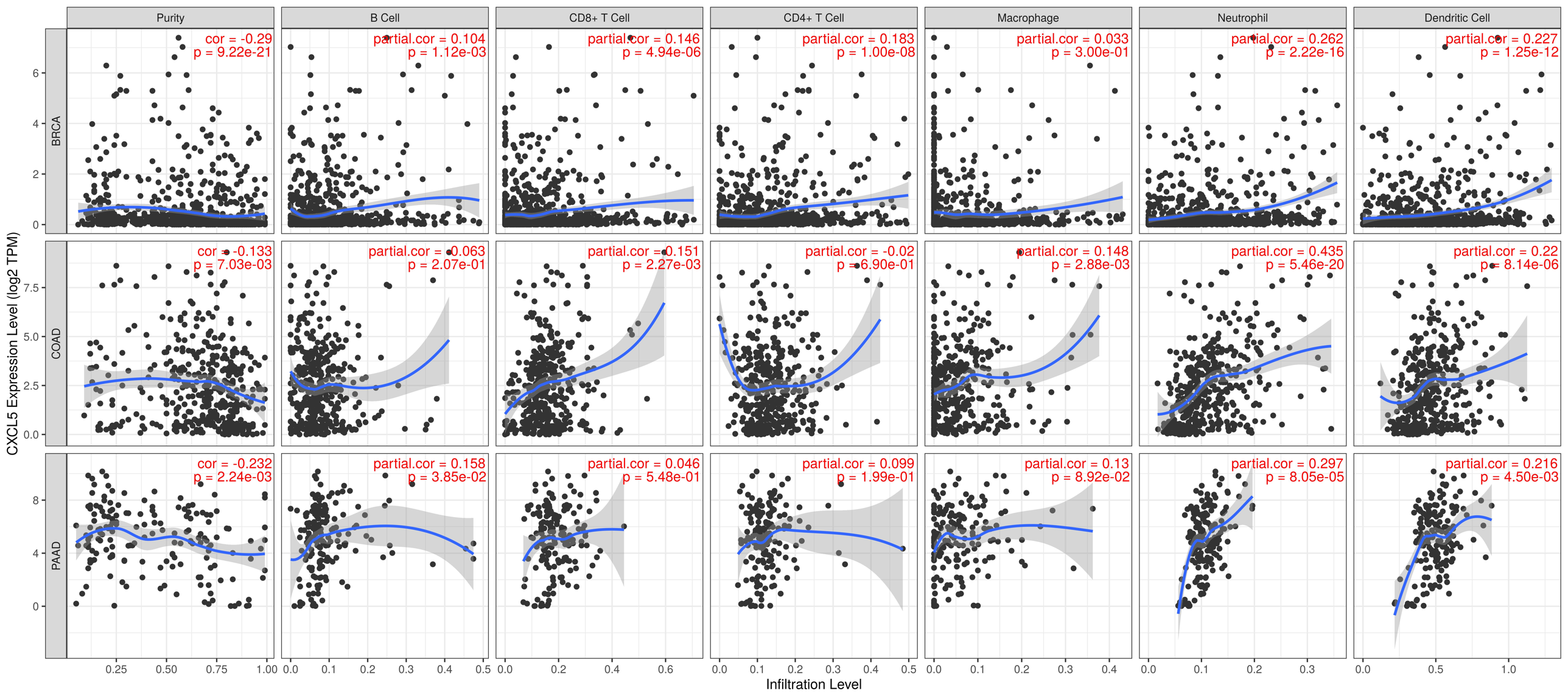

Supplement: Supplementary file 1 [file cancers-13-04153-s001.zip › Supplementary material/Material S6. Correlation of other differentially expressed CXCs and immune cell infiltration in BRCA, COAD, and PDAC/CXCL5.jpg]

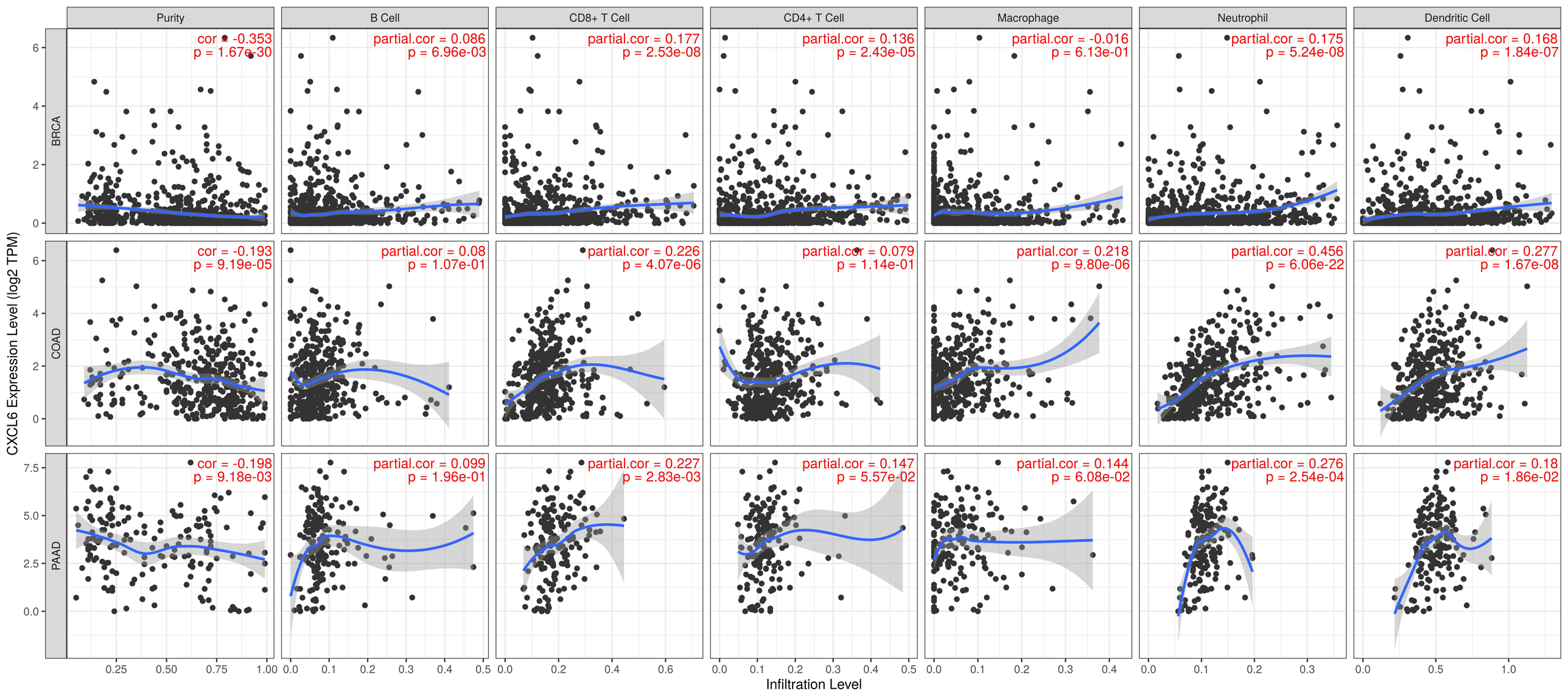

Supplement: Supplementary file 1 [file cancers-13-04153-s001.zip › Supplementary material/Material S6. Correlation of other differentially expressed CXCs and immune cell infiltration in BRCA, COAD, and PDAC/CXCL6.jpg]

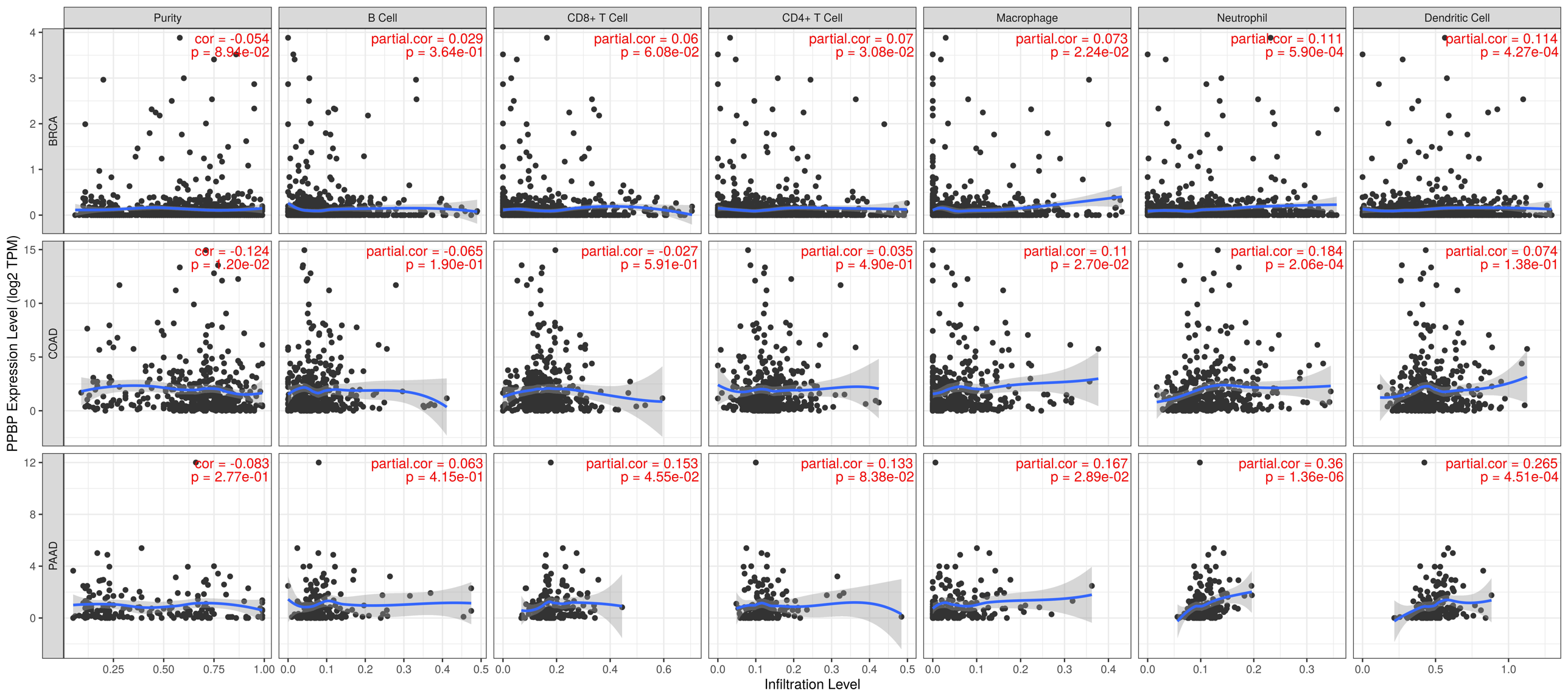

Supplement: Supplementary file 1 [file cancers-13-04153-s001.zip › Supplementary material/Material S6. Correlation of other differentially expressed CXCs and immune cell infiltration in BRCA, COAD, and PDAC/CXCL7.jpg]

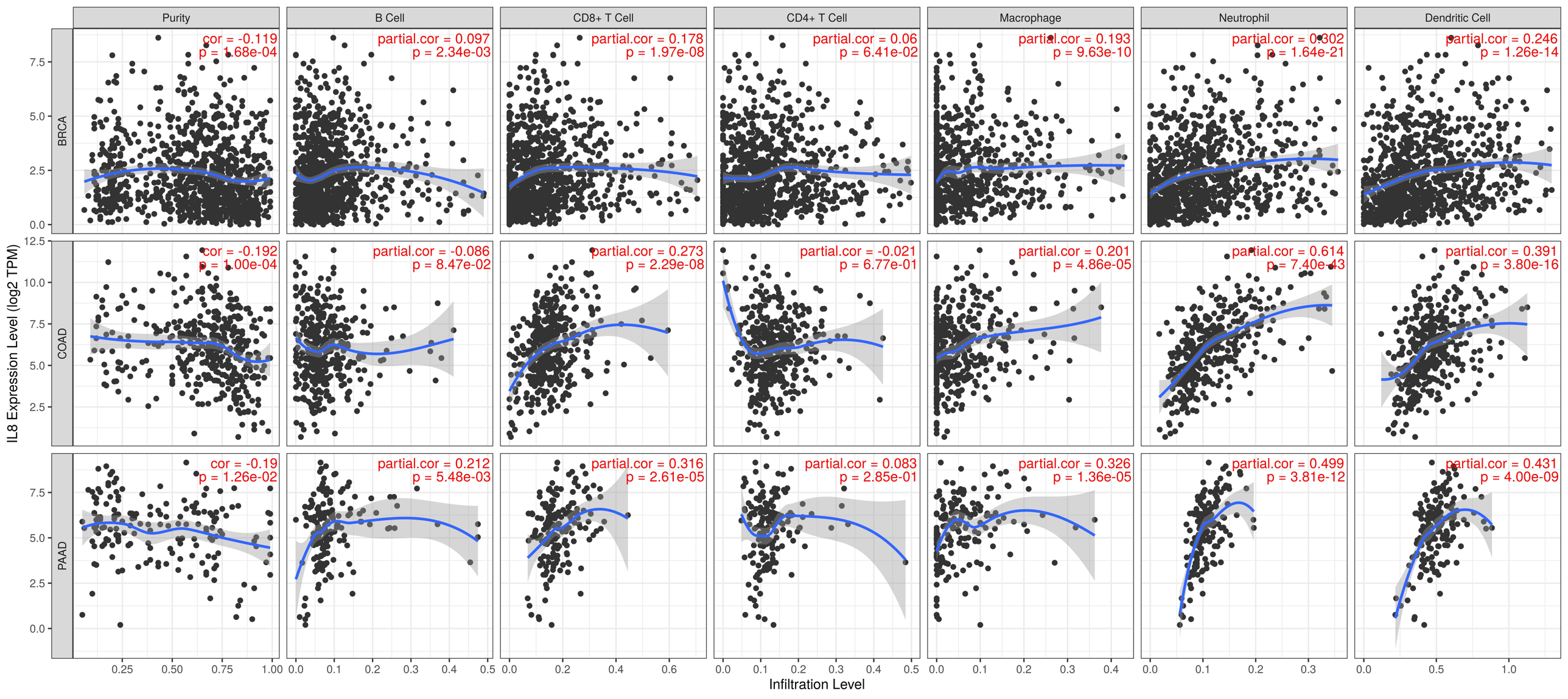

Supplement: Supplementary file 1 [file cancers-13-04153-s001.zip › Supplementary material/Material S6. Correlation of other differentially expressed CXCs and immune cell infiltration in BRCA, COAD, and PDAC/CXCL8.jpg]

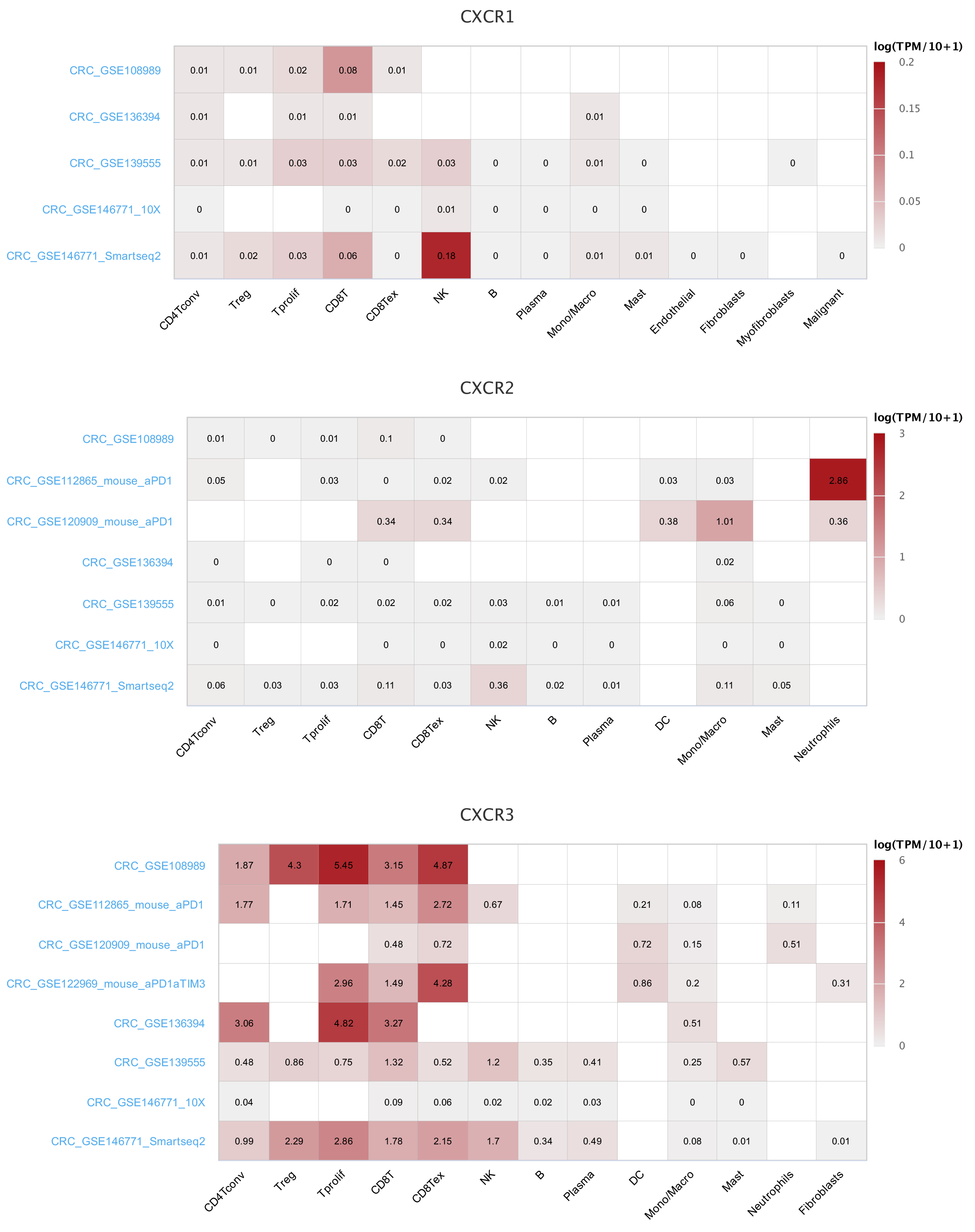

Supplement: Supplementary file 1 [file cancers-13-04153-s001.zip › Supplementary material/Material S7. Distribution of CXCRs in colon cancer/figure2-1.tif]

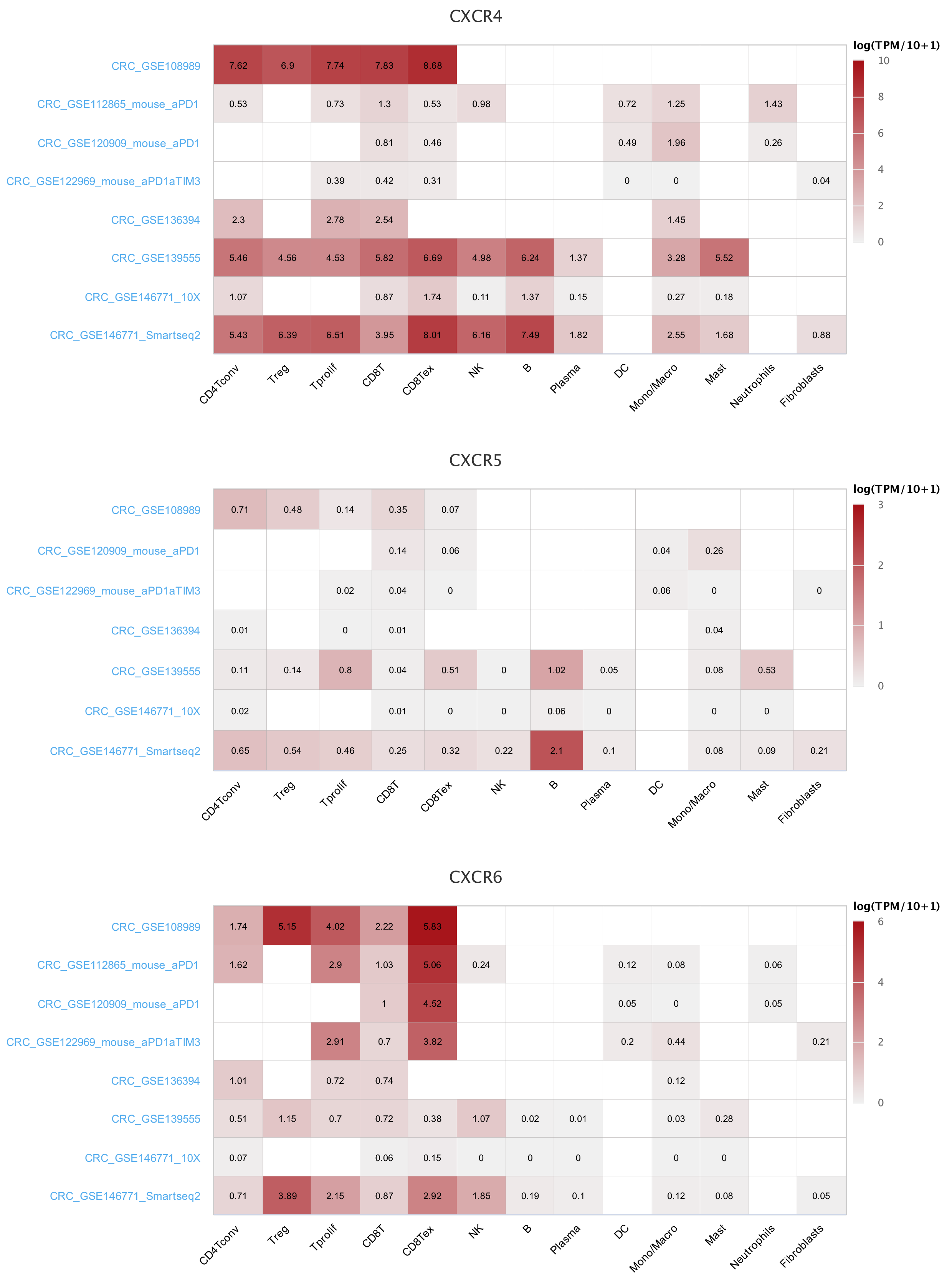

Supplement: Supplementary file 1 [file cancers-13-04153-s001.zip › Supplementary material/Material S7. Distribution of CXCRs in colon cancer/figure2-2.tif]

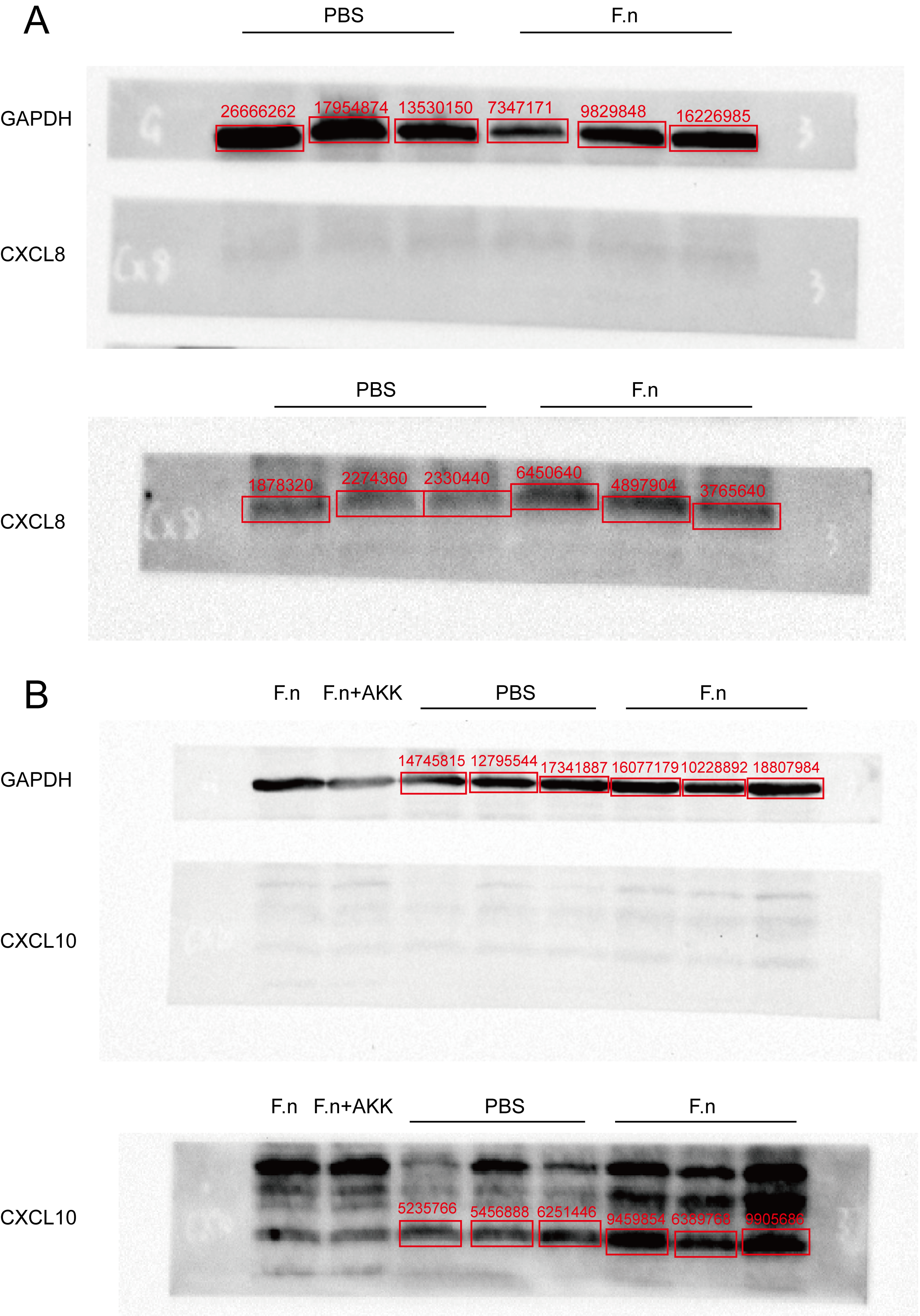

Supplement: Supplementary file 1 [file cancers-13-04153-s001.zip › Supplementary material/Material S8. The original Western blot images of (I&K)/Material S8.tif]
